# Temporal dynamics of oropharyngeal microbiome among SARS-CoV-2 patients reveals continued dysbiosis even after Viral Clearance

**DOI:** 10.1038/s41522-022-00330-y

**Published:** 2022-08-24

**Authors:** Suman Kalyan Paine, Usha Kiran Rout, Chandrika Bhattacharyya, Debaprasad Parai, Mahabub Alam, Rasmi Ranjan Nanda, Devashish Tripathi, Parveena Choudhury, Chanakya Nath Kundu, Sanghamitra Pati, Debdutta Bhattacharya, Analabha Basu

**Affiliations:** 1 National Institute of Biomedical Genomics, Kalyani, 741251 West Bengal India; 2 ICMR-Regional Medical Research Centre (Dept. of Health Research, Ministry of Health & Family Welfare, Govt. of India), Chandrasekharpur, Bhubaneswar, 751023 Odisha India; 3 KIIT School of Biotechnology, Campus XI, Patia, Bhubaneswar, 751024 Odisha India

**Keywords:** Metagenomics, Clinical microbiology

## Abstract

The severe acute respiratory syndrome coronavirus-2 (SARS-CoV-2) pandemic has posed multiple challenges to global public health. Clinical features and sequela of SARS-CoV-2 infection include long-term and short-term complications often clinically indistinguishable from bacterial sepsis and acute lung infection. Post-hoc studies of previous SARS outbreaks postulate secondary bacterial infections with microbial dysbiosis. Oral microbial dysbiosis, particularly the altered proportion of Firmicutes and Proteobacteria, observed in other respiratory virus infection, like influenza, has shown to be associated with increased morbidity and mortality. Oropharynx and lung share similar kinds of bacterial species. We hypothesized that alteration in the Human Oropharyngeal Microbiome in SARS-CoV-2 patients can be a clinical indicator of bacterial infection related complications. We made a longitudinal comparison of oropharyngeal microbiome of 20 SARS-CoV-2 patients over a period of 30 days; at three time points, with a 15 days interval; contrasting them with a matched group of 10 healthy controls. Present observation indicates that posterior segment of the oropharyngeal microbiome is a key reservoir for bacteria causing pneumonia and chronic lung infection on SARS-CoV-2 infection. Oropharyngeal microbiome is indeed altered and its α-diversity decreases, indicating reduced stability, in all SARS-CoV-2 positive individuals right at Day-1; i.e. within ~24 h of post clinical diagnosis. The dysbiosis persists long-term (30 days) irrespective of viral clearance and/or administration of antibiotics. There is a severe depletion of commensal bacteria phyla like Firmicutes among the patients and that depletion is compensated by higher proportion of bacteria associated with sepsis and severe lung infection from phyla Proteobacteria. We also found elevated proportions of certain genus that have previously been shown to be causal for lung pneumonia in studies of model organisms and human autopsies’ including Stenotrophomonas, Acenetobactor, Enterobactor, Klebsiella and Chryseobacterium that were to be elevated among the cases. We also show that responses to the antibiotics (Azithromycin and Doxycycline) are not uniform for all individuals.

## Introduction

The pandemicity caused by the severe acute respiratory syndrome coronavirus 2 (SARS-CoV-2) has impeded global public health. As of December, 2021, the COVID-19 pandemic has spread to 222 countries, resulting in over 260 million confirmed cases and over 5 million deaths globally (https://www.who.int/publications/m/item/weekly-epidemiological-update-on-covid-19–7-december-2021). The disease causes a wide range of symptoms, from moderate upper respiratory tract symptoms to severe acute respiratory distress syndrome. Recently, several studies have reported the role of microbiome in the COVID-19 associated complications, indicating probable links between COVID-19 and the oral, nasopharyngeal, gut and lung microbiome^1–3^. Homeostasis of pathogenic and symbiotic flora is an evolutionarily conserved phenomenon and dysbiosis is considered as a potential contributor and indicator of the disease. However, only a single study has reported the possible role of oropharyngeal microbiota in association with COVID-19 complications^4^.

Large-scale data on secondary bacterial Infections with SARS outbreaks postulate microbial dysbiosis among cases^5,6^. Oral microbial dysbiosis, particularly the altered proportion of Firmicutes and Proteobacteria, observed in another respiratory virus infection, like influenza, has shown to be associated with adverse response and increased morbidity and mortality^7^.

Among the most common long-term impact and adverse outcomes in SARS-CoV-2 infection are Sepsis, Pneumonia and other lung infections^8^. The clinical features of these ailments are often indistinguishable from a bacterial infection and the fact that these are often observed after the patient has eliminated the virus strongly indicates severe bacterial dysbiosis.

Cough, lung hypoxia, altered immune modulation through SARS-CoV-2 is likely to increase the risk of secondary lung infection and may favor the growth of anaerobes and facultative anaerobes. This evidence and the fact that the airways of the lung and oropharynx are closely connected led us to hypothesize that human oropharyngeal microbiome (HOPM) will be altered with SARS-CoV-2 infection and it will provide an indication of possible co-infections in the lungs.

Oral or salivary microbiome dysbiosis is well annotated in other reports for SARS-CoV-2 infection^7^. Oropharynx acts as a connecting link among nasopharynx and larynx and its microbiome is inhaled to the lung during breath-in of the respiration process. Inspite of having anatomical similarity between other oral cavity associate tissue and oropharynx, persistent nasopharyngeal inhalation and exhalation alter its environmental and chemical nature along with its microbial signature^9^. Previous studies documented that oral, oropharyngeal and lung microbiota share a similar pattern of bacterial species and amongst them the oral microbiome has higher diversity compared to lung microbiome^10,11^. Nasopharyngeal derived respiration process obstructs the airway passage of the oral cavity to the lung via oropharynx and may be the possible explanation for the similarity between oropharyngeal and lung flora and both being significantly distant from the oral microbiome (Supplementary Fig. 1). Data pertaining to oropharyngeal microbiome and its changes on SARS-CoV-2 infection is extremely limited. To the best of our knowledge, there has not been any longitudinal study on the dysbiosis of oropharyngeal microbiome of SARS-CoV-2 infected individuals. Our study is also unique as we have collected almost all our samples from home isolation, thus minimizing the confounding effect of hospital acquired bacteria.

Hence in the present longitudinal study, we mapped the human oropharyngeal microbiome (HOPM) at different time points (Day 1, Day 15 and Day 30) in SARS-CoV-2 infected individuals to understand the dynamics of microbiome dysbiosis. We observed striking contrast in the HOPM of infected individuals compared to uninfected controls even at Day 1 (at detection and completely treatment naive). At the phylum level, we identified a set of “marker bacteria” which show maximum perturbation (increase in Proteobacteria and severe decrease in Firmicutes) among the infected individuals compared to uninfected individuals. We also observed that once the homeostasis in the HOPM is perturbed, it is not easily restored, at least not in the 30 days time period that we have observed.

We found proportions of certain proteobacteria, that have previously been shown to be causal for lung pneumonia in studies of model organisms and human autopsies, like Stenotrophomonas, Acenetobactor, Enterobactor, Bifidobacterium and Chryseobacterium to be elevated among the cases. We also show that responses to the antibiotics (Azithromycin and Doxycycline) are not uniform for all individuals. Although we observed reduction in the proportion of harmful bacteria for some individuals on administration of the antibiotics; for others, especially ones who initially had very low proportion of the harmful bacteria, the trend often reversed.

## Results

### Demography of study participants

**Fig. 1:**
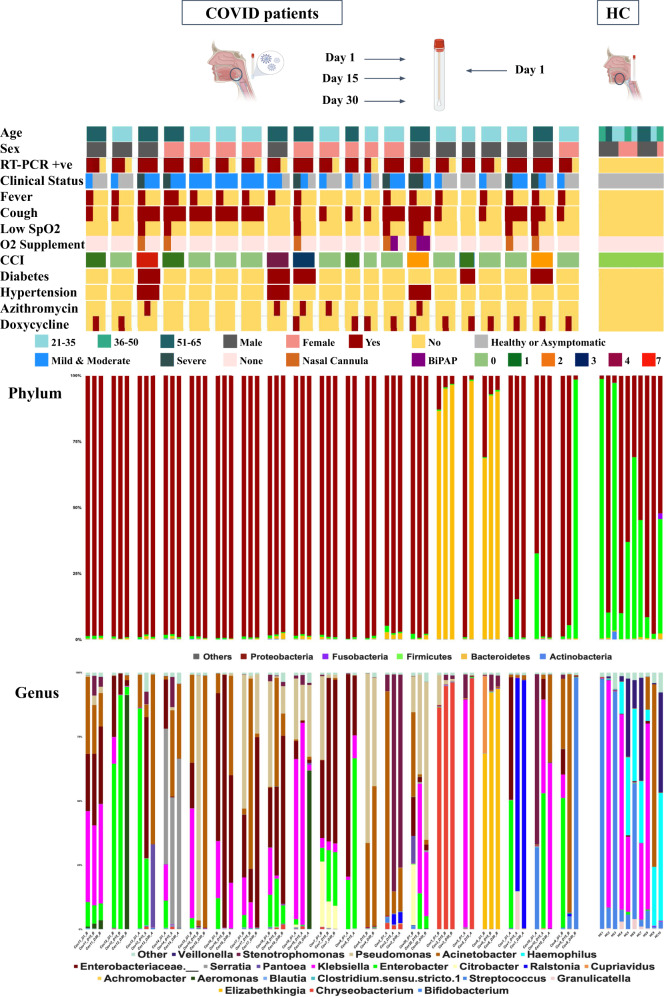
Schematic representation of the study design. Demographic characteristics (age and sex), Charlson Morbidity Index (CCI), RT-PCR positive, drugs intake (Azithromycin, Doxycycline) and respiratory support among Covid-19 patients (cases) are described with color legends. Patients’ throat swabs were collected at three distinct time points: Day 1, Day 15 and Day 30, as well as healthy controls (HC) on Day 1. Bacterial composition at the phylum and genus level in both cases and HC are depicted here. (Created by BioRender).

In (Fig. 1), we have summarized the phenotypic and microbial diversity data of study participants. The study included two different groups, where Group A (*n* = 8) is defined as the individuals who are RT-PCR positive 30 days after diagnosis and individuals who become negative within the 30 days time frame, after diagnosis, defined as Group B (*n* = 12). Among all the participants (*n* = 20), 16 individuals were administered antibiotics. Azithromycin was administered on 5 individuals and Doxycycline was administered on 11 individuals. Two individuals received both antibiotics. Two individuals had to be supported by external oxygen supplementation. Comorbidity status were estimated through Charlson Comorbidity-index (CCI)^12^. All of the above clinical characterizations are indicated in (Fig. 1).

### Distribution of phylum among Cases and Controls

**Fig. 2:**
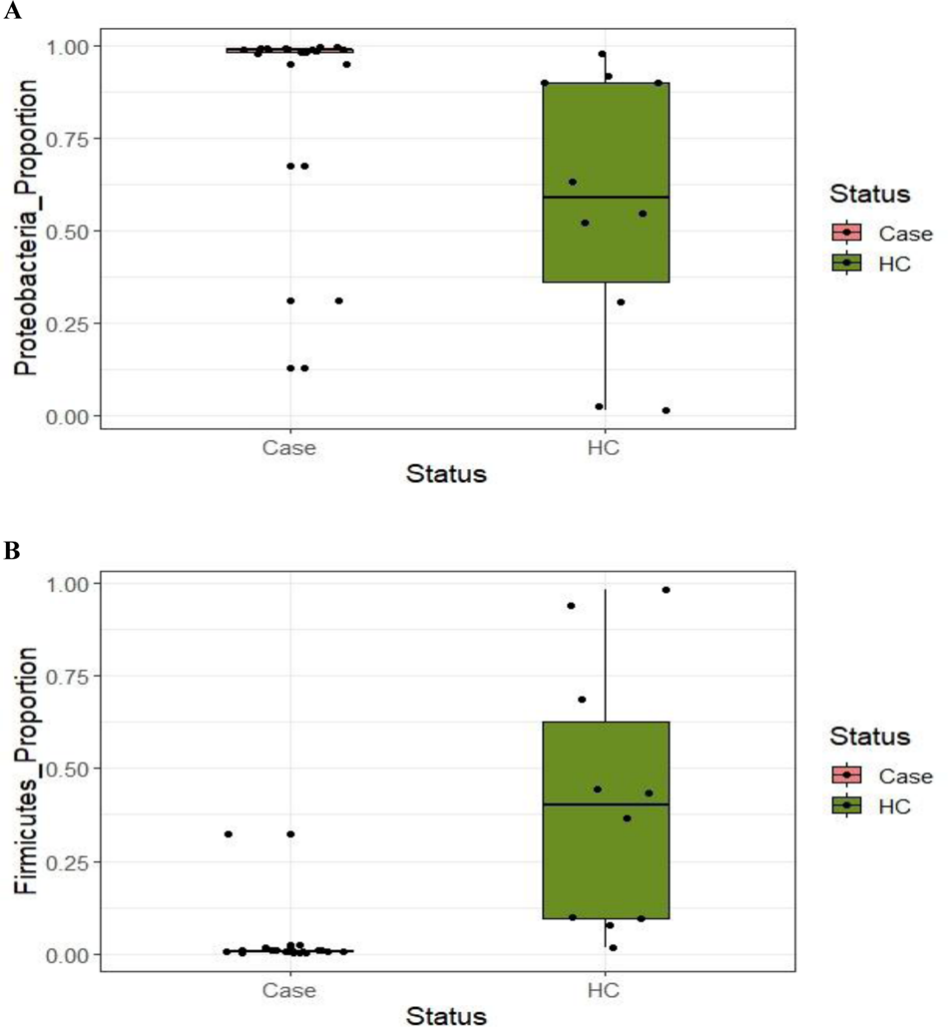
Box-whisker plots of key phyla among cases and HC. Distribution of key phylum Proteobacteria (**A**), Firmicutes (**B**) and Fusobacteria (**C**) among cases (Day-1) and control.

We have used Hotelling’s T^2^ Statistics to show that the distribution of the phyla among cases in Day 1 is significantly different from HC (Value of Test Statistic: 31.725, Numerator df, Denominator df: (5, 23, *p* value: 0.00197). Comparing different phylum, we found increased proportion of the key phyla like Proteobacteria among the cases [Cases:0.89 ± 0.25;range:0.12–0.99; Control:0.57 ± 0.36;range:0.013–0.98] whereas Firmicutes (Cases:0.026 ± 0.07;range:0.002-0.32; Control:0.41 ± 0.36; range:0.019–0.98) were high among HC (Fig. 2). Additionally we observed the difference in distribution among the other phyla that includes Bacteroidetes (Cases:0.085 ± 0.24;range:0.00008–0.86; Control:0.005 ± 0.006; range:0.0009-0.022;), Actinobacteria (Cases:0.0016 ± 0.0007; range:0.0003–0.0035 Control:0.004 ± 0.009; range:0.0000955–0.03) and Fusobacteria (Cases: 0.0000159 ± 0.0000194; range:0.0–0.00006; Control:0.002 ± 0.006; range: 0.0000352–0.02) between cases and controls.

### Alpha and Beta diversity in Cases and Controls

**Fig. 3:**
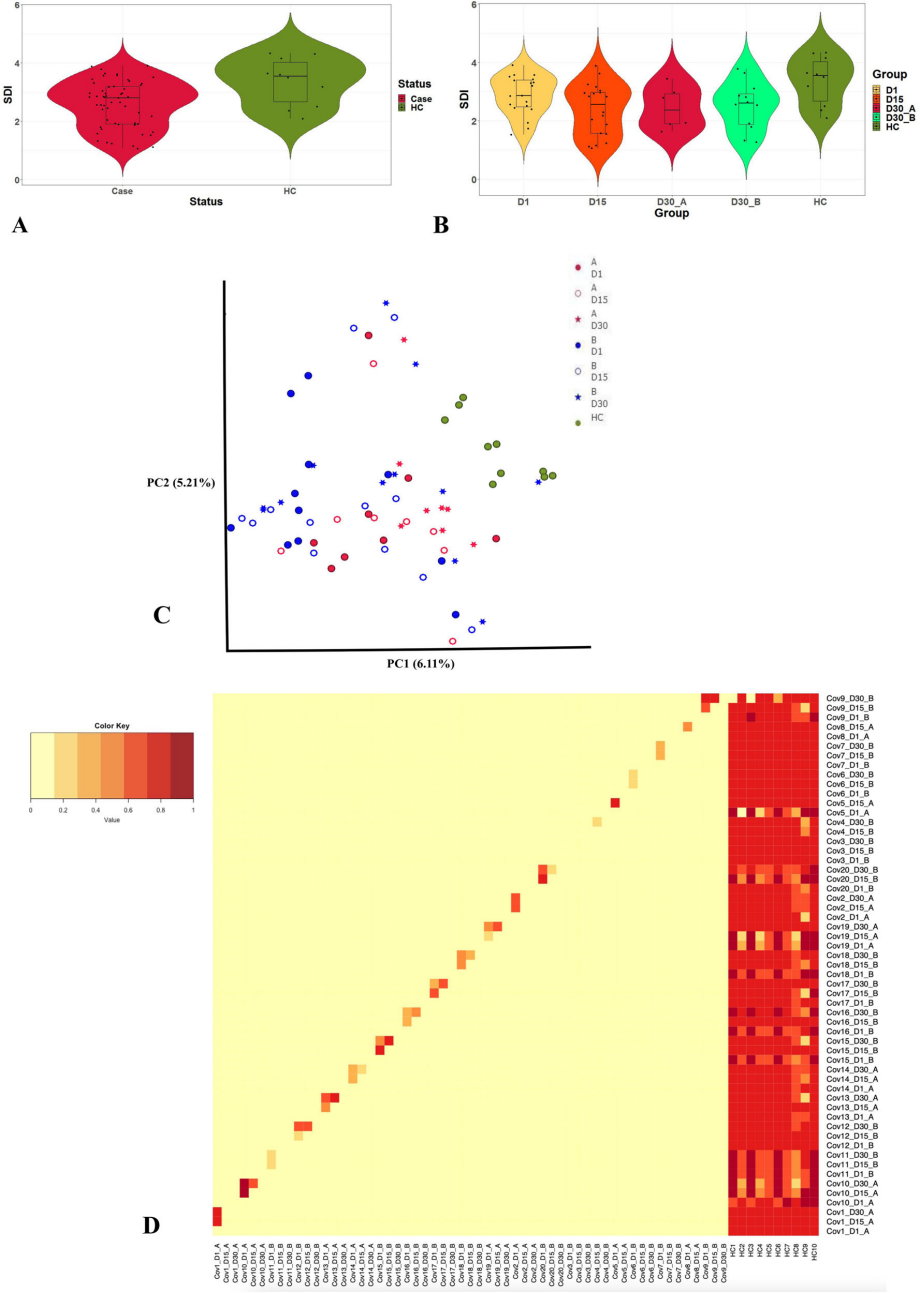
Detailed profile of microbial diversity. **A** Alpha-diversity (richness) among the Covid-19 cases and healthy controls (HC) were estimated through Shannon diversity index (SDI). The SDI among the cases and HC were depicted through violin plots. **B** Violin plots of SDI between cases and HC at different time points, such as Day 1, Day 15, and Day 30. The cases of Day 30 were divided into two distinct sub-groups: Group A, RT-PCR positive, and Group B, RT-PCR negative. **C** The principal components (PC1 vs PC2) plot is based on the Jaccard diversity index and demonstrates microbial clustering among groups (Aand B) across different time points (Day 1, Day 15 and Day 30) as well as HC. **D** Heat map showing the intra-individual beta diversity of the human oropharyngeal microbiome among groups estimated through Bray-Curtis Diversity Index.

We compared Shannon’s diversity indices (SDI) and found significant differences between cases and controls (Case: 2.58 ± 0.8, Control: 3.4 ± 0.8 K-S *p*-value: 2.2e–16), indicating reduced diversity and stability of HOPM among cases (Fig. 3A). We have computed the alpha diversity pattern with different time points that includes Day 1, 15 and 30 and the cases are further categorized with Group A and Group B who become RT-PCR positive at Day 30 and RT-PCR negative respectively (Fig. 3B). When we further subdivide the cases and calculate the alpha diversity for the three time points, we observe very little change Day 1 (Mean ± SD: 2.9 ± 0.6, range: 1.5–3.9), Day 15 (Mean ± SD: 2.4 ± 0.9, range: 1.05–3.9) and Day 30 (Mean ± SD: 2.5 ± 0.74, range: 1.27–3.8) in SDI over the tree time points. Further, in accordance with the expectation, the SDI among Group A (Mean ± SD: 2.45 ± 0.7, range:1.6–3.4) in Day 30, who were also found to be RT-PCR positive at Day 30, was lower compared to Group B (Mean ± SD: 2.6 ± 0.8, range:1.26–3.8).

We computed the pairwise distance between all possible pairs of individual using the Jaccard diversity index and plotted the first two principal components (Fig. 3C). The HC separates from the cases and forms a clear cluster, although no apparent clustering was observed for any other group. The intra-individual beta-diversity of HOPM, was measured using BCDI (Fig. 3D). Of all the groups, the BCDI is the least among HC, indicating that the microbial diversity is more homogeneous among the controls. Among cases, we found that BCDI was significantly different between Group A and Group B, i.e. the people who were RT-PCR positive and negative; at Day 30 time point, (r^2^:0.03; p-value: 0.006 using two-way PERMANOVA); but the difference was not significant at Day 1 and Day 15.

### Temporal variation of Genus in SARS-CoV2 infection

**Fig. 4:**
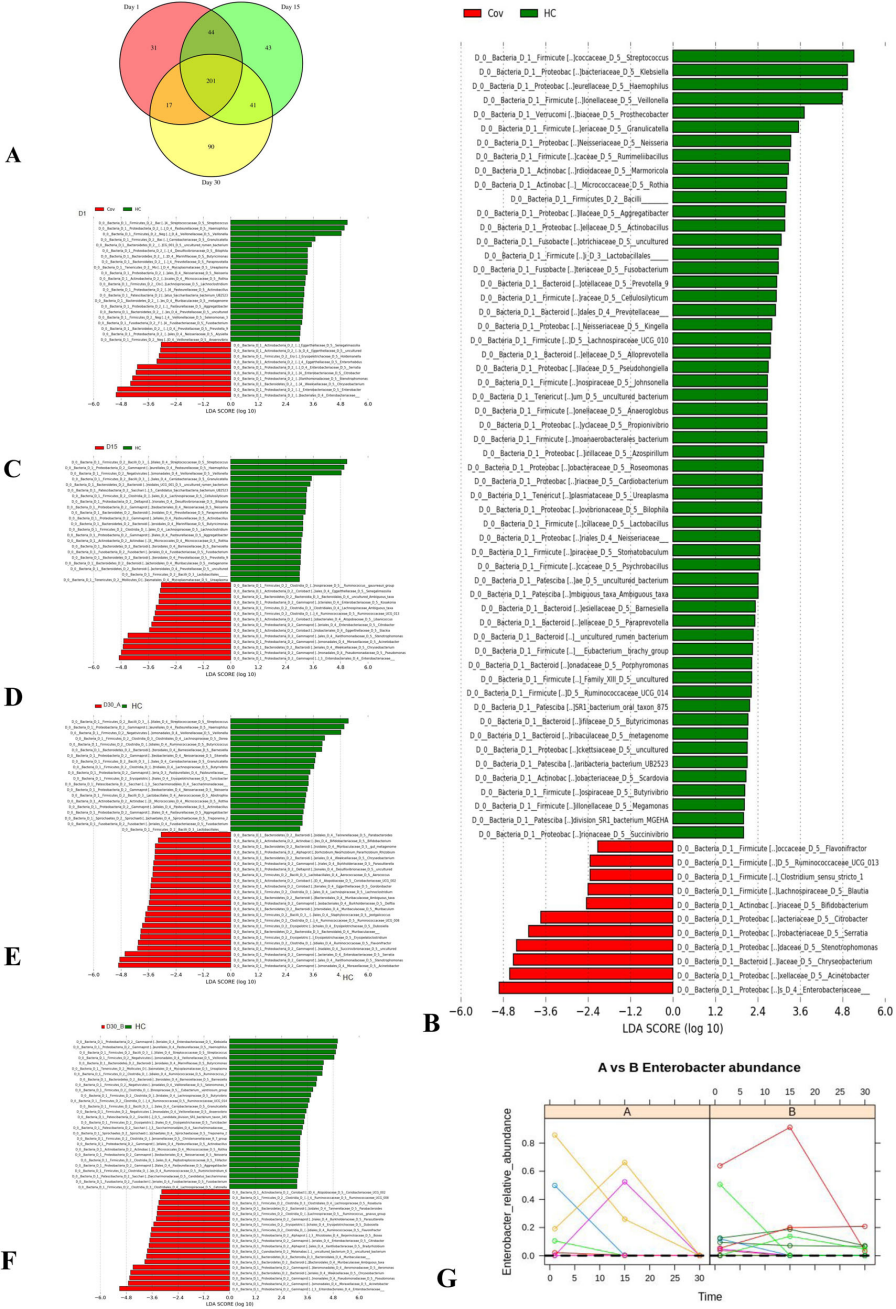
Detailed description of key OTUs discriminating study groups. **A** Venn diagram documented that 201 of 512 OTUs were common for both case and control groups. **B** Bacterial taxa of human oropharyngeal microbiome (HOPM) were identified using Linear Discriminant Analysis (LDA). Fifty-nine taxa were significant among cases and controls (LDA score > = 2.5). **C**, **D** Bacterial taxa ofthirty-one and thirty-sixwere significant among cases in the different time points Day 1, and HC and Day 15 and HC using LDA(LDA score > = 3). **E**, **F** Distinct bacteria taxa of forty-three and forty-seven were identified between Group A and HC as well as Group B and HC(LDA score > = 3). **G** Microbiome Interpretable Temporal Rule Engine (MITRE) algorithm found that the Enterobactor genus distinctly separates Group A and B.

Altogether we found 511 genus-specific OTUs, if we combine all the time points (Day 1, Day 15 and Day 30) and both cases and controls (Supplementary Table 2). Of these, among the cases, 31 OTUs were uniquely present in Day 1, 43 OTUs unique for Day 15 and 90 unique to Day 30 (Fig. 4A; (Supplementary Table 3)). We identified 97 genus-specific OTUs that are present only in HC and not detectable among affected individuals. Using Linear Discriminant Analysis (LDA), we identified a total of 59 OTUs of HOPM that can significantly separate the cases and controls; (Fig. 4B). We used LDA to find the OTUs that were significantly different between HC and the cases at different time points (Day 1, Day 15 and Day 30). A complete list of OTUs that came out to be significantly different are documented in (Fig. 4C–F). The OTUs which have strong evidence to be causal or have previously shown to be strongly associated with lung infection are mentioned in (Supplementary Table 4, Supplementary Fig. 2) along with the supplementary notes. In general, we have observed different kinds of potentially harmful bacteria in high proportion among the cases, particularly among Group A. The detailed functions and references of the lung-infection-associated-OTUs are in the Discussion section. Using a the supervised machine learning method, MITRE (Microbiome Interpretable Temporal Rule Engine)^13^ for feature selection on our longitudinal data, we found that Enterobactor is the most important candidate OTU to differentiate between Group A who harbor virus for longer period of time (>30 days) compared to Group B who eliminate the virus in shorter period (Bayes factor 0.516) (Fig. 4G). It is to be noted here, that in one individual (ID Cov 20), from whom we have collected samples during his hospital stay, a distinct pattern, where four OTUs: Chromobacterium, Novispirillum, Rickettsia and Bryobacter, were observed.

**Fig. 5:**
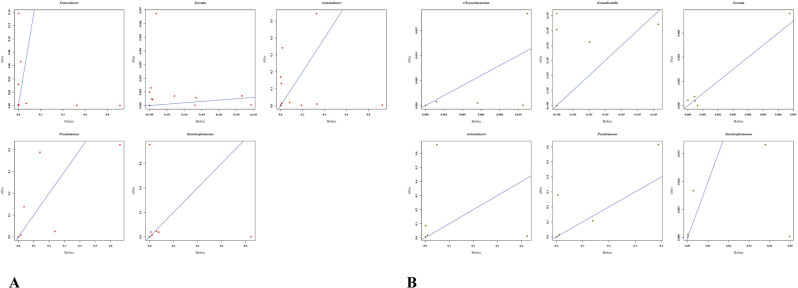
Treatment responses of antibiotics among cases. **A** Doxycycline, **B** Azithromycin. Proportions of key OTUs are plotted for individual cases before and after administration of antibiotics.

We have further selected 22 key genus, that are associated with cases in the discriminant analysis, for exploring their pattern with the use of antibiotic. We compared the microbiome profile, ~10 days after the completion of therapy and compared it with the profile prior to administration of antibiotics. The SDI did not alter significantly upon antibiotic administration (Supplementary Fig. 3 and supplementary note). Both antibiotics exhibit low efficacy in terms of proportional reduction for the bacteria Acenetobacter, Pseudomonas and Granulicatella which are associated with lung pneumonia. The study reveal that the Azithromycin administration could not uniformly reduce opportunistic pathogens like Stenotrophomonas, (correlation coefficient: 0.28), Serratia (correlation coefficient: 0.99), Chryseobacterium (correlation coefficient: 0.57) and only 40% individuals responded to the therapy (Fig. 5B). It may be noted that correlation here evaluated on the proportion of bacteria before and after the antibiotic treatment. Only one individual responded to Azithromycin. Reduction of proportion of the genus Bifidobacterium and Clostridium was documented among all 11 individuals who were administered Doxycycline. More than 70% of individuals did not show any reduction in proportion of Acenetobacter, Enterobacter, Serratia, Stenotrophomonas and Pseudomonas upon antibiotic administration (Fig. 5A). However, for both the antibiotics, the proportion of Streptococcus and Haemophilus, that is in high proportion among HC, increased among cases upon administration.

## Discussion

We quantitatively documented the significant temporal dysbiosis of human oropharyngeal microbiome (HOPM) community structure for SARS-CoV-2 infection. As the incubation period of SARS-CoV-2 infection is variable and infection generally occurs a few days before the appearance of symptoms; we have collected the microbiome samples immediately after the development of symptoms and RT-PCR confirmation for an individual to minimize the interval between infection and data collection. Our study revealed that comprehensive HOPM dysbiosis occurs rapidly, i.e. within a few days upon infection of SARS-CoV- 2, whereas the extent of dysbiosis increases with time and persists at least for a month, irrespective of whether the virus has been cleared. The study documented that entropy, measured using SDI, among COVID cases decreased significantly compared to HC. Further, the SDI is at its lowest after 30 days of infection, even upon clearance of infection. Interindividual distance (BCDI) of HOPM, increases monotonically from Day 1 to Day 30 among cases and is significantly lower among the controls. This phenomenon is unique to SARS CoV2 and different from other respiratory viral infections like H1N1, where the reshaping and restoration of the HOPM occurs faster. This is suggestive that the chances and risk of lower respiratory tract bacterial infection and its complications can be long-term in SARS-CoV-2 infection. Like previous reports we found some difference in HOPM between short (in our case group B) and long SARS-CoV-2 infection (group A), but unlike the previous study, we could report that the dysbiosis persists even in Group B, i.e. even after viral elimination^14^. Long-term lung infection-related complication and post infection hospitalization also indicates prolonged HOPM dysbiosis as a potent contributor for the same^15,16^. Clinical observations like the occurrence of bacterial co-infection as an adverse complication of SARS-CoV-2 infection, repeatedly reported in several studies bolsters the possibility of pneumonia-associated pulmonary complication even after viral elimination in recovery phase^17–19^.

In the present study we found that the bacterial genus Acenetobactor, Chryseobacterium, Stenotrophomonas, Serratia and Enterobacter are significantly associated with SARS-Cov-2 infection with the effect size of >4.5 (LDA Score). The genera Enterobactor is usually nosocomial, but there is evidence of its presence in high proportion in transtracheal aspiration among the fatal pneumonia patients^20^. We observed the co-existence and increased proportion of Enterobacter, Klebsiella and Acenetobacter among the severe cases. It is to be noted here that bacteria Enterobacter and efficiently can grow in hypoxic condition among all the severe cases who had SPO2 < 90. The genera Klebsiella, Acenetobactor^21,22^ and Chryseobacterium^23,24^ has previously been identified as potential players for pulmonary pneumonia. Previous reports from postmortem samples reveal that bacteria like Stenotrophomonas^25,26^ and Serratia^27–29^, which we found significantly higher in Group A compared to HC, are causal and is found in hemorrhagic bronchopneumonia and diffuse neutropenic pneumonitis resembling diffuse alveolar damage with pulmonary hemorrhage. Similar anatomical aberrations were also reported from SARS-CoV-2 infected catastrophic samples^30^. The genus Bifidobacterium, whose abundance has been observed among all cases may potentially contribute to inflammatory oropharyngeal scar via bifid-shunt^31,32^. Blautia was previously documented as the dysbiosis marker for intestinal microflora among the subjects acquiring inflammatory host physiology^33^ and here we found it as a potential marker for HOPM dysbiosis. The genus Blautia are thought to activate systemic inflammation upon SARS-CoV-2 infection that induce Pathogen associated Molecular Pattern (PAMP) are also present in severe cases with limited abundance^34^. In the present study, we have also documented unique presence of certain genera only in the hospitalized patient. These OTUs Chromobacterium, Novispirillum, Rickettsia, Bryobacter, are known to be hospital contaminants; indicating possible artifacts during our hospital collection. A recent similar study has documented the positive correlation between HOPM dysbiosis and local inflammation. Previous functional studies have shown that Gordonibacter, Lachnoclostridium and Clostridium, are immune mediators of inflammatory disease or activators of inflammation^35^. We found them in high proportion among the RT-PCR positive cases Group A at Day 30. The genus Aerococcus and Delfia are considered as potential opportunistic pathogens for pulmonary and cavitary lung infection^36^. These bacteria are also present in Group A but not in Group B on Day 30. On the other hand, we found reduction in proportion of Streptococcus and Velionella among cases compared to control. Streptococcus and Velionella together regulate the biofilm formation at oropharynx through quorum-sensing (QS) that are essential for the prevention of infectious diseases^37^. Altered homeostasis by the depletion of symbionts of HOPM may be one possibility for the long term dysbiosis observed in SARS-CoV-2 infection even after elimination. The dysbiosis of HOPM may alter respiratory epithelium by inflammatory cytokines and promote the adhesion of respiratory pathogens that are putative to develop pneumonia-like conditions on SARS-CoV-2 infection. Similar epithelial layer, airways connection and recent culture-independent NGS techniques have demonstrated an oral-lung axis, where similar patterns of microbial ecosystem persists^10^. Microaspiration seems to be one plausible reason for the homogenous microbiome profile between these two organs (lungs and oropharynx). It is also hypothesized that oropharynx have the highest impact for microbial transfer from oral to lung axis^38^. Anterior segment of oral cavity, tongue and saliva significantly differed with oropharynx in terms of host environment. The pH of oropharynx (pH ~ 5.6) significantly differed with the anterior segment (pH~6.4-7.5)^39^. Apart from the chemical nature, airway passage and oxygen content also differed between anterior and posterior segment of oral cavity which are crucial for microbial architecture. Oropharynx airway passage mediated through nasopharynx which obstructed from the anterior segment of the oral cavity to inhale during the process of respiration (Supplementary Fig. 1). Microaspiration of nasopharyngeal air along with the microflora passed through oropharynx to lung for respiration and it may be the possible explanation of the phenomenon that oropharynx shared a similar pattern of lung microbiome compared to anterior segment. Our study looks into the temporal variations of oropharynx to speculate the lung microbiome dysbiosis. Because of the similarity between the microbiome of the organs we think its an efficient and minimally invasive way to understand microbial dysbiosis in the lung.

We found antibiotic administration (both Azithromycin and Doxycycline) was effective on limited bacterial genus and a limited number of individuals. However, the genus Streptococcus and Velionella that are abundant in HC, has increased its proportion in individuals after antibiotic treatment. This may reflect a path to recovery and healthy HOPM restoration. Azithromycin treatment reduced the proportion of opportunistic pathogens like Enterobacter, Haemophilus and Doxycyclin reduced the proportion of Clostridium. The study reveals that neither of the antibiotics are able to reduce the opportunistic pathogenic bacteria uniformly. The proportions of Pneumonia associated bacterial genus, including Acenetobactor, Blatulia, Pseudomonas, Aeromonas, Klebsiella, Enterobactor and Chyroseobacterium were not altered post-administration of antibiotic Azithromycin and Doxycycline; although these bacteria were present in almost 80% of cases (n = 16). Alternative antibiotic therapeutic regime to uniformly reduce the risk of secondary bacterial infection among SARS-CoV-2 patients is necessary.

Dysbiosed HOPM has long been recognized as a critical determinant for the development of lung infections as well as its complications^40,41^. Multiple reports postulate that oral hygiene interventions among pneumonia patients leads to a quicker and higher recovery rate^42–44^.

Our study underscores the importance in identifying the HOPM dysbiosis bacterial markers during SARS-CoV-2 infection and the nature of HOPM dysbiosis to formulate the chemotherapeutic strategy to reduce early and late onset of lung infection related morbidity and mortality.

## Methods

### Ethical approval

Written informed consent was obtained from all the study participants after receiving the Institutional ethics committee recommendation from both the institute: National Institute of Biomedical Genomics, Kalyani and ICMR-Regional Medical Research Centre, Bhubaneswar.

### Study participants

20 cases were enrolled for the study and followed-up for one month. Oropharyngeal swabs were collected and RT-PCR was done at three time points: Day1 (after RT-PCR confirmation), Day15 and Day 30 after first diagnosis through RT-PCR for SARS-CoV-2. It may be also noted that the first diagnosis was done immediately after flu-like symptoms that include fever and/or cough, developed in an individual. Oropharyngeal swabs were collected within 24 h of RT-PCR confirmation. Except in one case all positive study participants are in home isolation. We have also collected oropharyngeal swabs from 10 healthy uninfected individuals of ICMR-RMRC Bhubaneswar, as a control group during the study period. Cases and controls were collected from the same geographical location viz. the campus of ICMR-RMRC-Bhubaneswar and they were matched for the socioeconomic and ethnic background to avoid lifestyle and food habit related heterogeneity. The original design of the study was to limit the sampling of the cases and the controls to individuals in home isolation, because we wanted to eliminate the confounder of hospital contaminants in the oropharyngeal microbiome. Although we were generally successful in maintaining that design, two individuals had to be admitted to hospital (ID Cov2 and Cov20). We did not want to tamper with the balance of the study design and we were against the idea of throwing away data and hence we continued with those individuals who were hospitalized. Therefore, we have one sample, the Day 15 sample pertaining to the individual (ID Cov2) which was collected 48 h after release from the hospital and the Day 15 and Day 30 samples of individual (ID Cov20), was collected at the hospital.

People predisposed with chronic and infectious lung diseases like tuberculosis (TB), lung-cancer and COPD were excluded from the study. People with clinically established periodontitis disease were also excluded. Healthy controls here are defined as RT-PCR, antibody titre negative for SARS-CoV-2 and devoid of any flu-like symptoms. We also ensured that they did not have antibiotic drug for any reason, in the past one month from the ascertainment into the study and remained clinically negative during their tenure of 30 days. We used sterile cotton swabs for sampling and sampled from the posterior segment of oropharynx through a trained clinician to avoid inter individual variability on sampling and stored the samples in sterile containers adding a lysis buffer and shipped to the laboratory maintaining a 4-degree Celsius temperature. DNA samples were isolated strictly within 4–6 h of collecting the oropharyngeal swabs, with the help of Qiagen biostic bacteremia kit.

Besides our group of Healthy Controls (HC), cases are divided into two groups in post hoc depending on the RT-PCR results on Day 30. The two groups are 1) individuals who were RT-PCR positive at Day30 days (Group A or possible long-term retainers) and 2) individuals who were RT-PCR negative at Day 30 (Group B or individuals who cleared the virus). Healthy controls (HC) have not been under any antibiotic treatment in the last 30 days and were devoid of any known chronic diseases like Diabetes, hypertension, liver diseases, vascular diseases and chronic kidney diseases. As far as the common comorbidities of Covid-19 are concerned, two individuals among the cases (Cov5, Cov19) had a history of Type2 Diabetes Mellitus (T2DM) and one individual (Cov20) had a history of Hypertension (HTN), whereas two individuals (Cov13 and Cov18) had both. The comorbidity status of each individual, i.e the Charlson Comorbidity Index (CCI) is shown in (Fig. 1)^12^. Seven individuals turned out to have severe disease between Day 1 and Day 15 and were on oxygen support (Details in Supplementary Table 1). The remaining thirteen individuals (cases) were mild to moderate as per the World Health Organization criteria for SARS-CoV-2 infection. One individual (ID Cov13), who had pre-established T2DM, HTN and proteinuria, reported water retention in the lungs within the first week of diagnosis. Two of the severe cases were admitted to the hospital and received BiPAP support. Except for these two individuals all cases were in home isolation. All the sampling, except the two hospitalized cases, were done at home. Details of clinical findings that include fever, cough, sore throat and SPO2 status are presented in (Fig. 1) and Supplementary Table 1.

### Sequencing and analysis

Amplicons were generated through 16 s universal primer for variable region 3 and 4 and sequenced on Illumina-NovaSeq 6000 (Supplementary File 1) and analyzed through QIIME (Version 2.0)^45^. Statistical analysis and graphical representations were done using QIIME, Version 2.0^45^ and R, version 4.0.5 (https://www.r-project.org/).

Proportions of OTUs were estimated by normalizing OTU-specific read counts with respect to total read counts. The non-parametric Kolmogorov-Smirnov (KS) method was used to test the equality of distributions. To compare beta diversity among different study groups, we have employed the PERMANOVA test (Supplementary File 2); considering the data points in beta diversity were not independent of each other. Linear discriminant analysis (LDA) was performed to discover the bacterial populations that can differentiate between two groups. To identify the specific OTUs, which were significantly altered in a temporal fashion, we have employed the Microbiome Interpretable Temporal Rule Engine (MITRE) (Supplementary File 3), a supervised machine learning method^13^. Cluster analysis was done by PCA using the Jaccard diversity index.

### Reporting summary

Further information on research design is available in the Nature Research Reporting Summary linked to this article.

## Supplementary information

Supplementary Material

Supplementary table 1

Supplementary table 2

Supplementary table 3

Reporting Summary

## Data Availability

The raw data and Meta data are available in the following link: https://www.ncbi.nlm.nih.gov/bioproject/PRJNA850148.

## Code availability

Raw sequences read were analyzed through default source code of QIIME 2 Module and statistical analysis performed in R. Data specific code is available on request to ab1@nibmg.ac.in, skp1@nibmg.ac.in
