# Supplementary material for: Temporal dynamics of oropharyngeal microbiome among SARS-CoV-2 patients reveals continued dysbiosis even after Viral Clearance
Source: NPJ Biofilms Microbiomes. 2022 Aug 24;8:67. doi: 10.1038/s41522-022-00330-y (PMC9400563; doi:10.1038/s41522-022-00330-y)
Supplement: Supplementary file 1 — Supplementary Material [file 41522_2022_330_MOESM1_ESM.pdf]

Supplementary Figure 1: Schematic Representation of Site Specific Microbiome Population in the Respiratory System

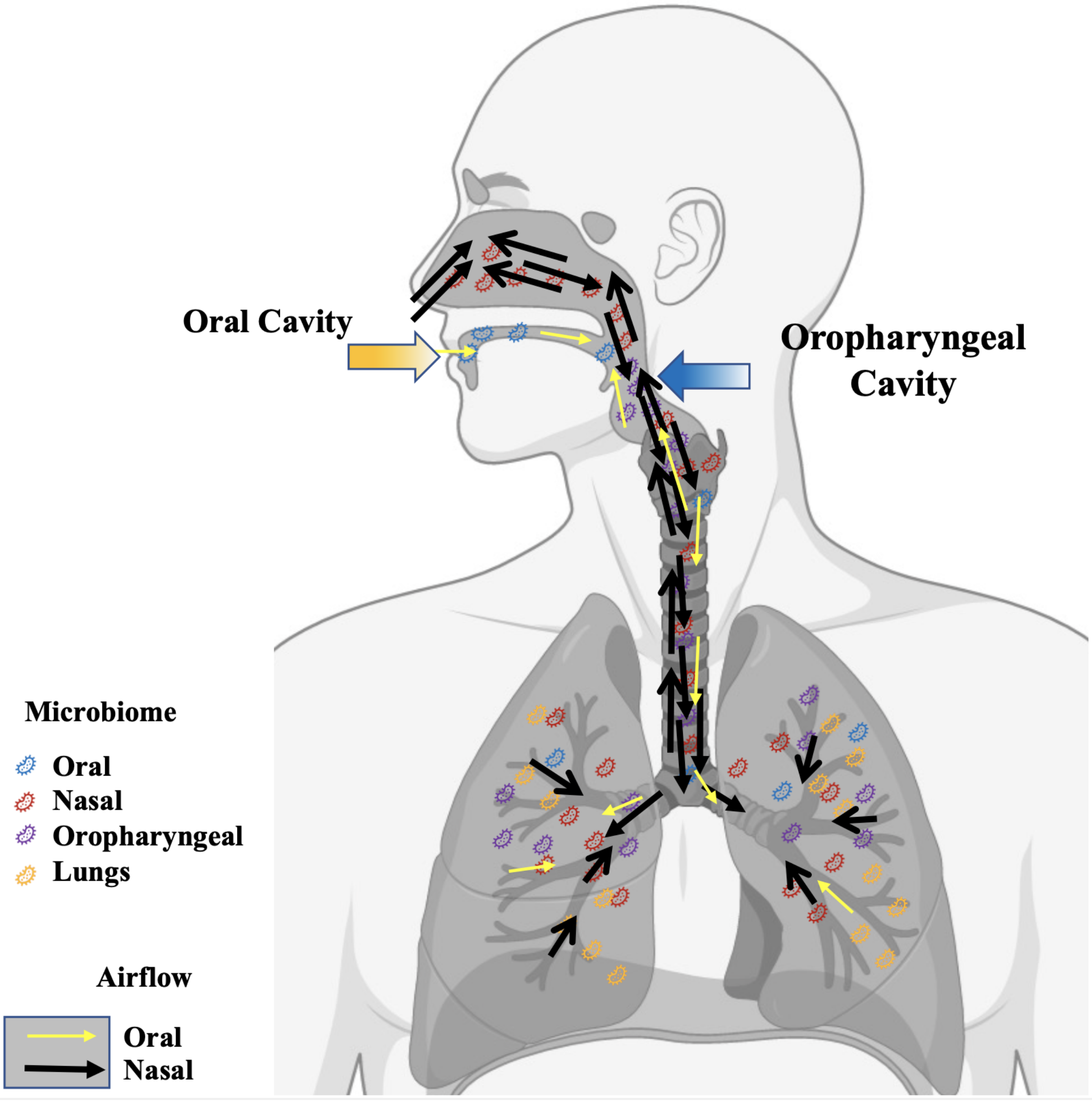

Microbiome population is different between oral and oropharyngeal. Airflow through the nasal cavity via the oropharynx has much higher pressure compared to the oral passage. The same is true when air exhales from the lung through the oropharynx. (created by BioRender)

Supplementary Figure 2

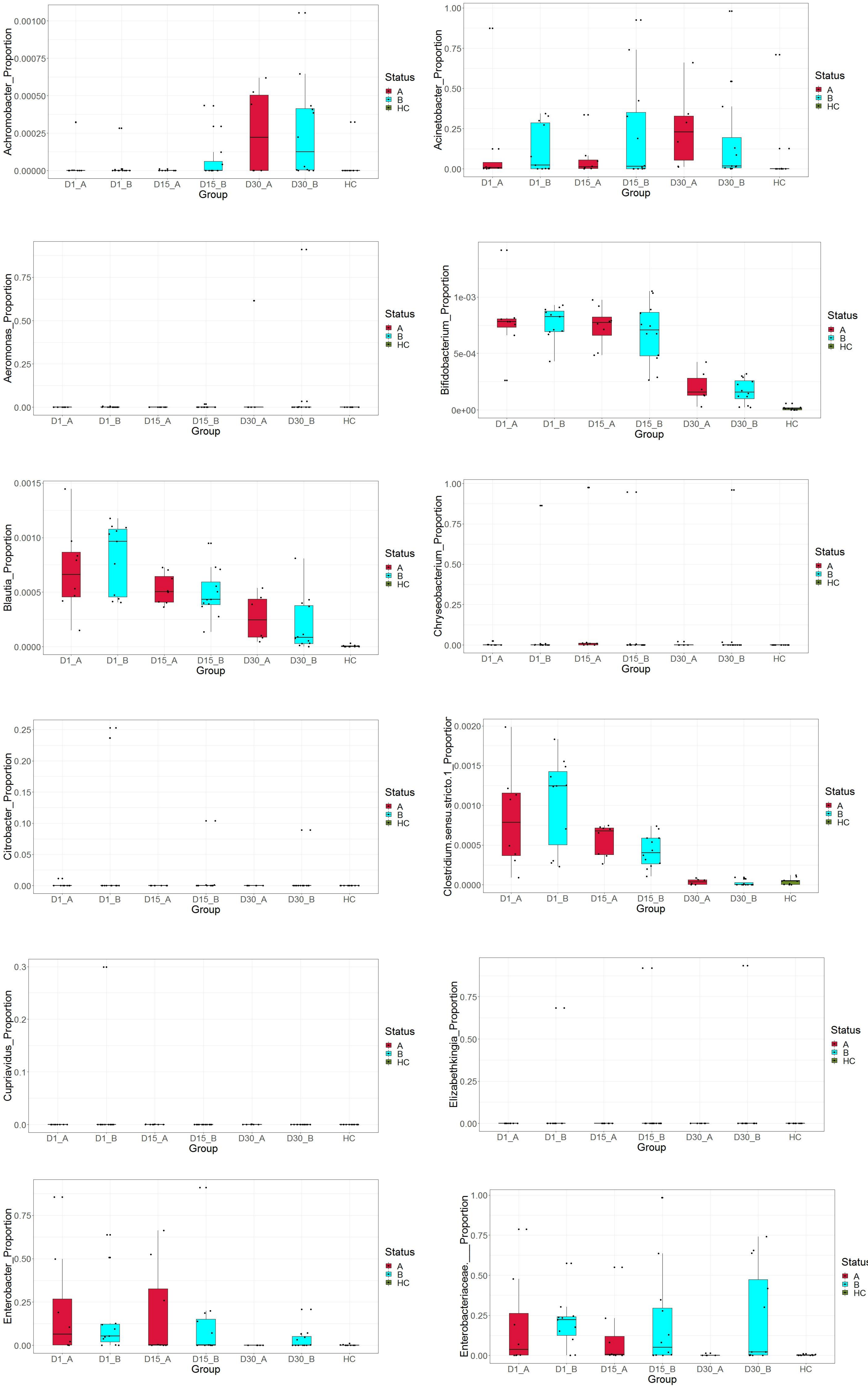

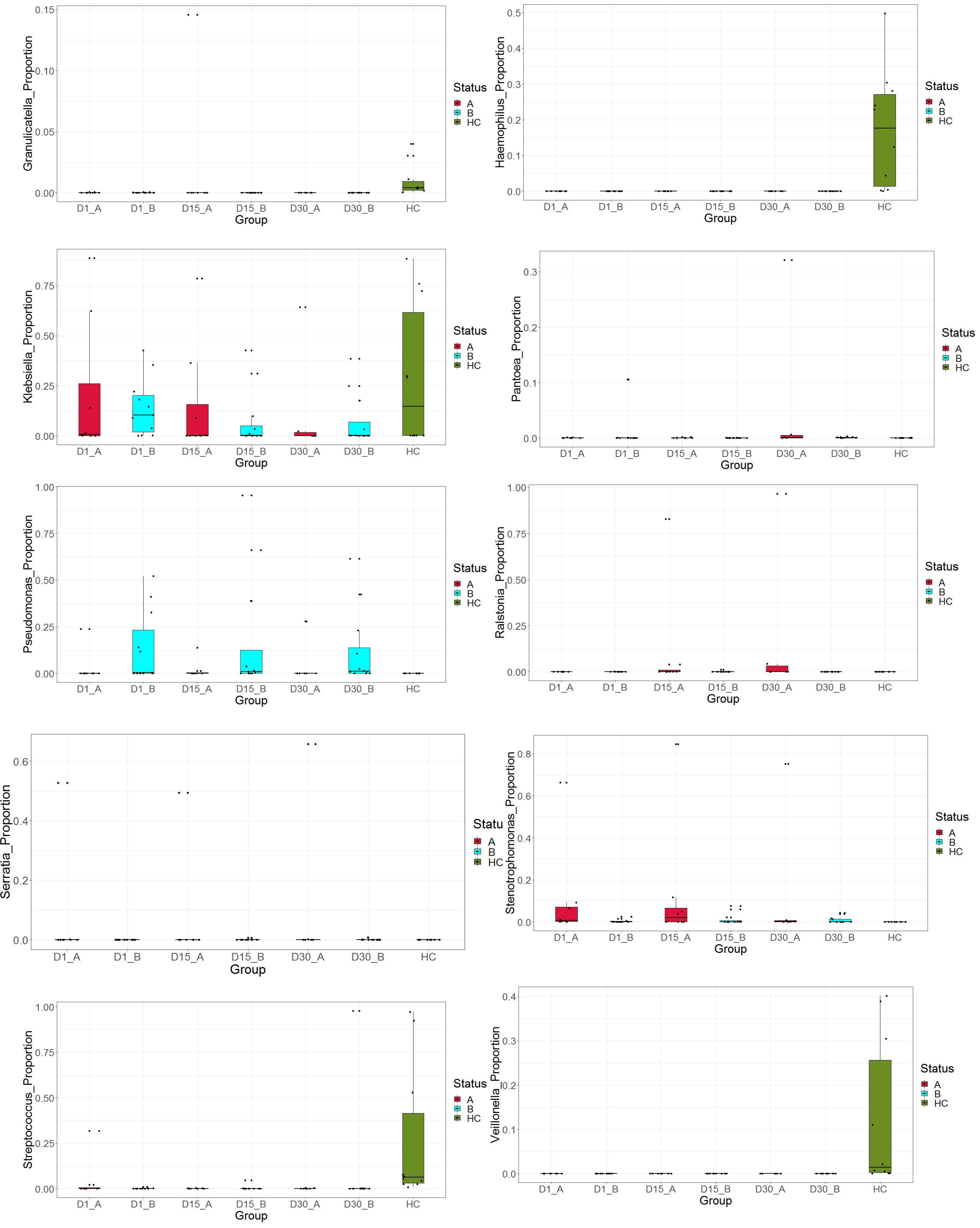

Granulicatella_Proportion
Haemophilus_Proportion
Klebsiella_Proportion
Pantoea_Proportion
Pseudomonas_Proportion
Ralstonia_Proportion
Serratia_Proportion
Stenotrophomonas_Proportion
Streptococcus_Proportion
Veillonella_Proportion
D1_A
D1_B
D15_A
D15_B
D30_A
D30_B
HC
Group
Status
A
B
HC

Note for Supplementary Figure 2: The OTUs which have strong association with cases and higher relative abundance in HC

The study revealed that the genus Veillonella significantly depleted among all cases group in all time point (D1_A vs HC; Pwcox: 0.0007, D1_B vs HC; Pwcox: 0.0005, D15_A vs HC; Pwcox:0.0005, D15_B vs HC; Pwcox:0.0004, D30_A vs HC; Pwcox: 0.006 and D30_B vs HC; Pwcox:0.0005) (Fig.S_A). Similar trend observed for the genus Haemophilus where the abundance significantly high among the HC compared to all cases groups at all time points (Pwcox<0.001). The genus Streptococcus is significantly high among the HC compared to all cases and all time points however the difference is lowest at D1A (D1A vs HC; Pwcox: 0.01) and the difference with rest are almost the same (Pwcox<0.001). The proportion of the genus Blatulia (Pwcox<0.004) and Granulicatella (Pwcox<0.05) significantly elevated among all groups and all time point compared to HC. The genus Chryseobacterium significantly (Pwcox<0.01) elevated among the cases of Group B at Day 1(mean+SD:0.08+0.2) and Day 30 (mean+SD:08+0.2) compared to HC (mean+SD:0.001+0.0001). Further the proportion of the same is elevated at D15 of Group A (mean+SD:0.13+0.34) compared to HC (Pwcox: 0.0009). The genus Clostridium exhibits the significant proportional difference at Day 30 with Day 1 and Day 15 between inter and intra cases groups (P wcox<0.001). The similar trend observed for the genus Bifidobacterium between cases groups (Pwcox<0.005) and with controls (Pwcox<0.001). The genus Blatulia significantly (Pwcox:0.02) decreased at Day 15 (mean+SD:0.0005+0.0002) compared to Day 1 (mean+SD:0.0008+0.0003) in group B. The proportion was altered between Day1 and Day 30 for Group A (Pwcox<0.05). The study further documented that the proportion of the genus at all time points of both the cases group significantly elevated compared to HC (Pwcox<0.004). (Supplementary Figure 2).

Supplementary Figure 3: SDI upon administration of Antibiotics

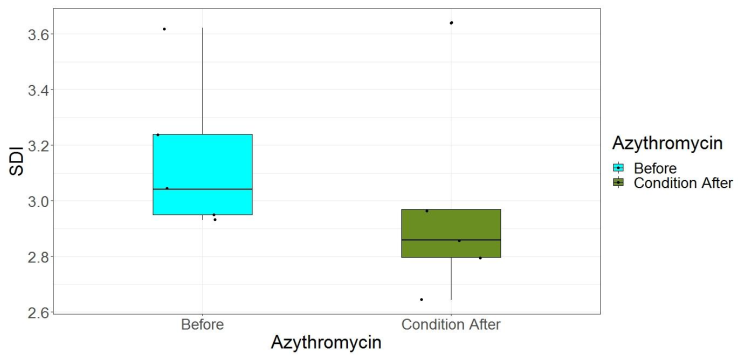

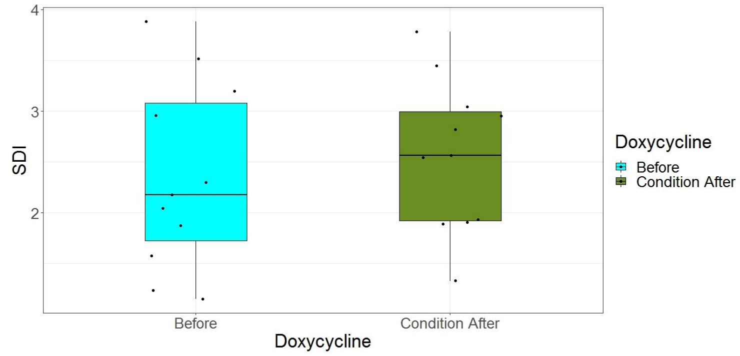

	Azithromycin		Doxycycline	
	Before	After	Before	After
Mean	3.157414127	2.98150761	2.35671686	2.564168534
SD	0.287331927	0.385748951	0.918626453	0.743324918
Min	2.931564958	2.643179968	1.149578054	1.326399555
Max	3.622437692	3.638625063	3.883270265	3.782884302

Note for Supplementary Figure 3

The SDI did not alter significantly upon administration of the both antibiotics that includes Azithromycin and Doxycycline. However an increase trend of SDI documented after administration of Doxycycline among the cases. The trend is reverse for Azithromycin.

Supplementary File 1: Methodology for 16S Amplicon Sequencing (V3V4)

Amplicon Primers:

- The gene-specific sequences used in this protocol target the 16S V3 and V4 region. They are selected from the Klindworth et al. publication (Klindworth A, Pruesse E, Schweer T, Peplles J, Quast C, et al. (2013) Evaluation of general 16S ribosomal RNA gene PCR primers for classical and next-generation sequencing-based diversity studies. Nucleic Acids Res 41(1).) as the most promising bacterial primer pair. Illumina adapter overhang nucleotide sequences are added to the gene-specific sequences. The full length primer sequences, using standard IUPAC nucleotide nomenclature, to follow the protocol targeting this region are:

16S Amplicon PCR Forward Primer = 5'
TCGTCGGCAGCGTCAGATGTGTATAAGAGACAGCCTACGGGNGGCWGCAG
16S Amplicon PCR Reverse Primer = 5'
GTCTCGTGGGCTCGGAGATGTGTATAAGAGACAGGACTACHVGGGTATCTAATCC

- This method can also be utilized to target other regions on the genome (either for 16S with other sets of primer pairs, or non-16S regions throughout the genome; ie any amplicon). The overhang adapter sequence must be added to the locus-specific primer for the region to be targeted (Figure 1). The Illumina overhang adapter sequences to be
- added to locus-specific sequences are:

Forward overhang: 5' TCGTCGGCAGCGTCAGATGTGTATAAGAGACAG-[locus specific sequence]
Reverse overhang: 5' GTCTCGTGGGCTCGGAGATGTGTATAAGAGACAG-[locus specific sequence]

Sequencing details

16s rRNA Gene Sequencing Library Preparation and Sequencing Run:

Genomic DNA was used as the input material for 16s rRNA gene sequencing. Fusion primer was used to amplify V3-V4 region and followed by index PCR using Nextera XT index kit (illumina). Quality of final libraries was checked using high sensitivity D1000 screen tape in 2200 TapeStation (Agilent) and final library quantification was performed in Qubit Fluorometer. Paired end (2 x 250 bp) sequencing of these libraries were performed in Novaseq 6000 (Illumina) SP Flowcell.

Supplementary File 2: Methodology for two way PERMANOVA

Two-way PERMANOVA is a non-parametric method of Multivariate analysis of variance to test the effect of factors Recovery (with 2 levels A, B), Time (with 3 levels D1, D15, D30) on covid Microbiome data. The method works in two steps

1) Calculates the test statistic pseudo F ratio which is a ratio of Mean sum of squares (MS) due to factor and Mean Residual sum of squares.

Sum of squares are calculated from Distances Matrix obtained using "Bray-Curtis" distance measure

2) Calculation of P-value using Permutation test, P-value for each factor and their interaction is considered to be significant if less than 0.05 after multiple testing correction (FDR).

Reference:

Anderson MJ. A new method for non-parametric multivariate analysis of variance. Austral ecology. 2001 Feb;26(1):32-46.

Supplementary File 3: Methodology for MITRE

Microbiome Interpretable Temporal Rule Engine (MITRE)

MITRE, a Bayesian supervised machine learning classifier uses longitudinal microbiome data with the labelled binary host status to identify the essential set of microbiome and relevant time windows to explain the host status.

Inputs

We provide longitudinal microbiome data as an input to the MITRE model. Let us assume we have measurements $\left\{x_{ij}\left(t_{j1}\right),\ x_{ij}\left(t_{j2}\right), x_{ij}\left(t_{j3}\right), ...\right\}$ of the abundance of the OTUsi$\in\left\{1, 2, 3,\ ....,\ N_{OTUs}\right\}$of the samples of the subjects j$\in\left\{1, 2, 3,\ ....,\ N_{subjects}\right\}$ at time points $\left\{t_{j1}, t_{j2}, t_{j3}, ...\right\}$from the jth subject, number of samples varies across the subjects and belongs to the time window [0,$T_{experiment}$], length of the experiment$T_{experiment}$is 12 months.For each subject we have a binary host status $y_j$which is independent of time.

Generation of Detectors from Data

user specified parameter to be used for generating detectors
$t_{min} = 1, t_{max} = 12, N_w = 4, N_\theta = no\ maximum.$

where,
t_{min}= starting time of the experiment, Rules may apply to time windows greater than t_{min}
t_{max} = length of the experiment, Rules may apply to time windows this long or shorter.
N_w = Number of intervals into which study duration to be divided.
N = maximum number of threshold values allowed for any feature

If $N_\theta = no\ maximum$ then $N_\theta = N_{subjects} - 1$

A pool of detectors Dgenerated using input data and user specified parameters mentioned above as follows:

1. Divide the duration of experiment $T_{experiment}$into N_w equal intervals such that at least one sample collected for each subject in each time widow.
2. Within each time window for feature i, calculate average abundance of the feature in each subject j and sort those values, obtaining $f_1 f_2 .... f_{N_{subjects}}$.

   for $m = 1, 2, ...\ N_{subjects} - 1$, let $_m = \frac{f_m + f_{m+1}}{2}$ and add the detector "between time t_a and t_b , the average abundance of the feature i is [above/below] $_m$"to the pool of detectors$D$.

3. Repeat the process for each time window during which at least two samples collected for every subject, calculating the slope of each feature i in each subject j and add detector

“between time t_a and t_b , the slope of the abundance of feature i is [above/below] $_m$” to the pool of detector D.

Mathematical basis of MITRE model

MITRE predicts host status y_j from set of rules using a Bayesian Logistic regression model defined as follows:

$$y_j \sim Bernoulli\left(p_j\right)$$
$$p_j = \frac{1}{1+e^{-\psi_j}}$$
$$\psi = A(R, x)\beta$$
$$\beta|R \sim N(0, \sigma_b^2 I)$$
$$R \sim \pi(R, x)$$

R is a set of Rules which are collection of detectors from the population of detectors generated above:

$$R = \left(\rho_1, \rho_2, \rho_3, ..., \rho_m\right)$$

Rule ρ_k is a defined as

$$\rho_k = (r_{k1}, r_{k2}, r_{k3}, ..., r_{kn_k} \; ; k = 1, 2, 3, ..., m$$

The transformed matrix $A(R, x)$ of covariates is obtained from R as

$$A(R, x) = \left(v_0, v_1, v_2, ..., v_m\right)$$

Where $v_0 = (1, 1, 1........., 1)^T$

$$v_k = (v_{1k}, v_{2k}, v_{3k}, ..., v_{N_{subject}k})^T; k = 1, 2, ..., m$$

$$v_{jk} = \{1\; 0 \; \frac{if\; k^{th}\; rule\; is\; true\; for\; j^{th}\; subject}{if\; k^{th}\; rule\; is\; false\; for\; j^{th}\; subject}$$

If rule list R is empty $A(R, x) = v_0$.

Prior probability distribution on rule sets

The prior probability distribution is described by following generative process:

- With probability $\Theta_0 \sim Beta(a_\theta, b_\theta)$, Rule set has length 0; otherwise, it has some nonzero length.

$a_\theta = 0.5,\ b_\theta = 0.5$, for a 50% prior probability that the list is empty.

- If the Rule set is non empty generate using according to following procedure:

    1. Set Rule set length to m, where excess length $m - 1$ is drawn from a truncated negative binomial distribution with parameters α_m and β_m and maximum value $m_{max} - 1$.
    2. For i^{th} rule draw excess length $n_i - 1$ from a truncated negative binomial distribution with parameters α_n and β_n and maximum value $n_{max} - 1$.
    3. For i^{th}rule draw the vector $z^{(i)} \sim Multinomial(n_i, p)$
       Where $p = (p_1, p_2, \ldots\ldots, p_{n_D})$

We can write the above generative process as

$$\pi(R) = \{\Theta_0\ \ m = 0\ \ (1 - \Theta_0)P_m(m - 1) \prod_{i=1}^{m} (P_n(n_i - 1)M_i p(r_{i1}) \ldots\ldots p(r_{in_i}))\qquad m > 0$$

M_i is the multinomial coefficient, $M_i = \frac{n_i!}{\prod_{c=0}^{n_D} z_c^{(i)}!}$

P_m(Respectively P_n) is probability mass function of truncated negative binomial distribution with minimum value 0 maximum value $m_{max} - 1$ ($n_{max} - 1$), andparameters α_m, β_m(α_n, β_n).

Default parameters are

$$\alpha_m = 0.5, \beta_m = 2.0, \alpha_n = 2.0, \beta_n = 4.0, m_{max} = 10,\ n_{max} = 10$$

The parameters chosen reflects the belief that a rule set containing one rule with twodetectors are generally more straightforward to interpret than a rule set containing tworules each with one detector.

Also, the prior probability of R independent of the particular detector itcontains is defined as

$$\pi_s(R) = \{\Theta_0\, m = 0\ \ (1 - \Theta_0)P_m(m - 1) \prod_{i=1}^{m} P_n(n_i - 1)\qquad m > 0$$

Prior distribution over detectors

For each detector $r^{(k)}$ in D, we assign a prior probability

$$p\left(r^{(k)}\right) \propto G\left(r^{(k)}\right) w\left(r^{(k)}\right)$$

Each element of Where Phylogeny prior $G\left(r^{(k)}\right) \sim N(\log log\ L_i;\ \mu_L, \sigma_L^2)$

$$\mu_L \sim N\left(\Lambda_L, 50\Delta_L^2\right)$$

$$\sigma_L \sim Uniform\left(0, 25\Delta_L\right)$$

Where, Λ_L is median of logarithms of weights L_i on the variables

$\Delta_L = L_h - L_l$

L_l, L_h is 2.5th and 97.5th percentile of the logarithms of weights L_i on the variables.

When we don't want to take phylogeny information into account, same weight is assigned to all the variables and assigning Δ_L a very small finite minimum value.

And prior on time window length $w\left(r^{(k)}\right) \sim Beta\left(\frac{W_k}{\left(\left(1+\epsilon_w\right)T_{experiment}\right.}; \alpha_w, \beta_w\right)$

Where $\epsilon_w = 0.01$,

$$\alpha_w = c_w f_w$$

$$\beta_w = c_w(1 - f_w)$$

f_w is prior mean window length distributed as $Uniform(0, 1)$.

c_w is distributed as $Exponential(0.2)$

Inference Algorithm

Now we have longitudinal microbiome data and prior distribution on parameters Rule set R and regression coefficients β, we will use Markov Chain Monte Carlo (MCMC) algorithm to infer the posterior distribution of Rule set R and regression coefficient βgiven data.

Initialization

For initial state of Markov chain R have been set to a rule set with one rule containing single detector, chosen from detector population to minimize the Hamming distance between the detectors vector of true values and true outcome variable. Each element of ωset to 1, each element of βto 0.

The MCMC algorithm with the following iterated steps has been used

1. Update the regression coefficient β, drawing new values from their conditional distribution using a data augmentation technique.

   To infer β, data augmentation approach has been used. Let ω_j be the auxiliary variable follows Polya-Gamma distribution.

   $\omega_j \sim PG(1, a_j^T\beta)$; $j = 1, 2, \ldots\ldots, N_{subjects}$
   $a_j = (A_{j1}, A_{j2}, \ldots\ldots, A_{j(m+1)})$ is j^{th} row of $A(R, x)$.

   The Augmented Posterior distribution is given by

$$\pi(y) = \frac{1}{c(y)}\pi(\beta)f(\beta)\prod_{j=1}^{N_{subjects}} P(y_j|\beta)$$

   Which gives the same posterior density $\pi(y)$as the original logistic regression After marginalizing out ω.

   The posterior distribution of $\pi(y, \omega)$is a multivariate normal distribution defined as

$$\beta|y, \omega \sim N(m_\omega, V_\omega)$$

where,

$$V_\omega = \left(A^T\Omega A + \frac{1}{\sigma_\beta^2}I\right)^{-1}$$

$$m_\omega = V_\omega A^T\kappa$$

$$\Omega = diag(\omega)$$

$$\kappa = \left(y_1 - \frac{1}{2}, y_2 - \frac{1}{2}, \ldots.., y_{N_{subjects}} - \frac{1}{2}\right)$$

$$z = \left(\frac{k_1}{\omega_1}, \frac{k_2}{\omega_2}, \ldots.., \frac{k_{N_{subjects}}}{\omega_{N_{subjects}}}\right)$$

2. Update the Rule set R via Metropolis-Hasting step

   The Posterior distribution of R by marginalizing out regression coefficient βis

$$\pi(\omega, y) \propto \pi(R) exp\left\{-\frac{1}{2} z^T (\Omega^{-1} + \sigma_\beta^2 A A^T) z\right\}$$

When a new R is chosen, a new value of βis drawn from the new conditional distribution of βgiven $A(R)$ and ω**.**

The algorithm proposes following modifications to structure of R

- Replacing one detector in Rule set R with another detector from D**.**

  We choose a detector r_{ij} in R at random and obtain a new rule list R' by deleting r_{ij} from R. We form a set of rule sets $\Delta = \{R'_1, R'_2, \ldots\ldots., R'_{n_D}\}$.

  Set of rule sets Δ resulting from every possible replacement of r_{ij} with any element in D.

  Finally, a new state from Δ is chosen with probability proportional to

  $\{(z_1^{'(i)} + 1)\pi(R'_1 | \omega, y), \ldots\ldots., (z_{n_D}^{'(i)} + 1)\pi(R'_{n_D} | \omega, y)\}$

  $z_k^{'(i)}$, number of copies of detector $r^{(k)}$ in rule i

- Adding a detector to the rule set

  Addition of a detector can be done in two ways. With probability $(m + 1)/(2m + 1)$ insertion of a new rule is proposed and with probability $m/(2m + 1)$ insertion of detector into existing rule is proposed.

  Addition of a new rule

  Suppose the we have proposed addition of new rule at i^{th} position in the rule Set. The set of possible rule sets by adding detectors from Das a new Rule is Is defined as

  $$S^* = \{R'_1, R'_2, \ldots\ldots., R'_{n_D}\}.$$

  If the transition into S^* is accepted we choose a particular R' from S^* according to posterior distribution of rule set $\pi(\omega, y)$restricted to S^*.

  Addition to an existing rule

  if it is proposed to add the detector to an existing rule i, the set of possible rule set by adding the detectors from D in Rule i is defined as

  $$S^* = \{R'_1, R'_2, \ldots\ldots., R'_{n_D}\}$$

If the transition into S^* is accepted we choose a particular R' from S^* according to posterior distribution of rule set $\pi(\omega, y)$restricted to S^*.

- Removal of a detector

  Removal step is a reverse of addition step described above i.e.; we consider it as a transition back to shorter rule set from the space of possible rule set obtained by addition of detector at a particular position in that shorter rule set. If we are removing a detector from length 1 rule in R then rule itself will be removed otherwise length of a rule will be decreased by 1. Let

$$R^- = \{\left(\rho_1, \rho_2, \rho_3, \ldots, \rho_{i-1}, \rho_{i+1} \ldots, \rho_m\right), \quad n = 1 \left(\rho_1, \rho_2, \rho_3, \ldots, (r_{i1}, \ldots r_{i(j-1)}, r_{i(j+1)}, \ldots, r_{in_i}) \ldots, \rho_m\right), \qquad n_i > 1$$

  The set of possible rules S^* formed by adding detectors from D to the relevant position in R^-. If the transition from S^* to R^- is accepted we remove a detector.

- Detector moves

  If the rule list is empty or contains only one detector, we do nothing. Otherwise, a detector r_{ij} in R chosen at Random and assigned to every rule in R or to a new rule of length 1.

3. Update the hyperparameters controlling the prior distribution $\pi(R)$

updated values drawn for Θ_0 from $Beta(a_\theta + 1, b_\theta)$ if the rule list is empty or $Beta(a_\theta, b_\theta + 1)$ if the rule list is non empty.

The phylogenetic prior parameters $\mu_L$and $\sigma_L$are updated with proposed values

$$\sigma_L' = \|\sigma_L + \delta_\sigma\|, \mu_L' = \|\mu_L + \delta_\mu\|$$

Where,

$$\delta_\mu \sim N(0, \frac{\Delta_L^2}{4})$$

$$\delta_\sigma \sim N(0, \frac{\Delta_L}{2})$$

For the time window prior parameters f_w and c_w proposed values are

$$f_w' = \|\left(f_w + \delta_f + 1\right) mod\, 2 \; - 1\| and\; c_w' = \|c_w + \delta_w\|$$

where,

$$\delta_f \sim N(0, 0.04)$$

$$\delta_w \sim N(0, 0.04 * 25)$$

Point Estimate of the posterior samples

After MCMC algorithm with steps mentioned above iterated a large number of times. The rule set R^* with associated coefficient β^* is chosen to be a high likelihood rule set that is representative of the posterior samples that is, R^* contains the posterior mode d^* of the total number of detectors in R.

Bayes Factor

The prior probability that Ruleset R is empty is $a_\theta/(a_\theta + b_\theta)$, if fraction of posterior rule set samples which are empty is f, the bayes factor for the empty rule set is defined as

$$B \ = \ posterior\ odds/prior\ odds \ = \ \frac{f}{1-f}\frac{b_\theta}{a_\theta}$$

Supplementary Table 1: **Detailed Demographic and Clinical Characteristics of Cases and Healthy Controls**

Sl No	ID	Age	Sex	RT PCR Positive	Clinical Status	Clinical Symptoms	Fever	Cough	Low SpO2	External oxygen supplement	Comorbidity	Charlson Comorbidity Index	Diabetes	Hypertension	Antibiotic usage	Duration of antibiotic therapy
	**Cov11_D1**			Yes	Mild	Cough, Cold, Fever	Yes	Yes	No	None					No	NA
1	**Cov11_D15**	59	M	Yes	Asymptomatic	Asymptomatic	No	No	No	None	No comorbidity	1	No	No	Doxycycline 100mg	5 days
	**Cov11_D30**			Yes	Asymptomatic	Asymptomatic	No	No	No	None					No	NA
															No	NA
	**Cov12_D1**			Yes	Mild	Cough, Cold, Fever	Yes	Yes	No	None					No	NA
2	**Cov12_D15**	21	M	Yes	Asymptomatic	Asymptomatic	No	No	No	None	No comorbidity	0	No	No	Doxycycline 100mg	5 days
	**Cov12_D30**			Yes	Asymptomatic	Asymptomatic	No	No	No	None					No	NA
															No	NA
	**Cov13_D1**			Yes	Severe	Cough, Cold, Fever, Sore throat, SPO2<90%	Yes	Yes	Yes	Nasal Cannula					No	NA
3	**Cov13_D15**	64	M	Yes	Moderate	Cough, Cold,	No	Yes	No	None	Diabetes, Hypertension, One kidney damaged, water retention in lung, Cholesterol high	7	Yes	Yes	No	NA
	**Cov13_D30**			Yes	Mild	Cough	No	Yes	No	None					No	NA
															No	NA
	**Cov14_D1**			Yes	Severe	Cough, Cold, Fever, SPO2<90%	Yes	Yes	Yes	Nasal Canula					No	NA
4	**Cov14_D15**	55	F	Yes	Moderate	Cough, Cold, Fever	Yes	Yes	No	None	No comorbidity	1	No	No	No	NA
	**Cov14_D30**			Yes	Mild	Cough	No	Yes	No	None					No	NA
															No	NA

Sl No	ID	Age	Sex	RT PCR Positive	Clinical Status	Clinical Symptoms	Fever	Cough	Low SpO2	External oxygen supplement	Comorbidity	Charlson Comorbidity Index	Diabetes	Hypertension	Antibiotic usage	Duration of antibiotic therapy
	**Cov15_D1**			Yes	Mild	Cough, Fever	Yes	Yes	No	None					No	NA
5	**Cov15_D15**	28	F	Yes	Mild	Dry Cough	No	Yes	No	None	No comorbidity	0	No	No	No	NA
	**Cov15_D30**			No	Mild	Dry Cough	No	Yes	No	None					No	NA
															No	NA
	**Cov16_D1**			Yes	Mild	Cough, Fever	Yes	Yes	No	None					No	NA
6	**Cov16_D15**	24	F	Yes	Moderate	Dry Cough	No	Yes	No	None	No comorbidity	0	No	No	No	NA
	**Cov16_D30**			Yes	Mild	Dry Cough	No	Yes	No	None					No	NA
															No	NA
	**Cov17_D1**			Yes	Mild	Cough, Fever	Yes	Yes	No	None					No	NA
7	**Cov17_D15**	21	F	Yes	Mild	Dry Cough	No	Yes	No	None	No comorbidity	0	No	No	No	NA
	**Cov17_D30**			No	Mild	Cough	No	Yes	No	None					No	NA
															No	NA
	**Cov18_D1**			Yes	Moderate	Cold, Fever	Yes	No	No	None					No	NA
8	**Cov18_D15**	62	M	Yes	Mild	Cold	No	No	No	None	Diabetes, Hypertension	4	Yes	Yes	Azithromycin 500mg	5 days
	**Cov18_D30**			No	Asymptomatic	Asymptomatic	No	No	No	None					No	NA
															No	NA

Sl No	ID	Age	Sex	RT PCR Positive	Clinical Status	Clinical Symptoms	Fever	Cough	Low SpO2	External oxygen supplement	Comorbidity	Charlson Comorbidity Index	Diabetes	Hypertension	Antibiotic usage	Duration of antibiotic therapy
	**Cov19_D1**			Yes	Severe	Cold, Fever, Sore throat , Cough SPO2<90%	Yes	Yes	Yes	Nasal Canula					Doxycycline 100mg	5 days
9	**Cov19_D15**	60	F	Yes	Moderate	Cold	No	No	No	None	Diabetes	3	Yes	No	Azithromycin 500mg	3 days after completion of Doxycycline dose
	**Cov19_D30**			Yes	Asymptomatic	Asymptomatic	No	No	No	None					No	NA
															No	NA
	**Cov7_D1**			Yes	Moderate	Cough, Cold, Fever	No	Yes	No	None					No	NA
10	**Cov7_D15**	30	F	Yes	Asymptomatic	Asymptomatic	No	No	No	None	No comorbidity	0	No	No	No	NA
	**Cov7_D30**			No	Asymptomatic	Asymptomatic	No	No	No	None					No	NA
															No	NA
	**Cov8_D1**			Yes	Moderate	Cough, Cold, Fever	No	Yes	No	None					No	NA
11	**Cov8_D15**	54	F	Yes	Asymptomatic	Asymptomatic	No	No	No	None	No comorbidity	1	No	No	Doxycycline 100mg	5 days
	**Cov8_D30**			Yes	Asymptomatic	Asymptomatic	No	No	No	None					No	NA
															No	NA
	**Cov4_D1**			Yes	Mild	Cough, Cold, Fever,Loss of smell and taste, weakness	Yes	Yes	No	None					No	NA
12	**Cov4_D15**	31	F	Yes	Mild	Dry Cough	No	Yes	No	None	No Comorbidity	0	No	No	No	NA
	**Cov4_D30**			No	Asymptomatic	Asymptomatic	No	No	No	None					No	NA

Sl No	ID	Age	Sex	RT PCR Positive	Clinical Status	Clinical Symptoms	Fever	Cough	Low SpO2	External oxygen supplement	Comorbidity	Charlson Comorbidity Index	Diabetes	Hypertension	Antibiotic usage	Duration of antibiotic therapy
															No	NA
	**Cov2_D1**			Yes	Severe	Cough, Cold, Fever, Headache, Sore throat, Loss of smell and taste, weakness	Yes	Yes	Yes	Nasal Canula					No	NA
13	**Cov2_D15**	28	F	Yes	Moderate	Dry Cough, Cold, Loss of smell and taste, weakness	No	Yes	No	BiPAP	No Comorbidity	0	No	No	Doxycycline 100mg	5 days
	**Cov2_D30**			Yes	Mild	Dry Cough	No	Yes	No	None					No	NA
															No	NA
	**Cov20_D1**			Yes	Severe	Cough, Cold, Fever, Sore throat, Breathelessness, SPO2<90%	Yes	Yes	Yes	Nasal Canula					Doxycycline 100mg	5 days
14	**Cov20_D15**	59	M	Yes	Severe	Cough, Cold, SPO2<90%	No	Yes	Yes	BiPAP	Hypertension	2	No	Yes	Azithromycin 500mg	3 days after completion of Doxycycline [illegible]
	**Cov20_D30**			Yes	Moderate	Cough, Cold	No	Yes	No	BiPAP					No	NA
															No	NA
	**Cov3_D1**			Yes	Mild	Cough, Cold, Fever, Loss of smell and taste	Yes	Yes	No	None					No	NA
15	**Cov3_D15**	30	M	Yes	Asymptomatic	Asymptomatic	No	No	No	None	No Comorbidity	0	No	No	No	NA
	**Cov3_D30**			No	Asymptomatic	Asymptomatic	No	No	No	None					No	NA
															No	NA
	**Cov5_D1**			Yes	Moderate	Light Cough, Positive during 1st Wave too.	No	Yes	No	None					No	NA
16	**Cov5_D15**	34	M	Yes	Asymptomatic	Asymptomatic	No	No	No	None	Diabetes	1	Yes	No	Doxycycline 100mg	5 days

Sl No	ID	Age	Sex	RT PCR Positive	Clinical Status	Clinical Symptoms	Fever	Cough	Low SpO2	External oxygen supplement	Comorbidity	Charlson Comorbidity Index	Diabetes	Hypertension	Antibiotic usage	Duration of antibiotic therapy
	**Cov5_D30**			Yes	Asymptomatic	Asymptomatic	No	No	No	None					No	NA
															No	NA
	**Cov6_D1**			Yes	Mild	Cough, Cold, Fever	Yes	Yes	No	None					No	NA
17	**Cov6_D15**	22	M	Yes	Asymptomatic	Asymptomatic	No	No	No	None	No comorbidity	0	No	No	No	NA
	**Cov6_D30**			No	Asymptomatic	Asymptomatic	No	No	No	None					No	NA
															No	NA
	**Cov1_D1**			Yes	Severe	Cough, Cold, Fever, Headache, Sore throat, Loss of smell and taste, [illegible]	Yes	Yes	Yes	Nasal Canula					No	NA
18	**Cov1_D15**	30	M	Yes	Moderate	Dry Cough, Cold, Loss of smell and taste, weakness	No	Yes	No	None	No Comorbidity	0	No	No	Doxycycline 100mg	5 days
	**Cov1_D30**			Yes	Mild	Dry Cough, weakness	No	Yes	No	None					No	NA
															No	NA
	**Cov10_D1**			Yes	Severe	Cough, Cold, Fever, SPO2<90%	Yes	Yes	Yes	Nasal Canula					No	NA
19	**Cov10_D15**	55	M	Yes	Moderate	Cough, Cold	No	Yes	No	None	Prediabetic	2	Yes	No	Doxycycline 100mg	5 days
	**Cov10_D30**			Yes	Asymptomatic	Asymptomatic	No	No	No	None					No	NA
															No	NA
	**Cov9_D1**			Yes	Mild	Cough, Cold, Fever	Yes	Yes	No	None					No	NA

Sl No	ID	Age	Sex	RT PCR Positive	Clinical Status	Clinical Symptoms	Fever	Cough	Low SpO2	External oxygen supplement	Comorbidity	Charlson Comorbidity Index	Diabetes	Hypertension	Antibiotic usage	Duration of antibiotic therapy
20	**Cov9_D15**	24	F	Yes	Asymptomatic	Asymptomatic	No	No	No	None	No comorbidity	0	No	No	Doxycycline 100mg	5 days
	**Cov9_D30**			No	Asymptomatic	Asymptomatic	No	No	No	None					No	NA
															No	NA
21	**HC1**	45	M	No	Healthy	NA	No	No	No	None	NA	0	No	No	No	No
22	**HC2**	53	M	No	Healthy	NA	No	No	No	None	NA	0	No	No	No	No
23	**HC3**	23	M	No	Healthy	NA	No	No	No	None	NA	0	No	No	No	No
24	**HC4**	29	F	No	Healthy	NA	No	No	No	None	NA	0	No	No	No	No
25	**HC5**	37	F	No	Healthy	NA	No	No	No	None	NA	0	No	No	No	No
26	**HC6**	29	F	No	Healthy	NA	No	No	No	None	NA	0	No	No	No	No
27	**HC7**	51	M	No	Healthy	NA	No	No	No	None	NA	0	No	No	No	No
28	**HC8**	55	M	No	Healthy	NA	No	No	No	None	NA	0	No	No	No	No
29	**HC9**	29	M	No	Healthy	NA	No	No	No	None	NA	0	No	No	No	No
30	**HC10**	44	F	No	Healthy	NA	No	No	No	None	NA	0	No	No	No	No

Supplementary Table 2: Genus specific OTUs of both cases and controls in all the time points (Day 1, Day 15 and Day 30)

Index	ID	Age	Sex	Day	Ct-value	[illegible]
[illegible]	[illegible]	[illegible]	[illegible]	[illegible]	[illegible]	[illegible]

Supplementary Table 3: List of OTUs present among Healthy Controls and Cases in different time points

OTUs present among HC	OTUs present among Cases_Day1	OTUs present among Cases_Day15	OTUs present among Cases_Day30	OTUs present among only Cases_Day30_A	OTUs present among only Cases_Day30_B
D_0__Bacteria.D_1__Actinobacteria.D_2__Actinobacteria.D_3__Bifidobacteriales.D_4__Bifidobacteriaceae.D_5__Scardovia	D_0__Bacteria.D_1__Actinobacteria.D_2__Actinobacteria.D_3__Actinomycetales.D_4__Actinomycetaceae.D_5__Actinomyces	D_0__Archaea.D_1__Euryarchaeota.D_2__Methanobacteria.D_3__Methanobacteriales.D_4__Methanobacteriaceae.D_5__Methanobrevibacter	D_0__Bacteria.D_1__Acidobacteria.D_2__Acidobacteriia.D_3__Solibacterales.D_4__Solibacteraceae..Subgroup.3..D_5__Bryobacter	D_0__Bacteria.D_1__Actinobacteria.D_2__Actinobacteria.D_3__Corynebacteriales.D_4__Corynebacteriaceae.__	D_0__Bacteria.D_1__Actinobacteria.D_2__Actinobacteria.D_3__Actinomycetales.D_4__Actinomycetaceae.D_5__Actinomyces
D_0__Bacteria.D_1__Actinobacteria.D_2__Actinobacteria.D_3__Corynebacteriales.D_4__Corynebacteriaceae.D_5__Corynebacterium	D_0__Bacteria.D_1__Actinobacteria.D_2__Actinobacteria.D_3__Actinomycetales.D_4__Actinomycetaceae.D_5__Arcanobacterium	D_0__Bacteria.D_1__Acidobacteria.D_2__Acidobacteriia.D_3__Solibacterales.D_4__Solibacteraceae..Subgroup.3..D_5__Bryobacter	D_0__Bacteria.D_1__Actinobacteria.D_2__Actinobacteria.D_3__Actinomycetales.D_4__Actinomycetaceae.D_5__Actinomyces	D_0__Bacteria.D_1__Actinobacteria.D_2__Actinobacteria.D_3__Corynebacteriales.D_4__Nocardiaceae.D_5__Gordonia	D_0__Bacteria.D_1__Actinobacteria.D_2__Actinobacteria.D_3__Actinomycetales.D_4__Actinomycetaceae.D_5__Mobiluncus
D_0__Bacteria.D_1__Actinobacteria.D_2__Actinobacteria.D_3__Micrococcales.D_4__Dermabacteraceae.D_5__Dermabacter	D_0__Bacteria.D_1__Actinobacteria.D_2__Actinobacteria.D_3__Actinomycetales.D_4__Actinomycetaceae.D_5__Mobiluncus	D_0__Bacteria.D_1__Actinobacteria.D_2__Actinobacteria.D_3__Actinomycetales.D_4__Actinomycetaceae.D_5__Actinomyces	D_0__Bacteria.D_1__Actinobacteria.D_2__Actinobacteria.D_3__Actinomycetales.D_4__Actinomycetaceae.D_5__Mobiluncus	D_0__Bacteria.D_1__Actinobacteria.D_2__Actinobacteria.D_3__Micrococcales.D_4__Cellulomonadaceae.__	D_0__Bacteria.D_1__Actinobacteria.D_2__Actinobacteria.D_3__Actinomycetales.D_4__Actinomycetaceae.D_5__Varibaculum
D_0__Bacteria.D_1__Actinobacteria.D_2__Actinobacteria.D_3__Micrococcales.D_4__Micrococcaceae.__	D_0__Bacteria.D_1__Actinobacteria.D_2__Actinobacteria.D_3__Bifidobacteriales.D_4__Bifidobacteriaceae.D_5__Bifidobacterium	D_0__Bacteria.D_1__Actinobacteria.D_2__Actinobacteria.D_3__Actinomycetales.D_4__Actinomycetaceae.D_5__Mobiluncus	D_0__Bacteria.D_1__Actinobacteria.D_2__Actinobacteria.D_3__Actinomycetales.D_4__Actinomycetaceae.D_5__Varibaculum	D_0__Bacteria.D_1__Actinobacteria.D_2__Actinobacteria.D_3__Micrococcales.D_4__Dermabacteraceae.D_5__Dermabacter	D_0__Bacteria.D_1__Actinobacteria.D_2__Actinobacteria.D_3__Corynebacteriales.D_4__Corynebacteriaceae.D_5__Corynebacterium
D_0__Bacteria.D_1__Actinobacteria.D_2__Actinobacteria.D_3__Propionibacteriales.D_4__Nocardioidaceae.D_5__Marmoricola	D_0__Bacteria.D_1__Actinobacteria.D_2__Actinobacteria.D_3__Bifidobacteriales.D_4__Bifidobacteriaceae.D_5__Gardnerella	D_0__Bacteria.D_1__Actinobacteria.D_2__Actinobacteria.D_3__Actinomycetales.D_4__Actinomycetaceae.D_5__Varibaculum	D_0__Bacteria.D_1__Actinobacteria.D_2__Actinobacteria.D_3__Bifidobacteriales.D_4__Bifidobacteriaceae.D_5__Bifidobacterium	D_0__Bacteria.D_1__Actinobacteria.D_2__Actinobacteria.D_3__Micrococcales.D_4__Microbacteriaceae.D_5__Agromyces	D_0__Bacteria.D_1__Actinobacteria.D_2__Actinobacteria.D_3__Micrococcales.D_4__Brevibacteriaceae.D_5__Brevibacterium
D_0__Bacteria.D_1__Actinobacteria.D_2__Thermoleophilia.D_3__Solirubrobacterales.D_4__Solirubrobacteraceae.D_5__Patulibacter	D_0__Bacteria.D_1__Actinobacteria.D_2__Actinobacteria.D_3__Corynebacteriales.D_4__Corynebacteriaceae.D_5__Corynebacterium.1	D_0__Bacteria.D_1__Actinobacteria.D_2__Actinobacteria.D_3__Bifidobacteriales.D_4__Bifidobacteriaceae.D_5__Bifidobacterium	D_0__Bacteria.D_1__Actinobacteria.D_2__Actinobacteria.D_3__Bifidobacteriales.D_4__Bifidobacteriaceae.D_5__Gardnerella	D_0__Bacteria.D_1__Actinobacteria.D_2__Actinobacteria.D_3__Micrococcales.D_4__Microbacteriaceae.D_5__Microbacterium	D_0__Bacteria.D_1__Actinobacteria.D_2__Actinobacteria.D_3__Micrococcales.D_4__Dermatophilaceae.D_5__uncultured
D_0__Bacteria.D_1__Bacteroidetes.D_2__Bacteroidia.D_3__Bacteroidales.D_4__Bacteroidales.BS11.gut.group.D_5__uncultured.bacterium	D_0__Bacteria.D_1__Actinobacteria.D_2__Actinobacteria.D_3__Corynebacteriales.D_4__Nocardiaceae.D_5__Gordonia	D_0__Bacteria.D_1__Actinobacteria.D_2__Actinobacteria.D_3__Bifidobacteriales.D_4__Bifidobacteriaceae.D_5__Gardnerella	D_0__Bacteria.D_1__Actinobacteria.D_2__Actinobacteria.D_3__Corynebacteriales.D_4__Corynebacteriaceae.D_5__Corynebacterium	D_0__Bacteria.D_1__Actinobacteria.D_2__Actinobacteria.D_3__Micrococcales.D_4__Micrococcaceae.D_5__Glutamicibacter	D_0__Bacteria.D_1__Actinobacteria.D_2__Actinobacteria.D_3__Micrococcales.D_4__Microbacteriaceae.D_5__Pseudoclavibacter
D_0__Bacteria.D_1__Bacteroidetes.D_2__Bacteroidia.D_3__Bacteroidales.D_4__Bacteroidales.UCG.001.D_5__uncultured.rumen.bacterium	D_0__Bacteria.D_1__Actinobacteria.D_2__Actinobacteria.D_3__Corynebacteriales.D_4__Nocardiaceae.D_5__Rhodococcus	D_0__Bacteria.D_1__Actinobacteria.D_2__Actinobacteria.D_3__Corynebacteriales.D_4__Corynebacteriaceae.D_5__Corynebacterium.1	D_0__Bacteria.D_1__Actinobacteria.D_2__Actinobacteria.D_3__Corynebacteriales.D_4__Corynebacteriaceae.D_5__Corynebacterium.1	D_0__Bacteria.D_1__Actinobacteria.D_2__Actinobacteria.D_3__Micrococcales.D_4__Micrococcaceae.D_5__Yaniella	D_0__Bacteria.D_1__Actinobacteria.D_2__Actinobacteria.D_3__Micrococcales.D_4__Micrococcaceae.D_5__Micrococcus
D_0__Bacteria.D_1__Bacteroidetes.D_2__Bacteroidia.D_3__Bacteroidales.D_4__Barnesiellaceae.D_5__Barnesiella	D_0__Bacteria.D_1__Actinobacteria.D_2__Actinobacteria.D_3__Corynebacteriales.D_4__Nocardiaceae.D_5__Williamsia	D_0__Bacteria.D_1__Actinobacteria.D_2__Actinobacteria.D_3__Corynebacteriales.D_4__Mycobacteriaceae.D_5__Mycobacterium	D_0__Bacteria.D_1__Actinobacteria.D_2__Actinobacteria.D_3__Corynebacteriales.D_4__Corynebacteriaceae.__	D_0__Bacteria.D_1__Actinobacteria.D_2__Actinobacteria.D_3__Micrococcales.D_4__Micrococcales.Incertae.Sedis.D_5__Timonella	D_0__Bacteria.D_1__Actinobacteria.D_2__Actinobacteria.__.__.__
D_0__Bacteria.D_1__Bacteroidetes.D_2__Bacteroidia.D_3__Bacteroidales.D_4__F082.D_5__uncultured.bacterium	D_0__Bacteria.D_1__Actinobacteria.D_2__Actinobacteria.D_3__Micrococcales.D_4__Brevibacteriaceae.D_5__Brevibacterium	D_0__Bacteria.D_1__Actinobacteria.D_2__Actinobacteria.D_3__Corynebacteriales.D_4__Nocardiaceae.D_5__Rhodococcus	D_0__Bacteria.D_1__Actinobacteria.D_2__Actinobacteria.D_3__Corynebacteriales.D_4__Mycobacteriaceae.D_5__Mycobacterium	D_0__Bacteria.D_1__Actinobacteria.D_2__Actinobacteria.D_3__Micrococcales.__.__	D_0__Bacteria.D_1__Actinobacteria.D_2__Coriobacteriia.D_3__Coriobacteriales.D_4__Atopobiaceae.D_5__Olsenella
D_0__Bacteria.D_1__Bacteroidetes.D_2__Bacteroidia.D_3__Bacteroidales.D_4__Marinifilaceae.D_5__Butyricimonas	D_0__Bacteria.D_1__Actinobacteria.D_2__Actinobacteria.D_3__Micrococcales.D_4__Cellulomonadaceae.D_5__Oerskovia	D_0__Bacteria.D_1__Actinobacteria.D_2__Actinobacteria.D_3__Corynebacteriales.D_4__Nocardiaceae.D_5__Williamsia	D_0__Bacteria.D_1__Actinobacteria.D_2__Actinobacteria.D_3__Corynebacteriales.D_4__Nocardiaceae.D_5__Gordonia	D_0__Bacteria.D_1__Bacteroidetes.D_2__Bacteroidia.D_3__Bacteroidales.D_4__F082.D_5__uncultured.bacterium	D_0__Bacteria.D_1__Actinobacteria.D_2__Coriobacteriia.D_3__Coriobacteriales.D_4__Eggerthellaceae.D_5__Slackia
D_0__Bacteria.D_1__Bacteroidetes.D_2__Bacteroidia.D_3__Bacteroidales.D_4__Marinifilaceae.D_5__Odoribacter	D_0__Bacteria.D_1__Actinobacteria.D_2__Actinobacteria.D_3__Micrococcales.D_4__Dermabacteraceae.D_5__Dermabacter	D_0__Bacteria.D_1__Actinobacteria.D_2__Actinobacteria.D_3__Micrococcales.D_4__Brevibacteriaceae.D_5__Brevibacterium	D_0__Bacteria.D_1__Actinobacteria.D_2__Actinobacteria.D_3__Corynebacteriales.D_4__Nocardiaceae.D_5__Rhodococcus	D_0__Bacteria.D_1__Bacteroidetes.D_2__Bacteroidia.D_3__Bacteroidales.D_4__Prevotellaceae.D_5__Prevotellaceae.UCG.001	D_0__Bacteria.D_1__Actinobacteria.D_2__Coriobacteriia.D_3__Coriobacteriales.D_4__Eggerthellaceae.__
D_0__Bacteria.D_1__Bacteroidetes.D_2__Bacteroidia.D_3__Bacteroidales.D_4__Muribaculaceae.D_5__metagenome	D_0__Bacteria.D_1__Actinobacteria.D_2__Actinobacteria.D_3__Micrococcales.D_4__Dermacoccaceae.D_5__Kytococcus	D_0__Bacteria.D_1__Actinobacteria.D_2__Actinobacteria.D_3__Micrococcales.D_4__Cellulomonadaceae.D_5__Oerskovia	D_0__Bacteria.D_1__Actinobacteria.D_2__Actinobacteria.D_3__Corynebacteriales.D_4__Nocardiaceae.D_5__Williamsia	D_0__Bacteria.D_1__Bacteroidetes.D_2__Bacteroidia.D_3__Cytophagales.D_4__Cyclobacteriaceae.D_5__Algoriphagus	D_0__Bacteria.D_1__Bacteroidetes.D_2__Bacteroidia.D_3__Bacteroidales.D_4__Barnesiellaceae.D_5__Coprobacter
D_0__Bacteria.D_1__Bacteroidetes.D_2__Bacteroidia.D_3__Bacteroidales.D_4__Paludibacteraceae.D_5__F0058	D_0__Bacteria.D_1__Actinobacteria.D_2__Actinobacteria.D_3__Micrococcales.D_4__Microbacteriaceae.__	D_0__Bacteria.D_1__Actinobacteria.D_2__Actinobacteria.D_3__Micrococcales.D_4__Microbacteriaceae.__	D_0__Bacteria.D_1__Actinobacteria.D_2__Actinobacteria.D_3__Micrococcales.D_4__Brevibacteriaceae.D_5__Brevibacterium	D_0__Bacteria.D_1__Bacteroidetes.D_2__Bacteroidia.D_3__Cytophagales.D_4__Microscillaceae.D_5__Siphonobacter	D_0__Bacteria.D_1__Bacteroidetes.D_2__Bacteroidia.D_3__Bacteroidales.D_4__Muribaculaceae.D_5__uncultured.Porphyromonadaceae.bacterium
D_0__Bacteria.D_1__Bacteroidetes.D_2__Bacteroidia.D_3__Bacteroidales.D_4__Prevotellaceae.D_5__Paraprevotella	D_0__Bacteria.D_1__Actinobacteria.D_2__Actinobacteria.D_3__Micrococcales.D_4__Micrococcaceae.D_5__Arthrobacter	D_0__Bacteria.D_1__Actinobacteria.D_2__Actinobacteria.D_3__Micrococcales.D_4__Micrococcaceae.D_5__Arthrobacter	D_0__Bacteria.D_1__Actinobacteria.D_2__Actinobacteria.D_3__Micrococcales.D_4__Cellulomonadaceae.D_5__Oerskovia	D_0__Bacteria.D_1__Bacteroidetes.D_2__Bacteroidia.D_3__Cytophagales.D_4__Spirosomaceae.D_5__Dyadobacter	D_0__Bacteria.D_1__Bacteroidetes.D_2__Bacteroidia.D_3__Bacteroidales.D_4__Muribaculaceae.D_5__uncultured.organism
D_0__Bacteria.D_1__Bacteroidetes.D_2__Bacteroidia.D_3__Bacteroidales.D_4__Prevotellaceae.D_5__Prevotella.7	D_0__Bacteria.D_1__Actinobacteria.D_2__Actinobacteria.D_3__Micrococcales.D_4__Micrococcaceae.D_5__Glutamicibacter	D_0__Bacteria.D_1__Actinobacteria.D_2__Actinobacteria.D_3__Micrococcales.D_4__Micrococcaceae.D_5__Glutamicibacter	D_0__Bacteria.D_1__Actinobacteria.D_2__Actinobacteria.D_3__Micrococcales.D_4__Cellulomonadaceae.__	D_0__Bacteria.D_1__Bacteroidetes.D_2__Bacteroidia.D_3__Flavobacteriales.D_4__Flavobacteriaceae.D_5__Capnocytophaga	D_0__Bacteria.D_1__Bacteroidetes.D_2__Bacteroidia.D_3__Bacteroidales.D_4__Paludibacteraceae.D_5__F0058
D_0__Bacteria.D_1__Bacteroidetes.D_2__Bacteroidia.D_3__Bacteroidales.D_4__Prevotellaceae.__	D_0__Bacteria.D_1__Actinobacteria.D_2__Actinobacteria.D_3__Micrococcales.D_4__Micrococcaceae.D_5__Rothia	D_0__Bacteria.D_1__Actinobacteria.D_2__Actinobacteria.D_3__Micrococcales.D_4__Micrococcaceae.D_5__Micrococcus	D_0__Bacteria.D_1__Actinobacteria.D_2__Actinobacteria.D_3__Micrococcales.D_4__Dermabacteraceae.D_5__Dermabacter	D_0__Bacteria.D_1__Bacteroidetes.D_2__Bacteroidia.D_3__Flavobacteriales.D_4__Weeksellaceae.D_5__Empedobacter	D_0__Bacteria.D_1__Bacteroidetes.D_2__Bacteroidia.D_3__Bacteroidales.D_4__Prevotellaceae.D_5__Paraprevotella
D_0__Bacteria.D_1__Bacteroidetes.D_2__Bacteroidia.D_3__Bacteroidales.D_4__Rikenellaceae.D_5__Alistipes	D_0__Bacteria.D_1__Actinobacteria.D_2__Actinobacteria.D_3__Micrococcales.D_4__Micrococcaceae.__	D_0__Bacteria.D_1__Actinobacteria.D_2__Actinobacteria.D_3__Micrococcales.D_4__Micrococcaceae.D_5__Rothia	D_0__Bacteria.D_1__Actinobacteria.D_2__Actinobacteria.D_3__Micrococcales.D_4__Dermatophilaceae.D_5__uncultured	D_0__Bacteria.D_1__Firmicutes.D_2__Bacilli.D_3__Bacillales.D_4__Family.XI.D_5__Gemella	D_0__Bacteria.D_1__Bacteroidetes.D_2__Bacteroidia.D_3__Bacteroidales.D_4__Prevotellaceae.D_5__Prevotellaceae.NK3B31.group
D_0__Bacteria.D_1__Bacteroidetes.D_2__Bacteroidia.D_3__Bacteroidales.D_4__Tannerellaceae.D_5__Tannerella	D_0__Bacteria.D_1__Actinobacteria.D_2__Actinobacteria.D_3__Micrococcales.D_4__Micrococcales.Incertae.Sedis.D_5__Timonella	D_0__Bacteria.D_1__Actinobacteria.D_2__Actinobacteria.D_3__Micrococcales.D_4__Micrococcaceae.__	D_0__Bacteria.D_1__Actinobacteria.D_2__Actinobacteria.D_3__Micrococcales.D_4__Microbacteriaceae.D_5__Agromyces	D_0__Bacteria.D_1__Firmicutes.D_2__Bacilli.D_3__Bacillales.D_4__Paenibacillaceae.__	D_0__Bacteria.D_1__Bacteroidetes.D_2__Bacteroidia.D_3__Bacteroidales.D_4__Prevotellaceae.D_5__Prevotellaceae.UCG.003
D_0__Bacteria.D_1__Bacteroidetes.D_2__Bacteroidia.D_3__Cytophagales.D_4__Cyclobacteriaceae.D_5__Algoriphagus	D_0__Bacteria.D_1__Actinobacteria.D_2__Actinobacteria.D_3__Micrococcales.D_4__Sanguibacteraceae.D_5__Sanguibacter	D_0__Bacteria.D_1__Actinobacteria.D_2__Actinobacteria.D_3__Micrococcales.D_4__Micrococcales.Incertae.Sedis.D_5__Timonella	D_0__Bacteria.D_1__Actinobacteria.D_2__Actinobacteria.D_3__Micrococcales.D_4__Microbacteriaceae.D_5__Microbacterium	D_0__Bacteria.D_1__Firmicutes.D_2__Bacilli.D_3__Bacillales.D_4__Staphylococcaceae.D_5__Nosocomiicoccus	D_0__Bacteria.D_1__Bacteroidetes.D_2__Bacteroidia.D_3__Bacteroidales.D_4__p.2534.18B5.gut.group.D_5__uncultured.bacterium
D_0__Bacteria.D_1__Bacteroidetes.D_2__Bacteroidia.D_3__Cytophagales.D_4__Spirosomaceae.__	D_0__Bacteria.D_1__Actinobacteria.D_2__Actinobacteria.D_3__Propionibacteriales.D_4__Nocardioidaceae.D_5__Nocardioides	D_0__Bacteria.D_1__Actinobacteria.D_2__Actinobacteria.D_3__Micrococcales.D_4__Sanguibacteraceae.D_5__Sanguibacter	D_0__Bacteria.D_1__Actinobacteria.D_2__Actinobacteria.D_3__Micrococcales.D_4__Microbacteriaceae.D_5__Pseudoclavibacter	D_0__Bacteria.D_1__Firmicutes.D_2__Clostridia.D_3__Clostridiales.D_4__Christensenellaceae.__	D_0__Bacteria.D_1__Bacteroidetes.D_2__Bacteroidia.D_3__Bacteroidales.D_4__uncultured.D_5__uncultured.bacterium
D_0__Bacteria.D_1__Bacteroidetes.D_2__Bacteroidia.D_3__Flavobacteriales.D_4__Weeksellaceae.D_5__Bergeyella	D_0__Bacteria.D_1__Actinobacteria.D_2__Coriobacteriia.D_3__Coriobacteriales.D_4__Atopobiaceae.D_5__Atopobium	D_0__Bacteria.D_1__Actinobacteria.D_2__Actinobacteria.D_3__Propionibacteriales.D_4__Nocardioidaceae.D_5__Nocardioides	D_0__Bacteria.D_1__Actinobacteria.D_2__Actinobacteria.D_3__Micrococcales.D_4__Microbacteriaceae.__	D_0__Bacteria.D_1__Firmicutes.D_2__Clostridia.D_3__Clostridiales.D_4__Lachnospiraceae.D_5__Lachnospiraceae.UCG.001	D_0__Bacteria.D_1__Bacteroidetes.D_2__Bacteroidia.D_3__Chitinophagales.D_4__Chitinophagaceae.D_5__Sediminibacterium
D_0__Bacteria.D_1__Bacteroidetes.D_2__Bacteroidia.D_3__Sphingobacteriales.D_4__Lentimicrobiaceae.D_5__uncultured.eubacterium.E1.K9	D_0__Bacteria.D_1__Actinobacteria.D_2__Coriobacteriia.D_3__Coriobacteriales.D_4__Atopobiaceae.D_5__Libanicoccus	D_0__Bacteria.D_1__Actinobacteria.D_2__Actinobacteria.__.__.__	D_0__Bacteria.D_1__Actinobacteria.D_2__Actinobacteria.D_3__Micrococcales.D_4__Micrococcaceae.D_5__Arthrobacter	D_0__Bacteria.D_1__Firmicutes.D_2__Clostridia.D_3__Clostridiales.D_4__Peptococcaceae.D_5__uncultured	D_0__Bacteria.D_1__Bacteroidetes.D_2__Bacteroidia.D_3__Chitinophagales.D_4__Chitinophagaceae.D_5__Taibaiella
D_0__Bacteria.D_1__Firmicutes.D_2__Bacilli.D_3__Bacillales.D_4__Family.XI.D_5__Gemella	D_0__Bacteria.D_1__Actinobacteria.D_2__Coriobacteriia.D_3__Coriobacteriales.D_4__Atopobiaceae.D_5__Olsenella	D_0__Bacteria.D_1__Actinobacteria.D_2__Coriobacteriia.D_3__Coriobacteriales.D_4__Atopobiaceae.D_5__Atopobium	D_0__Bacteria.D_1__Actinobacteria.D_2__Actinobacteria.D_3__Micrococcales.D_4__Micrococcaceae.D_5__Glutamicibacter	D_0__Bacteria.D_1__Firmicutes.D_2__Clostridia.D_3__Clostridiales.D_4__Peptostreptococcaceae.D_5__Sporacetigenium	D_0__Bacteria.D_1__Bacteroidetes.D_2__Bacteroidia.D_3__Chitinophagales.D_4__Chitinophagaceae.D_5__Terrimonas
D_0__Bacteria.D_1__Firmicutes.D_2__Bacilli.D_3__Bacillales.D_4__Planococcaceae.D_5__Psychrobacillus	D_0__Bacteria.D_1__Actinobacteria.D_2__Coriobacteriia.D_3__Coriobacteriales.D_4__Coriobacteriaceae.D_5__Collinsella	D_0__Bacteria.D_1__Actinobacteria.D_2__Coriobacteriia.D_3__Coriobacteriales.D_4__Atopobiaceae.D_5__Libanicoccus	D_0__Bacteria.D_1__Actinobacteria.D_2__Actinobacteria.D_3__Micrococcales.D_4__Micrococcaceae.D_5__Micrococcus	D_0__Bacteria.D_1__Firmicutes.D_2__Clostridia.D_3__Clostridiales.D_4__Ruminococcaceae.D_5__Ruminiclostridium.9	D_0__Bacteria.D_1__Bacteroidetes.D_2__Bacteroidia.D_3__Cytophagales.D_4__Spirosomaceae.D_5__Flectobacillus
D_0__Bacteria.D_1__Firmicutes.D_2__Bacilli.D_3__Bacillales.D_4__Planococcaceae.D_5__Rummeliibacillus	D_0__Bacteria.D_1__Actinobacteria.D_2__Coriobacteriia.D_3__Coriobacteriales.D_4__Coriobacteriales.Incertae.Sedis.D_5__uncultured	D_0__Bacteria.D_1__Actinobacteria.D_2__Coriobacteriia.D_3__Coriobacteriales.D_4__Atopobiaceae.D_5__Olsenella	D_0__Bacteria.D_1__Actinobacteria.D_2__Actinobacteria.D_3__Micrococcales.D_4__Micrococcaceae.D_5__Rothia	D_0__Bacteria.D_1__Firmicutes.D_2__Erysipelotrichia.D_3__Erysipelotrichales.D_4__Erysipelotrichaceae.D_5__.Clostridium..innocuum.group	D_0__Bacteria.D_1__Bacteroidetes.D_2__Bacteroidia.D_3__Cytophagales.D_4__Spirosomaceae.D_5__Spirosoma
D_0__Bacteria.D_1__Firmicutes.D_2__Bacilli.D_3__Lactobacillales.D_4__Aerococcaceae.D_5__Abiotrophia	D_0__Bacteria.D_1__Actinobacteria.D_2__Coriobacteriia.D_3__Coriobacteriales.D_4__Eggerthellaceae.D_5__DNF00809	D_0__Bacteria.D_1__Actinobacteria.D_2__Coriobacteriia.D_3__Coriobacteriales.D_4__Atopobiaceae.D_5__uncultured	D_0__Bacteria.D_1__Actinobacteria.D_2__Actinobacteria.D_3__Micrococcales.D_4__Micrococcaceae.D_5__Yaniella	D_0__Bacteria.D_1__Planctomycetes.D_2__Planctomycetacia.D_3__Isosphaerales.D_4__Isosphaeraceae.D_5__Isosphaera	D_0__Bacteria.D_1__Bacteroidetes.D_2__Bacteroidia.D_3__Flavobacteriales.D_4__Weeksellaceae.D_5__Moheibacter
D_0__Bacteria.D_1__Firmicutes.D_2__Bacilli.__.__.__	D_0__Bacteria.D_1__Actinobacteria.D_2__Coriobacteriia.D_3__Coriobacteriales.D_4__Eggerthellaceae.D_5__Enterorhabdus	D_0__Bacteria.D_1__Actinobacteria.D_2__Coriobacteriia.D_3__Coriobacteriales.D_4__Coriobacteriaceae.D_5__Collinsella	D_0__Bacteria.D_1__Actinobacteria.D_2__Actinobacteria.D_3__Micrococcales.D_4__Micrococcales.Incertae.Sedis.D_5__Timonella	D_0__Bacteria.D_1__Proteobacteria.D_2__Alphaproteobacteria.D_3__Rhizobiales.D_4__Beijerinckiaceae.D_5__28.YEA.48	D_0__Bacteria.D_1__Bacteroidetes.D_2__Bacteroidia.D_3__Sphingobacteriales.D_4__Lentimicrobiaceae.D_5__Bacteroidia.bacterium.feline.oral.taxon.312
D_0__Bacteria.D_1__Firmicutes.D_2__Clostridia.D_3__Clostridiales.D_4__Clostridiales.vadinBB60.group.D_5__uncultured.Thermoanaerobactera	D_0__Bacteria.D_1__Actinobacteria.D_2__Coriobacteriia.D_3__Coriobacteriales.D_4__Eggerthellaceae.D_5__Senegalimassilia	D_0__Bacteria.D_1__Actinobacteria.D_2__Coriobacteriia.D_3__Coriobacteriales.D_4__Coriobacteriales.Incertae.Sedis.D_5__Raoultibacter	D_0__Bacteria.D_1__Actinobacteria.D_2__Actinobacteria.D_3__Micrococcales.D_4__Sanguibacteraceae.D_5__Sanguibacter	D_0__Bacteria.D_1__Proteobacteria.D_2__Alphaproteobacteria.D_3__Rhodobacterales.D_4__Rhodobacteraceae.D_5__uncultured	D_0__Bacteria.D_1__Bacteroidetes.D_2__Bacteroidia.D_3__Sphingobacteriales.D_4__Sphingobacteriaceae.D_5__Nubsella
D_0__Bacteria.D_1__Firmicutes.D_2__Clostridia.D_3__Clostridiales.D_4__Clostridiales.vadinBB60.group.D_5__uncultured.bacterium	D_0__Bacteria.D_1__Actinobacteria.D_2__Coriobacteriia.D_3__Coriobacteriales.D_4__Eggerthellaceae.D_5__Slackia	D_0__Bacteria.D_1__Actinobacteria.D_2__Coriobacteriia.D_3__Coriobacteriales.D_4__Coriobacteriales.Incertae.Sedis.D_5__uncultured	D_0__Bacteria.D_1__Actinobacteria.D_2__Actinobacteria.D_3__Micrococcales.__.__	D_0__Bacteria.D_1__Proteobacteria.D_2__Alphaproteobacteria.D_3__Sneathiellales.D_4__Sneathiellaceae.D_5__Taonella	D_0__Bacteria.D_1__Cyanobacteria.D_2__Melainabacteria.D_3__Obscuribacterales.__.__
D_0__Bacteria.D_1__Firmicutes.D_2__Clostridia.D_3__Clostridiales.D_4__Family.XIII.D_5__Anaerovorax	D_0__Bacteria.D_1__Actinobacteria.D_2__Coriobacteriia.D_3__Coriobacteriales.D_4__Eggerthellaceae.D_5__uncultured	D_0__Bacteria.D_1__Actinobacteria.D_2__Coriobacteriia.D_3__Coriobacteriales.D_4__Eggerthellaceae.D_5__DNF00809	D_0__Bacteria.D_1__Actinobacteria.D_2__Actinobacteria.D_3__Propionibacteriales.D_4__Nocardioidaceae.D_5__Aeromicrobium	D_0__Bacteria.D_1__Proteobacteria.D_2__Alphaproteobacteria.D_3__Sphingomonadales.D_4__Sphingomonadaceae.D_5__Altererythrobacter	D_0__Bacteria.D_1__Cyanobacteria.D_2__Oxyphotobacteria.D_3__Chloroplast.__.__
D_0__Bacteria.D_1__Firmicutes.D_2__Clostridia.D_3__Clostridiales.D_4__Family.XIII.D_5__Family.XIII.AD3011.group	D_0__Bacteria.D_1__Actinobacteria.D_2__Thermoleophilia.D_3__Solirubrobacterales.D_4__Solirubrobacteraceae.D_5__Patulibacter	D_0__Bacteria.D_1__Actinobacteria.D_2__Coriobacteriia.D_3__Coriobacteriales.D_4__Eggerthellaceae.D_5__Enterorhabdus	D_0__Bacteria.D_1__Actinobacteria.D_2__Actinobacteria.D_3__Propionibacteriales.D_4__Nocardioidaceae.D_5__Nocardioides	D_0__Bacteria.D_1__Proteobacteria.D_2__Alphaproteobacteria.D_3__Sphingomonadales.D_4__Sphingomonadaceae.D_5__Sphingobium	D_0__Bacteria.D_1__Epsilonbacteraeota.D_2__Campylobacteria.D_3__Campylobacterales.D_4__Helicobacteraceae.D_5__Helicobacter
D_0__Bacteria.D_1__Firmicutes.D_2__Clostridia.D_3__Clostridiales.D_4__Family.XIII.D_5__.Eubacterium..brachy.group	D_0__Bacteria.D_1__Bacteroidetes.D_2__Bacteroidia.D_3__Bacteroidales.D_4__Bacteroidaceae.D_5__Bacteroides	D_0__Bacteria.D_1__Actinobacteria.D_2__Coriobacteriia.D_3__Coriobacteriales.D_4__Eggerthellaceae.D_5__Senegalimassilia	D_0__Bacteria.D_1__Actinobacteria.D_2__Actinobacteria.__.__.__	D_0__Bacteria.D_1__Proteobacteria.D_2__Gammaproteobacteria.D_3__Aeromonadales.D_4__Succinivibrionaceae.__	D_0__Bacteria.D_1__Firmicutes.D_2__Bacilli.D_3__Bacillales.D_4__Paenibacillaceae.D_5__Paenibacillus
D_0__Bacteria.D_1__Firmicutes.D_2__Clostridia.D_3__Clostridiales.D_4__Family.XIII.D_5__.Eubacterium..nodatum.group	D_0__Bacteria.D_1__Bacteroidetes.D_2__Bacteroidia.D_3__Bacteroidales.D_4__Bacteroidales.BS11.gut.group.D_5__uncultured.bacterium	D_0__Bacteria.D_1__Actinobacteria.D_2__Coriobacteriia.D_3__Coriobacteriales.D_4__Eggerthellaceae.D_5__Slackia	D_0__Bacteria.D_1__Actinobacteria.D_2__Coriobacteriia.D_3__Coriobacteriales.D_4__Atopobiaceae.D_5__Atopobium	D_0__Bacteria.D_1__Proteobacteria.D_2__Gammaproteobacteria.D_3__Betaproteobacteriales.D_4__Burkholderiaceae.D_5__Duganella	D_0__Bacteria.D_1__Firmicutes.D_2__Bacilli.D_3__Lactobacillales.D_4__Leuconostocaceae.D_5__Leuconostoc
D_0__Bacteria.D_1__Firmicutes.D_2__Clostridia.D_3__Clostridiales.D_4__Family.XIII.D_5__.Eubacterium..saphenum.group	D_0__Bacteria.D_1__Bacteroidetes.D_2__Bacteroidia.D_3__Bacteroidales.D_4__Barnesiellaceae.D_5__Barnesiella	D_0__Bacteria.D_1__Actinobacteria.D_2__Coriobacteriia.D_3__Coriobacteriales.D_4__Eggerthellaceae.D_5__uncultured	D_0__Bacteria.D_1__Actinobacteria.D_2__Coriobacteriia.D_3__Coriobacteriales.D_4__Atopobiaceae.D_5__Coriobacteriaceae.UCG.002	D_0__Bacteria.D_1__Proteobacteria.D_2__Gammaproteobacteria.D_3__Betaproteobacteriales.D_4__Burkholderiaceae.D_5__Noviherbaspirillum	D_0__Bacteria.D_1__Firmicutes.D_2__Clostridia.D_3__Clostridiales.D_4__Clostridiales.vadinBB60.group.D_5__gut.metagenome
D_0__Bacteria.D_1__Firmicutes.D_2__Clostridia.D_3__Clostridiales.D_4__Family.XIII.D_5__uncultured	D_0__Bacteria.D_1__Bacteroidetes.D_2__Bacteroidia.D_3__Bacteroidales.D_4__Marinifilaceae.D_5__Odoribacter	D_0__Bacteria.D_1__Actinobacteria.D_2__Rubrobacteria.D_3__Rubrobacterales.D_4__Rubrobacteriaceae.D_5__Rubrobacter	D_0__Bacteria.D_1__Actinobacteria.D_2__Coriobacteriia.D_3__Coriobacteriales.D_4__Atopobiaceae.D_5__Olsenella	D_0__Bacteria.D_1__Proteobacteria.D_2__Gammaproteobacteria.D_3__Betaproteobacteriales.D_4__Burkholderiaceae.D_5__Undibacterium	D_0__Bacteria.D_1__Firmicutes.D_2__Clostridia.D_3__Clostridiales.D_4__Family.XI.D_5__Finegoldia
D_0__Bacteria.D_1__Firmicutes.D_2__Clostridia.D_3__Clostridiales.D_4__Lachnospiraceae.D_5__Anaerostipes	D_0__Bacteria.D_1__Bacteroidetes.D_2__Bacteroidia.D_3__Bacteroidales.D_4__Muribaculaceae.D_5__uncultured.Porphyromonadaceae.bacterium	D_0__Bacteria.D_1__Actinobacteria.D_2__Thermoleophilia.D_3__Solirubrobacterales.D_4__Solirubrobacteraceae.D_5__Patulibacter	D_0__Bacteria.D_1__Actinobacteria.D_2__Coriobacteriia.D_3__Coriobacteriales.D_4__Coriobacteriaceae.D_5__Collinsella	D_0__Bacteria.D_1__Proteobacteria.D_2__Gammaproteobacteria.D_3__Betaproteobacteriales.D_4__Burkholderiaceae.D_5__uncultured	D_0__Bacteria.D_1__Firmicutes.D_2__Clostridia.D_3__Clostridiales.D_4__Family.XI.D_5__Parvimonas
D_0__Bacteria.D_1__Firmicutes.D_2__Clostridia.D_3__Clostridiales.D_4__Lachnospiraceae.D_5__Butyrivibrio	D_0__Bacteria.D_1__Bacteroidetes.D_2__Bacteroidia.D_3__Bacteroidales.D_4__Porphyromonadaceae.D_5__Porphyromonas	D_0__Bacteria.D_1__Bacteroidetes.D_2__Bacteroidia.D_3__Bacteroidales.D_4__Bacteroidaceae.D_5__Bacteroides	D_0__Bacteria.D_1__Actinobacteria.D_2__Coriobacteriia.D_3__Coriobacteriales.D_4__Eggerthellaceae.D_5__DNF00809	D_0__Bacteria.D_1__Proteobacteria.D_2__Gammaproteobacteria.D_3__Betaproteobacteriales.D_4__Methylophilaceae.D_5__Methylophilus	D_0__Bacteria.D_1__Firmicutes.D_2__Clostridia.D_3__Clostridiales.D_4__Family.XI.D_5__W5053
D_0__Bacteria.D_1__Firmicutes.D_2__Clostridia.D_3__Clostridiales.D_4__Lachnospiraceae.D_5__Catonella	D_0__Bacteria.D_1__Bacteroidetes.D_2__Bacteroidia.D_3__Bacteroidales.D_4__Prevotellaceae.D_5__Alloprevotella	D_0__Bacteria.D_1__Bacteroidetes.D_2__Bacteroidia.D_3__Bacteroidales.D_4__Bacteroidales.BS11.gut.group.D_5__uncultured.bacterium	D_0__Bacteria.D_1__Actinobacteria.D_2__Coriobacteriia.D_3__Coriobacteriales.D_4__Eggerthellaceae.D_5__Enterorhabdus	D_0__Bacteria.D_1__Proteobacteria.D_2__Gammaproteobacteria.D_3__Betaproteobacteriales.D_4__Rhodocyclaceae.D_5__C39	D_0__Bacteria.D_1__Firmicutes.D_2__Clostridia.D_3__Clostridiales.D_4__Family.XIII.D_5__S5.A14a
D_0__Bacteria.D_1__Firmicutes.D_2__Clostridia.D_3__Clostridiales.D_4__Lachnospiraceae.D_5__Cellulosilyticum	D_0__Bacteria.D_1__Bacteroidetes.D_2__Bacteroidia.D_3__Bacteroidales.D_4__Prevotellaceae.D_5__Prevotella	D_0__Bacteria.D_1__Bacteroidetes.D_2__Bacteroidia.D_3__Bacteroidales.D_4__F082.__	D_0__Bacteria.D_1__Actinobacteria.D_2__Coriobacteriia.D_3__Coriobacteriales.D_4__Eggerthellaceae.D_5__Gordonibacter	D_0__Bacteria.D_1__Proteobacteria.D_2__Gammaproteobacteria.D_3__Cellvibrionales.D_4__Cellvibrionaceae.D_5__Cellvibrio	D_0__Bacteria.D_1__Firmicutes.D_2__Clostridia.D_3__Clostridiales.D_4__Lachnospiraceae.D_5__Howardella
D_0__Bacteria.D_1__Firmicutes.D_2__Clostridia.D_3__Clostridiales.D_4__Lachnospiraceae.D_5__Fusicatenibacter	D_0__Bacteria.D_1__Bacteroidetes.D_2__Bacteroidia.D_3__Bacteroidales.D_4__Prevotellaceae.D_5__Prevotella.2	D_0__Bacteria.D_1__Bacteroidetes.D_2__Bacteroidia.D_3__Bacteroidales.D_4__Muribaculaceae.D_5__metagenome	D_0__Bacteria.D_1__Actinobacteria.D_2__Coriobacteriia.D_3__Coriobacteriales.D_4__Eggerthellaceae.D_5__Slackia	D_0__Bacteria.D_1__Proteobacteria.D_2__Gammaproteobacteria.D_3__Enterobacteriales.D_4__Enterobacteriaceae.D_5__Buttiauxella	D_0__Bacteria.D_1__Firmicutes.D_2__Clostridia.D_3__Clostridiales.D_4__Lachnospiraceae.D_5__Lachnospiraceae.NC2004.group
D_0__Bacteria.D_1__Firmicutes.D_2__Clostridia.D_3__Clostridiales.D_4__Lachnospiraceae.D_5__Johnsonella	D_0__Bacteria.D_1__Bacteroidetes.D_2__Bacteroidia.D_3__Bacteroidales.D_4__Prevotellaceae.D_5__Prevotella.6	D_0__Bacteria.D_1__Bacteroidetes.D_2__Bacteroidia.D_3__Bacteroidales.D_4__Muribaculaceae.D_5__uncultured.Porphyromonadaceae.bacterium	D_0__Bacteria.D_1__Actinobacteria.D_2__Coriobacteriia.D_3__Coriobacteriales.D_4__Eggerthellaceae.__	D_0__Bacteria.D_1__Proteobacteria.D_2__Gammaproteobacteria.D_3__Pasteurellales.D_4__Pasteurellaceae.D_5__Aggregatibacter	D_0__Bacteria.D_1__Firmicutes.D_2__Clostridia.D_3__Clostridiales.D_4__Lachnospiraceae.D_5__Lachnospiraceae.UCG.006
D_0__Bacteria.D_1__Firmicutes.D_2__Clostridia.D_3__Clostridiales.D_4__Lachnospiraceae.D_5__Lachnoanaerobaculum	D_0__Bacteria.D_1__Bacteroidetes.D_2__Bacteroidia.D_3__Bacteroidales.D_4__Prevotellaceae.D_5__Prevotella.9	D_0__Bacteria.D_1__Bacteroidetes.D_2__Bacteroidia.D_3__Bacteroidales.D_4__Porphyromonadaceae.D_5__Porphyromonas	D_0__Bacteria.D_1__Bacteroidetes.D_2__Bacteroidia.D_3__Bacteroidales.D_4__Bacteroidaceae.D_5__Bacteroides	D_0__Bacteria.D_1__Proteobacteria.D_2__Gammaproteobacteria.D_3__Xanthomonadales.D_4__Rhodanobacteraceae.D_5__Dokdonella	D_0__Bacteria.D_1__Firmicutes.D_2__Clostridia.D_3__Clostridiales.D_4__Lachnospiraceae.D_5__Marvinbryantia
D_0__Bacteria.D_1__Firmicutes.D_2__Clostridia.D_3__Clostridiales.D_4__Lachnospiraceae.D_5__Lachnoclostridium	D_0__Bacteria.D_1__Bacteroidetes.D_2__Bacteroidia.D_3__Bacteroidales.D_4__Prevotellaceae.D_5__Prevotellaceae.UCG.004	D_0__Bacteria.D_1__Bacteroidetes.D_2__Bacteroidia.D_3__Bacteroidales.D_4__Prevotellaceae.D_5__Alloprevotella	D_0__Bacteria.D_1__Bacteroidetes.D_2__Bacteroidia.D_3__Bacteroidales.D_4__Barnesiellaceae.D_5__Coprobacter	D_0__Bacteria.D_1__Proteobacteria.__.__.__.__	D_0__Bacteria.D_1__Firmicutes.D_2__Clostridia.D_3__Clostridiales.D_4__Lachnospiraceae.D_5__Moryella
D_0__Bacteria.D_1__Firmicutes.D_2__Clostridia.D_3__Clostridiales.D_4__Lachnospiraceae.D_5__Lachnoclostridium.10	D_0__Bacteria.D_1__Bacteroidetes.D_2__Bacteroidia.D_3__Bacteroidales.D_4__Prevotellaceae.D_5__uncultured	D_0__Bacteria.D_1__Bacteroidetes.D_2__Bacteroidia.D_3__Bacteroidales.D_4__Prevotellaceae.D_5__Prevotella	D_0__Bacteria.D_1__Bacteroidetes.D_2__Bacteroidia.D_3__Bacteroidales.D_4__F082.D_5__uncultured.bacterium		D_0__Bacteria.D_1__Firmicutes.D_2__Clostridia.D_3__Clostridiales.D_4__Lachnospiraceae.D_5__.Eubacterium..eligens.group
D_0__Bacteria.D_1__Firmicutes.D_2__Clostridia.D_3__Clostridiales.D_4__Lachnospiraceae.D_5__Lachnospiraceae.AC2044.group	D_0__Bacteria.D_1__Bacteroidetes.D_2__Bacteroidia.D_3__Bacteroidales.D_4__Rikenellaceae.D_5__Rikenellaceae.RC9.gut.group	D_0__Bacteria.D_1__Bacteroidetes.D_2__Bacteroidia.D_3__Bacteroidales.D_4__Prevotellaceae.D_5__Prevotella.2	D_0__Bacteria.D_1__Bacteroidetes.D_2__Bacteroidia.D_3__Bacteroidales.D_4__Marinifilaceae.D_5__Odoribacter		D_0__Bacteria.D_1__Firmicutes.D_2__Clostridia.D_3__Clostridiales.D_4__Lachnospiraceae.D_5__.Eubacterium..ruminantium.group
D_0__Bacteria.D_1__Firmicutes.D_2__Clostridia.D_3__Clostridiales.D_4__Lachnospiraceae.D_5__Lachnospiraceae.UCG.004	D_0__Bacteria.D_1__Bacteroidetes.D_2__Bacteroidia.D_3__Bacteroidales.D_4__Tannerellaceae.D_5__Parabacteroides	D_0__Bacteria.D_1__Bacteroidetes.D_2__Bacteroidia.D_3__Bacteroidales.D_4__Prevotellaceae.D_5__Prevotella.6	D_0__Bacteria.D_1__Bacteroidetes.D_2__Bacteroidia.D_3__Bacteroidales.D_4__Muribaculaceae.Ambiguous_taxa		D_0__Bacteria.D_1__Firmicutes.D_2__Clostridia.D_3__Clostridiales.D_4__Peptococcaceae.D_5__Peptococcus
D_0__Bacteria.D_1__Firmicutes.D_2__Clostridia.D_3__Clostridiales.D_4__Lachnospiraceae.D_5__Lachnospiraceae.UCG.010	D_0__Bacteria.D_1__Bacteroidetes.D_2__Bacteroidia.D_3__Bacteroidales.D_4__uncultured.D_5__uncultured.bacterium	D_0__Bacteria.D_1__Bacteroidetes.D_2__Bacteroidia.D_3__Bacteroidales.D_4__Prevotellaceae.D_5__Prevotella.7	D_0__Bacteria.D_1__Bacteroidetes.D_2__Bacteroidia.D_3__Bacteroidales.D_4__Muribaculaceae.D_5__Muribaculum		D_0__Bacteria.D_1__Firmicutes.D_2__Clostridia.D_3__Clostridiales.D_4__Ruminococcaceae.D_5__Butyricicoccus
D_0__Bacteria.D_1__Firmicutes.D_2__Clostridia.D_3__Clostridiales.D_4__Lachnospiraceae.D_5__Oribacterium	D_0__Bacteria.D_1__Bacteroidetes.D_2__Bacteroidia.D_3__Chitinophagales.D_4__Chitinophagaceae.D_5__Chitinophaga	D_0__Bacteria.D_1__Bacteroidetes.D_2__Bacteroidia.D_3__Bacteroidales.D_4__Prevotellaceae.D_5__Prevotella.9	D_0__Bacteria.D_1__Bacteroidetes.D_2__Bacteroidia.D_3__Bacteroidales.D_4__Muribaculaceae.D_5__gut.metagenome		D_0__Bacteria.D_1__Firmicutes.D_2__Clostridia.D_3__Clostridiales.D_4__Ruminococcaceae.D_5__Ruminiclostridium.6
D_0__Bacteria.D_1__Firmicutes.D_2__Clostridia.D_3__Clostridiales.D_4__Lachnospiraceae.D_5__Stomatobaculum	D_0__Bacteria.D_1__Bacteroidetes.D_2__Bacteroidia.D_3__Chitinophagales.D_4__Chitinophagaceae.D_5__Niabella	D_0__Bacteria.D_1__Bacteroidetes.D_2__Bacteroidia.D_3__Bacteroidales.D_4__Prevotellaceae.D_5__Prevotellaceae.UCG.004	D_0__Bacteria.D_1__Bacteroidetes.D_2__Bacteroidia.D_3__Bacteroidales.D_4__Muribaculaceae.D_5__metagenome		D_0__Bacteria.D_1__Firmicutes.D_2__Clostridia.D_3__Clostridiales.D_4__Ruminococcaceae.D_5__Ruminococcaceae.UCG.010
D_0__Bacteria.D_1__Firmicutes.D_2__Clostridia.D_3__Clostridiales.D_4__Lachnospiraceae.D_5__.Eubacterium..ventriosum.group	D_0__Bacteria.D_1__Bacteroidetes.D_2__Bacteroidia.D_3__Chitinophagales.D_4__Chitinophagaceae.D_5__Pseudoflavitalea	D_0__Bacteria.D_1__Bacteroidetes.D_2__Bacteroidia.D_3__Bacteroidales.D_4__Prevotellaceae.D_5__uncultured	D_0__Bacteria.D_1__Bacteroidetes.D_2__Bacteroidia.D_3__Bacteroidales.D_4__Muribaculaceae.D_5__uncultured.Porphyromonadaceae.bacterium		D_0__Bacteria.D_1__Firmicutes.D_2__Clostridia.D_3__Clostridiales.D_4__Ruminococcaceae.D_5__Ruminococcaceae.UCG.013
D_0__Bacteria.D_1__Firmicutes.D_2__Clostridia.D_3__Clostridiales.D_4__Peptostreptococcaceae.D_5__Filifactor	D_0__Bacteria.D_1__Bacteroidetes.D_2__Bacteroidia.D_3__Chitinophagales.D_4__Chitinophagaceae.D_5__Terrimonas	D_0__Bacteria.D_1__Bacteroidetes.D_2__Bacteroidia.D_3__Bacteroidales.D_4__Rikenellaceae.D_5__Alistipes	D_0__Bacteria.D_1__Bacteroidetes.D_2__Bacteroidia.D_3__Bacteroidales.D_4__Muribaculaceae.D_5__uncultured.bacterium		D_0__Bacteria.D_1__Firmicutes.D_2__Clostridia.D_3__Clostridiales.D_4__Ruminococcaceae.D_5__Ruminococcus.1
D_0__Bacteria.D_1__Firmicutes.D_2__Clostridia.D_3__Clostridiales.D_4__Peptostreptococcaceae.__	D_0__Bacteria.D_1__Bacteroidetes.D_2__Bacteroidia.D_3__Cytophagales.D_4__Spirosomaceae.D_5__Dyadobacter	D_0__Bacteria.D_1__Bacteroidetes.D_2__Bacteroidia.D_3__Bacteroidales.D_4__Rikenellaceae.D_5__Rikenellaceae.RC9.gut.group	D_0__Bacteria.D_1__Bacteroidetes.D_2__Bacteroidia.D_3__Bacteroidales.D_4__Muribaculaceae.D_5__uncultured.organism		D_0__Bacteria.D_1__Firmicutes.D_2__Clostridia.D_3__Clostridiales.D_4__Ruminococcaceae.__
D_0__Bacteria.D_1__Firmicutes.D_2__Erysipelotrichia.D_3__Erysipelotrichales.D_4__Erysipelotrichaceae.D_5__Bulleidia	D_0__Bacteria.D_1__Bacteroidetes.D_2__Bacteroidia.D_3__Cytophagales.D_4__Spirosomaceae.D_5__Leadbetterella	D_0__Bacteria.D_1__Bacteroidetes.D_2__Bacteroidia.D_3__Bacteroidales.D_4__Tannerellaceae.D_5__Parabacteroides	D_0__Bacteria.D_1__Bacteroidetes.D_2__Bacteroidia.D_3__Bacteroidales.D_4__Muribaculaceae.__		D_0__Bacteria.D_1__Firmicutes.D_2__Erysipelotrichia.D_3__Erysipelotrichales.D_4__Erysipelotrichaceae.D_5__Candidatus.Stoquefichus
D_0__Bacteria.D_1__Firmicutes.D_2__Erysipelotrichia.D_3__Erysipelotrichales.D_4__Erysipelotrichaceae.D_5__Erysipelotrichaceae.UCG.003	D_0__Bacteria.D_1__Bacteroidetes.D_2__Bacteroidia.D_3__Cytophagales.D_4__Spirosomaceae.__	D_0__Bacteria.D_1__Bacteroidetes.D_2__Bacteroidia.D_3__Bacteroidales.D_4__uncultured.Ambiguous_taxa	D_0__Bacteria.D_1__Bacteroidetes.D_2__Bacteroidia.D_3__Bacteroidales.D_4__Paludibacteraceae.D_5__F0058		D_0__Bacteria.D_1__Firmicutes.D_2__Erysipelotrichia.D_3__Erysipelotrichales.D_4__Erysipelotrichaceae.D_5__Holdemanella
D_0__Bacteria.D_1__Firmicutes.D_2__Erysipelotrichia.D_3__Erysipelotrichales.D_4__Erysipelotrichaceae.D_5__Solobacterium	D_0__Bacteria.D_1__Bacteroidetes.D_2__Bacteroidia.D_3__Flavobacteriales.D_4__Crocinitomicaceae.D_5__Fluviicola	D_0__Bacteria.D_1__Bacteroidetes.D_2__Bacteroidia.D_3__Bacteroidales.D_4__uncultured.D_5__uncultured.bacterium	D_0__Bacteria.D_1__Bacteroidetes.D_2__Bacteroidia.D_3__Bacteroidales.D_4__Porphyromonadaceae.D_5__Porphyromonas		D_0__Bacteria.D_1__Firmicutes.D_2__Negativicutes.D_3__Selenomonadales.D_4__Veillonellaceae.D_5__Allisonella
D_0__Bacteria.D_1__Firmicutes.D_2__Erysipelotrichia.D_3__Erysipelotrichales.D_4__Erysipelotrichaceae.D_5__Turicibacter	D_0__Bacteria.D_1__Bacteroidetes.D_2__Bacteroidia.D_3__Flavobacteriales.D_4__Flavobacteriaceae.D_5__Capnocytophaga	D_0__Bacteria.D_1__Bacteroidetes.D_2__Bacteroidia.D_3__Chitinophagales.D_4__Chitinophagaceae.D_5__Chitinophaga	D_0__Bacteria.D_1__Bacteroidetes.D_2__Bacteroidia.D_3__Bacteroidales.D_4__Prevotellaceae.D_5__Alloprevotella		D_0__Bacteria.D_1__Firmicutes.D_2__Negativicutes.D_3__Selenomonadales.D_4__Veillonellaceae.D_5__uncultured

OTUs present among HC	OTUs present among Cases_Day1	OTUs present among Cases_Day15	OTUs present among Cases_Day30	OTUs present among only Cases_Day30_A	OTUs present among only Cases_Day30_B
D_0__Bacteria.D_1__Firmicutes.D_2__Negativicutes.D_3__Selenomonadales.D_4__Veillonellaceae.D_5__Anaeroglobus	D_0__Bacteria.D_1__Bacteroidetes.D_2__Bacteroidia.D_3__Flavobacteriales.D_4__Flavobacteriaceae.D_5__Confluentibacter	D_0__Bacteria.D_1__Bacteroidetes.D_2__Bacteroidia.D_3__Chitinophagales.D_4__Chitinophagaceae.D_5__Niabella	D_0__Bacteria.D_1__Bacteroidetes.D_2__Bacteroidia.D_3__Bacteroidales.D_4__Prevotellaceae.D_5__Paraprevotella		D_0__Bacteria.D_1__Lentisphaerae.D_2__Lentisphaeria.D_3__Victivallales.D_4__vadinBE97.D_5__uncultured.bacterium
D_0__Bacteria.D_1__Firmicutes.D_2__Negativicutes.D_3__Selenomonadales.D_4__Veillonellaceae.D_5__Anaerovibrio	D_0__Bacteria.D_1__Bacteroidetes.D_2__Bacteroidia.D_3__Flavobacteriales.D_4__Flavobacteriaceae.D_5__Flavobacterium	D_0__Bacteria.D_1__Bacteroidetes.D_2__Bacteroidia.D_3__Chitinophagales.D_4__Chitinophagaceae.D_5__Sediminibacterium	D_0__Bacteria.D_1__Bacteroidetes.D_2__Bacteroidia.D_3__Bacteroidales.D_4__Prevotellaceae.D_5__Prevotella		D_0__Bacteria.D_1__Proteobacteria.D_2__Alphaproteobacteria.D_3__Caulobacterales.D_4__Caulobacteraceae.D_5__Asticcacaulis
D_0__Bacteria.D_1__Firmicutes.D_2__Negativicutes.D_3__Selenomonadales.D_4__Veillonellaceae.D_5__Selenomonas	D_0__Bacteria.D_1__Bacteroidetes.D_2__Bacteroidia.D_3__Flavobacteriales.D_4__Flavobacteriaceae.__	D_0__Bacteria.D_1__Bacteroidetes.D_2__Bacteroidia.D_3__Chitinophagales.D_4__Chitinophagaceae.D_5__Terrimonas	D_0__Bacteria.D_1__Bacteroidetes.D_2__Bacteroidia.D_3__Bacteroidales.D_4__Prevotellaceae.D_5__Prevotella.2		D_0__Bacteria.D_1__Proteobacteria.D_2__Alphaproteobacteria.D_3__Caulobacterales.D_4__Caulobacteraceae.D_5__Phenylobacterium
D_0__Bacteria.D_1__Firmicutes.D_2__Negativicutes.D_3__Selenomonadales.D_4__Veillonellaceae.D_5__Selenomonas.3	D_0__Bacteria.D_1__Bacteroidetes.D_2__Bacteroidia.D_3__Flavobacteriales.D_4__Weeksellaceae.D_5__Chryseobacterium	D_0__Bacteria.D_1__Bacteroidetes.D_2__Bacteroidia.D_3__Chitinophagales.D_4__Chitinophagaceae.D_5__uncultured	D_0__Bacteria.D_1__Bacteroidetes.D_2__Bacteroidia.D_3__Bacteroidales.D_4__Prevotellaceae.D_5__Prevotella.6		D_0__Bacteria.D_1__Proteobacteria.D_2__Alphaproteobacteria.D_3__Paracaedibacterales.D_4__Paracaedibacteraceae.D_5__Candidatus.Finniella
D_0__Bacteria.D_1__Firmicutes.D_2__Negativicutes.D_3__Selenomonadales.D_4__Veillonellaceae.D_5__uncultured	D_0__Bacteria.D_1__Bacteroidetes.D_2__Bacteroidia.D_3__Flavobacteriales.D_4__Weeksellaceae.D_5__Elizabethkingia	D_0__Bacteria.D_1__Bacteroidetes.D_2__Bacteroidia.D_3__Cytophagales.D_4__Cyclobacteriaceae.D_5__Algoriphagus	D_0__Bacteria.D_1__Bacteroidetes.D_2__Bacteroidia.D_3__Bacteroidales.D_4__Prevotellaceae.D_5__Prevotella.7		D_0__Bacteria.D_1__Proteobacteria.D_2__Alphaproteobacteria.D_3__Paracaedibacterales.D_4__Paracaedibacteraceae.D_5__uncultured
D_0__Bacteria.D_1__Firmicutes.__.__.__.__	D_0__Bacteria.D_1__Bacteroidetes.D_2__Bacteroidia.D_3__Flavobacteriales.D_4__Weeksellaceae.D_5__Empedobacter	D_0__Bacteria.D_1__Bacteroidetes.D_2__Bacteroidia.D_3__Cytophagales.D_4__Microscillaceae.D_5__Siphonobacter	D_0__Bacteria.D_1__Bacteroidetes.D_2__Bacteroidia.D_3__Bacteroidales.D_4__Prevotellaceae.D_5__Prevotella.9		D_0__Bacteria.D_1__Proteobacteria.D_2__Alphaproteobacteria.D_3__Reyranellales.D_4__Reyranellaceae.D_5__Reyranella
D_0__Bacteria.D_1__Fusobacteria.D_2__Fusobacteriia.D_3__Fusobacteriales.D_4__Fusobacteriaceae.D_5__Fusobacterium	D_0__Bacteria.D_1__Bacteroidetes.D_2__Bacteroidia.D_3__Flavobacteriales.D_4__Weeksellaceae.D_5__uncultured	D_0__Bacteria.D_1__Bacteroidetes.D_2__Bacteroidia.D_3__Cytophagales.D_4__Spirosomaceae.D_5__Dyadobacter	D_0__Bacteria.D_1__Bacteroidetes.D_2__Bacteroidia.D_3__Bacteroidales.D_4__Prevotellaceae.D_5__Prevotellaceae.NK3B31.group		D_0__Bacteria.D_1__Proteobacteria.D_2__Alphaproteobacteria.D_3__Rhizobiales.D_4__Rhizobiaceae.D_5__Ensifer
D_0__Bacteria.D_1__Fusobacteria.D_2__Fusobacteriia.D_3__Fusobacteriales.D_4__Leptotrichiaceae.D_5__Leptotrichia	D_0__Bacteria.D_1__Bacteroidetes.D_2__Bacteroidia.D_3__Flavobacteriales.D_4__Weeksellaceae.__	D_0__Bacteria.D_1__Bacteroidetes.D_2__Bacteroidia.D_3__Cytophagales.D_4__Spirosomaceae.D_5__Fibrisoma	D_0__Bacteria.D_1__Bacteroidetes.D_2__Bacteroidia.D_3__Bacteroidales.D_4__Prevotellaceae.D_5__Prevotellaceae.UCG.001		D_0__Bacteria.D_1__Proteobacteria.D_2__Alphaproteobacteria.D_3__Rhizobiales.D_4__Rhizobiaceae.D_5__uncultured
D_0__Bacteria.D_1__Fusobacteria.D_2__Fusobacteriia.D_3__Fusobacteriales.D_4__Leptotrichiaceae.D_5__Streptobacillus	D_0__Bacteria.D_1__Bacteroidetes.D_2__Bacteroidia.D_3__Sphingobacteriales.D_4__Sphingobacteriaceae.D_5__Pedobacter	D_0__Bacteria.D_1__Bacteroidetes.D_2__Bacteroidia.D_3__Cytophagales.D_4__Spirosomaceae.D_5__Flectobacillus	D_0__Bacteria.D_1__Bacteroidetes.D_2__Bacteroidia.D_3__Bacteroidales.D_4__Prevotellaceae.D_5__Prevotellaceae.UCG.003		D_0__Bacteria.D_1__Proteobacteria.D_2__Alphaproteobacteria.D_3__Rhizobiales.D_4__Rhizobiaceae.__
D_0__Bacteria.D_1__Fusobacteria.D_2__Fusobacteriia.D_3__Fusobacteriales.D_4__Leptotrichiaceae.D_5__uncultured	D_0__Bacteria.D_1__Bacteroidetes.D_2__Bacteroidia.D_3__Sphingobacteriales.D_4__Sphingobacteriaceae.D_5__Sphingobacterium	D_0__Bacteria.D_1__Bacteroidetes.D_2__Bacteroidia.D_3__Cytophagales.D_4__Spirosomaceae.D_5__Leadbetterella	D_0__Bacteria.D_1__Bacteroidetes.D_2__Bacteroidia.D_3__Bacteroidales.D_4__Prevotellaceae.D_5__Prevotellaceae.UCG.004		D_0__Bacteria.D_1__Proteobacteria.D_2__Alphaproteobacteria.D_3__Rhodobacterales.D_4__Rhodobacteraceae.D_5__Defluviimonas
D_0__Bacteria.D_1__Patescibacteria.D_2__Gracilibacteria.Ambiguous_taxa.Ambiguous_taxa.Ambiguous_taxa	D_0__Bacteria.D_1__Cyanobacteria.D_2__Melainabacteria.D_3__Gastranaerophilales.D_4__uncultured.bacterium.D_5__uncultured.bacterium	D_0__Bacteria.D_1__Bacteroidetes.D_2__Bacteroidia.D_3__Flavobacteriales.D_4__Crocinitomicaceae.D_5__Fluviicola	D_0__Bacteria.D_1__Bacteroidetes.D_2__Bacteroidia.D_3__Bacteroidales.D_4__Prevotellaceae.D_5__uncultured		D_0__Bacteria.D_1__Proteobacteria.D_2__Alphaproteobacteria.D_3__Rhodobacterales.D_4__Rhodobacteraceae.D_5__Paracoccus
D_0__Bacteria.D_1__Patescibacteria.D_2__Gracilibacteria.D_3__Absconditabacteriales..SR1..D_4__SR1.bacterium.oral.taxon.875.D_5__SR1.bact	D_0__Bacteria.D_1__Cyanobacteria.D_2__Melainabacteria.D_3__Gastranaerophilales.__.__	D_0__Bacteria.D_1__Bacteroidetes.D_2__Bacteroidia.D_3__Flavobacteriales.D_4__Flavobacteriaceae.D_5__Capnocytophaga	D_0__Bacteria.D_1__Bacteroidetes.D_2__Bacteroidia.D_3__Bacteroidales.D_4__Rikenellaceae.D_5__Alistipes		D_0__Bacteria.D_1__Proteobacteria.D_2__Alphaproteobacteria.D_3__Rhodospirillales.D_4__Rhodospirillaceae.D_5__Novispirillum
D_0__Bacteria.D_1__Patescibacteria.D_2__Gracilibacteria.D_3__Absconditabacteriales..SR1..D_4__candidate.division.SR1.bacterium.MGEHA.D	D_0__Bacteria.D_1__Elusimicrobia.D_2__Elusimicrobia.D_3__Elusimicrobiales.D_4__Elusimicrobiaceae.D_5__Elusimicrobium	D_0__Bacteria.D_1__Bacteroidetes.D_2__Bacteroidia.D_3__Flavobacteriales.D_4__Flavobacteriaceae.D_5__Confluentibacter	D_0__Bacteria.D_1__Bacteroidetes.D_2__Bacteroidia.D_3__Bacteroidales.D_4__Rikenellaceae.D_5__Rikenella		D_0__Bacteria.D_1__Proteobacteria.D_2__Alphaproteobacteria.D_3__Rhodospirillales.D_4__uncultured.D_5__uncultured.bacterium
D_0__Bacteria.D_1__Patescibacteria.D_2__Gracilibacteria.D_3__Absconditabacteriales..SR1..D_4__candidate.division.SR1.bacterium.taxon.345.D	D_0__Bacteria.D_1__Epsilonbacteraeota.D_2__Campylobacteria.D_3__Campylobacterales.D_4__Campylobacteraceae.D_5__Campylobacter	D_0__Bacteria.D_1__Bacteroidetes.D_2__Bacteroidia.D_3__Flavobacteriales.D_4__Flavobacteriaceae.D_5__Flavobacterium	D_0__Bacteria.D_1__Bacteroidetes.D_2__Bacteroidia.D_3__Bacteroidales.D_4__Rikenellaceae.D_5__Rikenellaceae.RC9.gut.group		D_0__Bacteria.D_1__Proteobacteria.D_2__Alphaproteobacteria.D_3__Rickettsiales.D_4__Rickettsiaceae.D_5__Rickettsia
D_0__Bacteria.D_1__Patescibacteria.D_2__Saccharimonadia.D_3__Saccharimonadales.D_4__Candidatus.Saccharibacteria.bacterium.UB2523.D_5	D_0__Bacteria.D_1__Firmicutes.D_2__Bacilli.D_3__Bacillales.D_4__Bacillaceae.D_5__Bacillus	D_0__Bacteria.D_1__Bacteroidetes.D_2__Bacteroidia.D_3__Flavobacteriales.D_4__Weeksellaceae.D_5__Chryseobacterium	D_0__Bacteria.D_1__Bacteroidetes.D_2__Bacteroidia.D_3__Bacteroidales.D_4__Tannerellaceae.D_5__Parabacteroides		D_0__Bacteria.D_1__Proteobacteria.D_2__Alphaproteobacteria.D_3__Sphingomonadales.D_4__Sphingomonadaceae.D_5__Blastomonas
D_0__Bacteria.D_1__Patescibacteria.D_2__Saccharimonadia.D_3__Saccharimonadales.D_4__Saccharimonadaceae.D_5__Candidatus.Saccharimon	D_0__Bacteria.D_1__Firmicutes.D_2__Bacilli.D_3__Bacillales.D_4__Family.XI.D_5__Gemella	D_0__Bacteria.D_1__Bacteroidetes.D_2__Bacteroidia.D_3__Flavobacteriales.D_4__Weeksellaceae.D_5__Elizabethkingia	D_0__Bacteria.D_1__Bacteroidetes.D_2__Bacteroidia.D_3__Bacteroidales.D_4__p.2534.18B5.gut.group.D_5__uncultured.bacterium		D_0__Bacteria.D_1__Proteobacteria.D_2__Alphaproteobacteria.__.__.__
D_0__Bacteria.D_1__Patescibacteria.D_2__Saccharimonadia.D_3__Saccharimonadales.D_4__Saccharimonadaceae.D_5__uncultured.bacterium	D_0__Bacteria.D_1__Firmicutes.D_2__Bacilli.D_3__Bacillales.D_4__Family.XII.D_5__Exiguobacterium	D_0__Bacteria.D_1__Bacteroidetes.D_2__Bacteroidia.D_3__Flavobacteriales.D_4__Weeksellaceae.D_5__uncultured	D_0__Bacteria.D_1__Bacteroidetes.D_2__Bacteroidia.D_3__Bacteroidales.D_4__uncultured.D_5__uncultured.bacterium		D_0__Bacteria.D_1__Proteobacteria.D_2__Gammaproteobacteria.D_3__Betaproteobacteriales.D_4__Burkholderiaceae.D_5__Burkholderia.Caballeronia.Paraburkholderia
D_0__Bacteria.D_1__Patescibacteria.D_2__Saccharimonadia.D_3__Saccharimonadales.D_4__Saccharimonadaceae.__	D_0__Bacteria.D_1__Firmicutes.D_2__Bacilli.D_3__Bacillales.D_4__Planococcaceae.D_5__Lysinibacillus	D_0__Bacteria.D_1__Bacteroidetes.D_2__Bacteroidia.D_3__Flavobacteriales.D_4__Weeksellaceae.__	D_0__Bacteria.D_1__Bacteroidetes.D_2__Bacteroidia.D_3__Chitinophagales.D_4__Chitinophagaceae.D_5__Chitinophaga		D_0__Bacteria.D_1__Proteobacteria.D_2__Gammaproteobacteria.D_3__Betaproteobacteriales.D_4__Burkholderiaceae.D_5__Diaphorobacter
D_0__Bacteria.D_1__Patescibacteria.D_2__Saccharimonadia.D_3__Saccharimonadales.D_4__TM7.phylum.sp..oral.clone.FR058.D_5__TM7.phylu	D_0__Bacteria.D_1__Firmicutes.D_2__Bacilli.D_3__Bacillales.D_4__Planococcaceae.D_5__Solibacillus	D_0__Bacteria.D_1__Bacteroidetes.D_2__Bacteroidia.D_3__Flavobacteriales.__.__	D_0__Bacteria.D_1__Bacteroidetes.D_2__Bacteroidia.D_3__Chitinophagales.D_4__Chitinophagaceae.D_5__Niabella		D_0__Bacteria.D_1__Proteobacteria.D_2__Gammaproteobacteria.D_3__Betaproteobacteriales.D_4__Burkholderiaceae.D_5__Herminiimonas
D_0__Bacteria.D_1__Proteobacteria.D_2__Alphaproteobacteria.D_3__Acetobacterales.D_4__Acetobacteraceae.D_5__Roseomonas	D_0__Bacteria.D_1__Firmicutes.D_2__Bacilli.D_3__Bacillales.D_4__Planococcaceae.D_5__uncultured	D_0__Bacteria.D_1__Bacteroidetes.D_2__Bacteroidia.D_3__Sphingobacteriales.D_4__Sphingobacteriaceae.D_5__Mucilaginibacter	D_0__Bacteria.D_1__Bacteroidetes.D_2__Bacteroidia.D_3__Chitinophagales.D_4__Chitinophagaceae.D_5__Sediminibacterium		D_0__Bacteria.D_1__Proteobacteria.D_2__Gammaproteobacteria.D_3__Betaproteobacteriales.D_4__Burkholderiaceae.D_5__Lautropia
D_0__Bacteria.D_1__Proteobacteria.D_2__Alphaproteobacteria.D_3__Azospirillales.D_4__Azospirillaceae.D_5__Azospirillum	D_0__Bacteria.D_1__Firmicutes.D_2__Bacilli.D_3__Bacillales.D_4__Planococcaceae.__	D_0__Bacteria.D_1__Bacteroidetes.D_2__Bacteroidia.D_3__Sphingobacteriales.D_4__Sphingobacteriaceae.D_5__Pedobacter	D_0__Bacteria.D_1__Bacteroidetes.D_2__Bacteroidia.D_3__Chitinophagales.D_4__Chitinophagaceae.D_5__Taibaiella		D_0__Bacteria.D_1__Proteobacteria.D_2__Gammaproteobacteria.D_3__Betaproteobacteriales.D_4__Burkholderiaceae.D_5__Pelomonas
D_0__Bacteria.D_1__Proteobacteria.D_2__Alphaproteobacteria.D_3__Rhodospirillales.D_4__uncultured.D_5__uncultured.bacterium	D_0__Bacteria.D_1__Firmicutes.D_2__Bacilli.D_3__Bacillales.D_4__Staphylococcaceae.D_5__Salinicoccus	D_0__Bacteria.D_1__Bacteroidetes.D_2__Bacteroidia.D_3__Sphingobacteriales.D_4__Sphingobacteriaceae.D_5__Sphingobacterium	D_0__Bacteria.D_1__Bacteroidetes.D_2__Bacteroidia.D_3__Chitinophagales.D_4__Chitinophagaceae.D_5__Terrimonas		D_0__Bacteria.D_1__Proteobacteria.D_2__Gammaproteobacteria.D_3__Betaproteobacteriales.D_4__Burkholderiaceae.D_5__Pigmentiphaga
D_0__Bacteria.D_1__Proteobacteria.D_2__Deltaproteobacteria.D_3__Desulfovibrionales.D_4__Desulfovibrionaceae.D_5__Bilophila	D_0__Bacteria.D_1__Firmicutes.D_2__Bacilli.D_3__Bacillales.D_4__Staphylococcaceae.D_5__Staphylococcus	D_0__Bacteria.D_1__Bacteroidetes.D_2__Bacteroidia.D_3__Sphingobacteriales.D_4__Sphingobacteriaceae.__	D_0__Bacteria.D_1__Bacteroidetes.D_2__Bacteroidia.D_3__Chitinophagales.D_4__Chitinophagaceae.D_5__uncultured		D_0__Bacteria.D_1__Proteobacteria.D_2__Gammaproteobacteria.D_3__Betaproteobacteriales.D_4__Burkholderiaceae.D_5__Pseudacidovorax
D_0__Bacteria.D_1__Proteobacteria.D_2__Gammaproteobacteria.D_3__Betaproteobacteriales.D_4__Burkholderiaceae.Ambiguous_taxa	D_0__Bacteria.D_1__Firmicutes.D_2__Bacilli.D_3__Bacillales.__.__	D_0__Bacteria.D_1__Cyanobacteria.D_2__Melainabacteria.D_3__Gastranaerophilales.Ambiguous_taxa.Ambiguous_taxa	D_0__Bacteria.D_1__Bacteroidetes.D_2__Bacteroidia.D_3__Cytophagales.D_4__Cyclobacteriaceae.D_5__Algoriphagus		D_0__Bacteria.D_1__Proteobacteria.D_2__Gammaproteobacteria.D_3__Betaproteobacteriales.D_4__Burkholderiaceae.D_5__Ramlibacter
D_0__Bacteria.D_1__Proteobacteria.D_2__Gammaproteobacteria.D_3__Betaproteobacteriales.D_4__Burkholderiaceae.D_5__Herminiimonas	D_0__Bacteria.D_1__Firmicutes.D_2__Bacilli.D_3__Lactobacillales.D_4__Aerococcaceae.D_5__Aerococcus	D_0__Bacteria.D_1__Cyanobacteria.D_2__Melainabacteria.D_3__Gastranaerophilales.D_4__uncultured.bacterium.D_5__uncultured.bacterium	D_0__Bacteria.D_1__Bacteroidetes.D_2__Bacteroidia.D_3__Cytophagales.D_4__Microscillaceae.D_5__Siphonobacter		D_0__Bacteria.D_1__Proteobacteria.D_2__Gammaproteobacteria.D_3__Betaproteobacteriales.D_4__Burkholderiaceae.D_5__Roseateles
D_0__Bacteria.D_1__Proteobacteria.D_2__Gammaproteobacteria.D_3__Betaproteobacteriales.D_4__Burkholderiaceae.D_5__Lautropia	D_0__Bacteria.D_1__Firmicutes.D_2__Bacilli.D_3__Lactobacillales.D_4__Carnobacteriaceae.D_5__Atopostipes	D_0__Bacteria.D_1__Cyanobacteria.D_2__Melainabacteria.D_3__Gastranaerophilales.__.__	D_0__Bacteria.D_1__Bacteroidetes.D_2__Bacteroidia.D_3__Cytophagales.D_4__Spirosomaceae.D_5__Dyadobacter		D_0__Bacteria.D_1__Proteobacteria.D_2__Gammaproteobacteria.D_3__Betaproteobacteriales.D_4__Burkholderiaceae.D_5__Variovorax
D_0__Bacteria.D_1__Proteobacteria.D_2__Gammaproteobacteria.D_3__Betaproteobacteriales.D_4__Neisseriaceae.D_5__Alysiella	D_0__Bacteria.D_1__Firmicutes.D_2__Bacilli.D_3__Lactobacillales.D_4__Carnobacteriaceae.D_5__Granulicatella	D_0__Bacteria.D_1__Cyanobacteria.D_2__Melainabacteria.D_3__Obscuribacterales.Ambiguous_taxa.Ambiguous_taxa	D_0__Bacteria.D_1__Bacteroidetes.D_2__Bacteroidia.D_3__Cytophagales.D_4__Spirosomaceae.D_5__Flectobacillus		D_0__Bacteria.D_1__Proteobacteria.D_2__Gammaproteobacteria.D_3__Betaproteobacteriales.D_4__Burkholderiaceae.D_5__Xenophilus
D_0__Bacteria.D_1__Proteobacteria.D_2__Gammaproteobacteria.D_3__Betaproteobacteriales.D_4__Neisseriaceae.D_5__Eikenella	D_0__Bacteria.D_1__Firmicutes.D_2__Bacilli.D_3__Lactobacillales.D_4__Enterococcaceae.D_5__Enterococcus	D_0__Bacteria.D_1__Elusimicrobia.D_2__Elusimicrobia.D_3__Elusimicrobiales.D_4__Elusimicrobiaceae.D_5__Elusimicrobium	D_0__Bacteria.D_1__Bacteroidetes.D_2__Bacteroidia.D_3__Cytophagales.D_4__Spirosomaceae.D_5__Leadbetterella		D_0__Bacteria.D_1__Proteobacteria.D_2__Gammaproteobacteria.D_3__Betaproteobacteriales.D_4__Chromobacteriaceae.D_5__Chromobacterium
D_0__Bacteria.D_1__Proteobacteria.D_2__Gammaproteobacteria.D_3__Betaproteobacteriales.D_4__Neisseriaceae.D_5__Kingella	D_0__Bacteria.D_1__Firmicutes.D_2__Bacilli.D_3__Lactobacillales.D_4__Enterococcaceae.__	D_0__Bacteria.D_1__Epsilonbacteraeota.D_2__Campylobacteria.D_3__Campylobacterales.D_4__Campylobacteraceae.D_5__Campylobacter	D_0__Bacteria.D_1__Bacteroidetes.D_2__Bacteroidia.D_3__Cytophagales.D_4__Spirosomaceae.D_5__Spirosoma		D_0__Bacteria.D_1__Proteobacteria.D_2__Gammaproteobacteria.D_3__Betaproteobacteriales.D_4__Rhodocyclaceae.D_5__Methyloversatilis
D_0__Bacteria.D_1__Proteobacteria.D_2__Gammaproteobacteria.D_3__Betaproteobacteriales.D_4__Neisseriaceae.__	D_0__Bacteria.D_1__Firmicutes.D_2__Bacilli.D_3__Lactobacillales.D_4__Lactobacillaceae.D_5__Lactobacillus	D_0__Bacteria.D_1__Firmicutes.D_2__Bacilli.D_3__Bacillales.D_4__Bacillaceae.D_5__Bacillus	D_0__Bacteria.D_1__Bacteroidetes.D_2__Bacteroidia.D_3__Flavobacteriales.D_4__Crocinitomicaceae.D_5__Fluviicola		D_0__Bacteria.D_1__Proteobacteria.D_2__Gammaproteobacteria.D_3__Betaproteobacteriales.__.__
D_0__Bacteria.D_1__Proteobacteria.D_2__Gammaproteobacteria.D_3__Betaproteobacteriales.D_4__Rhodocyclaceae.D_5__Propionivibrio	D_0__Bacteria.D_1__Firmicutes.D_2__Bacilli.D_3__Lactobacillales.D_4__Leuconostocaceae.D_5__Weissella	D_0__Bacteria.D_1__Firmicutes.D_2__Bacilli.D_3__Bacillales.D_4__Bacillaceae.__	D_0__Bacteria.D_1__Bacteroidetes.D_2__Bacteroidia.D_3__Flavobacteriales.D_4__Flavobacteriaceae.D_5__Capnocytophaga		D_0__Bacteria.D_1__Proteobacteria.D_2__Gammaproteobacteria.D_3__Enterobacteriales.D_4__Enterobacteriaceae.D_5__Cronobacter
D_0__Bacteria.D_1__Proteobacteria.D_2__Gammaproteobacteria.D_3__Cardiobacteriales.D_4__Cardiobacteriaceae.D_5__Cardiobacterium	D_0__Bacteria.D_1__Firmicutes.D_2__Bacilli.D_3__Lactobacillales.D_4__Streptococcaceae.D_5__Lactococcus	D_0__Bacteria.D_1__Firmicutes.D_2__Bacilli.D_3__Bacillales.D_4__Family.XII.D_5__Exiguobacterium	D_0__Bacteria.D_1__Bacteroidetes.D_2__Bacteroidia.D_3__Flavobacteriales.D_4__Flavobacteriaceae.D_5__Flavobacterium		D_0__Bacteria.D_1__Proteobacteria.D_2__Gammaproteobacteria.D_3__Enterobacteriales.D_4__Enterobacteriaceae.D_5__Rahnella
D_0__Bacteria.D_1__Proteobacteria.D_2__Gammaproteobacteria.D_3__Diplorickettsiales.D_4__Diplorickettsiaceae.D_5__uncultured	D_0__Bacteria.D_1__Firmicutes.D_2__Bacilli.D_3__Lactobacillales.D_4__Streptococcaceae.D_5__Streptococcus	D_0__Bacteria.D_1__Firmicutes.D_2__Bacilli.D_3__Bacillales.D_4__Paenibacillaceae.D_5__Paenibacillus	D_0__Bacteria.D_1__Bacteroidetes.D_2__Bacteroidia.D_3__Flavobacteriales.D_4__Weeksellaceae.D_5__Chryseobacterium		D_0__Bacteria.D_1__Proteobacteria.D_2__Gammaproteobacteria.D_3__Gammaproteobacteria.Incertae.Sedis.D_4__Unknown.Family.D_5__uncultured.bacterium
D_0__Bacteria.D_1__Proteobacteria.D_2__Gammaproteobacteria.D_3__Oceanospirillales.D_4__Pseudohongiellaceae.D_5__Pseudohongiella	D_0__Bacteria.D_1__Firmicutes.D_2__Bacilli.D_3__Lactobacillales.__.__	D_0__Bacteria.D_1__Firmicutes.D_2__Bacilli.D_3__Bacillales.D_4__Planococcaceae.D_5__Caryophanon	D_0__Bacteria.D_1__Bacteroidetes.D_2__Bacteroidia.D_3__Flavobacteriales.D_4__Weeksellaceae.D_5__Elizabethkingia		D_0__Bacteria.D_1__Proteobacteria.D_2__Gammaproteobacteria.D_3__Salinisphaerales.D_4__Solimonadaceae.D_5__uncultured
D_0__Bacteria.D_1__Proteobacteria.D_2__Gammaproteobacteria.D_3__Pasteurellales.D_4__Pasteurellaceae.D_5__Aggregatibacter	D_0__Bacteria.D_1__Firmicutes.D_2__Clostridia.D_3__Clostridiales.D_4__Christensenellaceae.D_5__Christensenellaceae.R.7.group	D_0__Bacteria.D_1__Firmicutes.D_2__Bacilli.D_3__Bacillales.D_4__Planococcaceae.D_5__Lysinibacillus	D_0__Bacteria.D_1__Bacteroidetes.D_2__Bacteroidia.D_3__Flavobacteriales.D_4__Weeksellaceae.D_5__Empedobacter		D_0__Bacteria.D_1__Verrucomicrobia.D_2__Verrucomicrobiae.D_3__Verrucomicrobiales.D_4__Akkermansiaceae.D_5__Akkermansia
D_0__Bacteria.D_1__Proteobacteria.D_2__Gammaproteobacteria.D_3__Pasteurellales.D_4__Pasteurellaceae.__	D_0__Bacteria.D_1__Firmicutes.D_2__Clostridia.D_3__Clostridiales.D_4__Christensenellaceae.__	D_0__Bacteria.D_1__Firmicutes.D_2__Bacilli.D_3__Bacillales.D_4__Planococcaceae.D_5__Solibacillus	D_0__Bacteria.D_1__Bacteroidetes.D_2__Bacteroidia.D_3__Flavobacteriales.D_4__Weeksellaceae.D_5__Moheibacter		D_0__Bacteria.__.__.__.__.__
D_0__Bacteria.D_1__Proteobacteria.D_2__Gammaproteobacteria.D_3__Pseudomonadales.D_4__Moraxellaceae.D_5__Enhydrobacter	D_0__Bacteria.D_1__Firmicutes.D_2__Clostridia.D_3__Clostridiales.D_4__Clostridiaceae.1.D_5__Clostridium.sensu.stricto.1	D_0__Bacteria.D_1__Firmicutes.D_2__Bacilli.D_3__Bacillales.D_4__Planococcaceae.__	D_0__Bacteria.D_1__Bacteroidetes.D_2__Bacteroidia.D_3__Flavobacteriales.D_4__Weeksellaceae.D_5__uncultured		
D_0__Bacteria.D_1__Synergistetes.D_2__Synergistia.D_3__Synergistales.D_4__Synergistaceae.D_5__Fretibacterium	D_0__Bacteria.D_1__Firmicutes.D_2__Clostridia.D_3__Clostridiales.D_4__Clostridiaceae.1.D_5__Clostridium.sensu.stricto.13	D_0__Bacteria.D_1__Firmicutes.D_2__Bacilli.D_3__Bacillales.D_4__Staphylococcaceae.D_5__Staphylococcus	D_0__Bacteria.D_1__Bacteroidetes.D_2__Bacteroidia.D_3__Sphingobacteriales.D_4__Lentimicrobiaceae.D_5__Bacteroidia.bacterium.feline.oral.taxon.312		
D_0__Bacteria.D_1__Tenericutes.D_2__Mollicutes.D_3__Mollicutes.RF39.D_4__uncultured.bacterium.D_5__uncultured.bacterium	D_0__Bacteria.D_1__Firmicutes.D_2__Clostridia.D_3__Clostridiales.D_4__Clostridiaceae.1.D_5__Clostridium.sensu.stricto.3	D_0__Bacteria.D_1__Firmicutes.D_2__Bacilli.D_3__Bacillales.__.__	D_0__Bacteria.D_1__Bacteroidetes.D_2__Bacteroidia.D_3__Sphingobacteriales.D_4__Sphingobacteriaceae.D_5__Nubsella		
D_0__Bacteria.D_1__Verrucomicrobia.D_2__Verrucomicrobiae.D_3__Verrucomicrobiales.D_4__Verrucomicrobiaceae.D_5__Prosthecobacter	D_0__Bacteria.D_1__Firmicutes.D_2__Clostridia.D_3__Clostridiales.D_4__Clostridiaceae.1.D_5__Clostridium.sensu.stricto.8	D_0__Bacteria.D_1__Firmicutes.D_2__Bacilli.D_3__Lactobacillales.D_4__Aerococcaceae.D_5__Aerococcus	D_0__Bacteria.D_1__Bacteroidetes.D_2__Bacteroidia.D_3__Sphingobacteriales.D_4__Sphingobacteriaceae.D_5__Pedobacter		
	D_0__Bacteria.D_1__Firmicutes.D_2__Clostridia.D_3__Clostridiales.D_4__Clostridiaceae.1.D_5__Sarcina	D_0__Bacteria.D_1__Firmicutes.D_2__Bacilli.D_3__Lactobacillales.D_4__Aerococcaceae.D_5__Facklamia	D_0__Bacteria.D_1__Bacteroidetes.D_2__Bacteroidia.D_3__Sphingobacteriales.D_4__Sphingobacteriaceae.D_5__Sphingobacterium		
	D_0__Bacteria.D_1__Firmicutes.D_2__Clostridia.D_3__Clostridiales.D_4__Clostridiaceae.1.__	D_0__Bacteria.D_1__Firmicutes.D_2__Bacilli.D_3__Lactobacillales.D_4__Carnobacteriaceae.D_5__Atopostipes	D_0__Bacteria.D_1__Cyanobacteria.D_2__Melainabacteria.D_3__Gastranaerophilales.D_4__uncultured.bacterium.D_5__uncultured.bacterium		
	D_0__Bacteria.D_1__Firmicutes.D_2__Clostridia.D_3__Clostridiales.D_4__Clostridiales.vadinBB60.group.D_5__gut.metagenome	D_0__Bacteria.D_1__Firmicutes.D_2__Bacilli.D_3__Lactobacillales.D_4__Carnobacteriaceae.D_5__Carnobacterium	D_0__Bacteria.D_1__Cyanobacteria.D_2__Melainabacteria.D_3__Gastranaerophilales.__.__		
	D_0__Bacteria.D_1__Firmicutes.D_2__Clostridia.D_3__Clostridiales.D_4__Clostridiales.vadinBB60.group.D_5__uncultured.bacterium	D_0__Bacteria.D_1__Firmicutes.D_2__Bacilli.D_3__Lactobacillales.D_4__Carnobacteriaceae.D_5__Granulicatella	D_0__Bacteria.D_1__Cyanobacteria.D_2__Melainabacteria.D_3__Obscuribacterales.__.__		
	D_0__Bacteria.D_1__Firmicutes.D_2__Clostridia.D_3__Clostridiales.D_4__Eubacteriaceae.D_5__Eubacterium	D_0__Bacteria.D_1__Firmicutes.D_2__Bacilli.D_3__Lactobacillales.D_4__Enterococcaceae.D_5__Enterococcus	D_0__Bacteria.D_1__Cyanobacteria.D_2__Oxyphotobacteria.D_3__Chloroplast.__.__		
	D_0__Bacteria.D_1__Firmicutes.D_2__Clostridia.D_3__Clostridiales.D_4__Family.XI.D_5__Anaerococcus	D_0__Bacteria.D_1__Firmicutes.D_2__Bacilli.D_3__Lactobacillales.D_4__Enterococcaceae.__	D_0__Bacteria.D_1__Elusimicrobia.D_2__Elusimicrobia.D_3__Elusimicrobiales.D_4__Elusimicrobiaceae.D_5__Elusimicrobium		
	D_0__Bacteria.D_1__Firmicutes.D_2__Clostridia.D_3__Clostridiales.D_4__Family.XI.D_5__Ezakiella	D_0__Bacteria.D_1__Firmicutes.D_2__Bacilli.D_3__Lactobacillales.D_4__Lactobacillaceae.D_5__Lactobacillus	D_0__Bacteria.D_1__Epsilonbacteraeota.D_2__Campylobacteria.D_3__Campylobacterales.D_4__Campylobacteraceae.D_5__Campylobacter		
	D_0__Bacteria.D_1__Firmicutes.D_2__Clostridia.D_3__Clostridiales.D_4__Family.XI.D_5__Finegoldia	D_0__Bacteria.D_1__Firmicutes.D_2__Bacilli.D_3__Lactobacillales.D_4__Leuconostocaceae.D_5__Weissella	D_0__Bacteria.D_1__Epsilonbacteraeota.D_2__Campylobacteria.D_3__Campylobacterales.D_4__Helicobacteraceae.D_5__Helicobacter		
	D_0__Bacteria.D_1__Firmicutes.D_2__Clostridia.D_3__Clostridiales.D_4__Family.XI.D_5__Parvimonas	D_0__Bacteria.D_1__Firmicutes.D_2__Bacilli.D_3__Lactobacillales.D_4__P5D1.392.D_5__Granulicatella.sp..BB.11	D_0__Bacteria.D_1__Firmicutes.D_2__Bacilli.D_3__Bacillales.D_4__Bacillaceae.D_5__Bacillus		
	D_0__Bacteria.D_1__Firmicutes.D_2__Clostridia.D_3__Clostridiales.D_4__Family.XI.D_5__Peptoniphilus	D_0__Bacteria.D_1__Firmicutes.D_2__Bacilli.D_3__Lactobacillales.D_4__P5D1.392.D_5__Streptococcus.sp..oral.clone.ASCB12	D_0__Bacteria.D_1__Firmicutes.D_2__Bacilli.D_3__Bacillales.D_4__Family.XI.D_5__Gemella		
	D_0__Bacteria.D_1__Firmicutes.D_2__Clostridia.D_3__Clostridiales.D_4__Family.XI.D_5__W5053	D_0__Bacteria.D_1__Firmicutes.D_2__Bacilli.D_3__Lactobacillales.D_4__Streptococcaceae.D_5__Lactococcus	D_0__Bacteria.D_1__Firmicutes.D_2__Bacilli.D_3__Bacillales.D_4__Family.XII.D_5__Exiguobacterium		
	D_0__Bacteria.D_1__Firmicutes.D_2__Clostridia.D_3__Clostridiales.D_4__Family.XIII.D_5__Family.XIII.AD3011.group	D_0__Bacteria.D_1__Firmicutes.D_2__Bacilli.D_3__Lactobacillales.D_4__Streptococcaceae.D_5__Streptococcus	D_0__Bacteria.D_1__Firmicutes.D_2__Bacilli.D_3__Bacillales.D_4__Paenibacillaceae.D_5__Paenibacillus		
	D_0__Bacteria.D_1__Firmicutes.D_2__Clostridia.D_3__Clostridiales.D_4__Family.XIII.D_5__S5.A14a	D_0__Bacteria.D_1__Firmicutes.D_2__Bacilli.D_3__Lactobacillales.__.__	D_0__Bacteria.D_1__Firmicutes.D_2__Bacilli.D_3__Bacillales.D_4__Paenibacillaceae.__		
	D_0__Bacteria.D_1__Firmicutes.D_2__Clostridia.D_3__Clostridiales.D_4__Lachnospiraceae.Ambiguous_taxa	D_0__Bacteria.D_1__Firmicutes.D_2__Clostridia.D_3__Clostridiales.D_4__Christensenellaceae.D_5__Christensenellaceae.R.7.group	D_0__Bacteria.D_1__Firmicutes.D_2__Bacilli.D_3__Bacillales.D_4__Planococcaceae.D_5__Lysinibacillus		
	D_0__Bacteria.D_1__Firmicutes.D_2__Clostridia.D_3__Clostridiales.D_4__Lachnospiraceae.D_5__Agathobacter	D_0__Bacteria.D_1__Firmicutes.D_2__Clostridia.D_3__Clostridiales.D_4__Clostridiaceae.1.D_5__Clostridium.sensu.stricto.1	D_0__Bacteria.D_1__Firmicutes.D_2__Bacilli.D_3__Bacillales.D_4__Planococcaceae.D_5__Solibacillus		
	D_0__Bacteria.D_1__Firmicutes.D_2__Clostridia.D_3__Clostridiales.D_4__Lachnospiraceae.D_5__Anaerocolumna	D_0__Bacteria.D_1__Firmicutes.D_2__Clostridia.D_3__Clostridiales.D_4__Clostridiaceae.1.D_5__Clostridium.sensu.stricto.13	D_0__Bacteria.D_1__Firmicutes.D_2__Bacilli.D_3__Bacillales.D_4__Planococcaceae.__		
	D_0__Bacteria.D_1__Firmicutes.D_2__Clostridia.D_3__Clostridiales.D_4__Lachnospiraceae.D_5__Anaerostipes	D_0__Bacteria.D_1__Firmicutes.D_2__Clostridia.D_3__Clostridiales.D_4__Clostridiaceae.1.D_5__Clostridium.sensu.stricto.3	D_0__Bacteria.D_1__Firmicutes.D_2__Bacilli.D_3__Bacillales.D_4__Staphylococcaceae.D_5__Jeotgalicoccus		

OTUs present among HC	OTUs present among Cases_Day1	OTUs present among Cases_Day15	OTUs present among Cases_Day30	OTUs present among only Cases_Day30_A	OTUs present among only Cases_Day30_B
	D_0__Bacteria.D_1__Firmicutes.D_2__Clostridia.D_3__Clostridiales.D_4__Lachnospiraceae.D_5__Blautia	D_0__Bacteria.D_1__Firmicutes.D_2__Clostridia.D_3__Clostridiales.D_4__Clostridiaceae.1.D_5__Clostridium.sensu.stricto.6	D_0__Bacteria.D_1__Firmicutes.D_2__Bacilli.D_3__Bacillales.D_4__Staphylococcaceae.D_5__Nosocomiicoccus		
	D_0__Bacteria.D_1__Firmicutes.D_2__Clostridia.D_3__Clostridiales.D_4__Lachnospiraceae.D_5__Butyrivibrio	D_0__Bacteria.D_1__Firmicutes.D_2__Clostridia.D_3__Clostridiales.D_4__Clostridiaceae.1.D_5__Sarcina	D_0__Bacteria.D_1__Firmicutes.D_2__Bacilli.D_3__Bacillales.D_4__Staphylococcaceae.D_5__Staphylococcus		
	D_0__Bacteria.D_1__Firmicutes.D_2__Clostridia.D_3__Clostridiales.D_4__Lachnospiraceae.D_5__Cellulosilyticum	D_0__Bacteria.D_1__Firmicutes.D_2__Clostridia.D_3__Clostridiales.D_4__Clostridiales.vadinBB60.group.Ambiguous_taxa	D_0__Bacteria.D_1__Firmicutes.D_2__Bacilli.D_3__Bacillales.__.__		
	D_0__Bacteria.D_1__Firmicutes.D_2__Clostridia.D_3__Clostridiales.D_4__Lachnospiraceae.D_5__Coprococcus.1	D_0__Bacteria.D_1__Firmicutes.D_2__Clostridia.D_3__Clostridiales.D_4__Clostridiales.vadinBB60.group.D_5__gut.metagenome	D_0__Bacteria.D_1__Firmicutes.D_2__Bacilli.D_3__Lactobacillales.D_4__Aerococcaceae.D_5__Aerococcus		
	D_0__Bacteria.D_1__Firmicutes.D_2__Clostridia.D_3__Clostridiales.D_4__Lachnospiraceae.D_5__Coprococcus.2	D_0__Bacteria.D_1__Firmicutes.D_2__Clostridia.D_3__Clostridiales.D_4__Family.XI.D_5__Anaerococcus	D_0__Bacteria.D_1__Firmicutes.D_2__Bacilli.D_3__Lactobacillales.D_4__Aerococcaceae.D_5__Facklamia		
	D_0__Bacteria.D_1__Firmicutes.D_2__Clostridia.D_3__Clostridiales.D_4__Lachnospiraceae.D_5__Coprococcus.3	D_0__Bacteria.D_1__Firmicutes.D_2__Clostridia.D_3__Clostridiales.D_4__Family.XI.D_5__Ezakiella	D_0__Bacteria.D_1__Firmicutes.D_2__Bacilli.D_3__Lactobacillales.D_4__Carnobacteriaceae.D_5__Atopostipes		
	D_0__Bacteria.D_1__Firmicutes.D_2__Clostridia.D_3__Clostridiales.D_4__Lachnospiraceae.D_5__Dorea	D_0__Bacteria.D_1__Firmicutes.D_2__Clostridia.D_3__Clostridiales.D_4__Family.XI.D_5__Finegoldia	D_0__Bacteria.D_1__Firmicutes.D_2__Bacilli.D_3__Lactobacillales.D_4__Carnobacteriaceae.D_5__Granulicatella		
	D_0__Bacteria.D_1__Firmicutes.D_2__Clostridia.D_3__Clostridiales.D_4__Lachnospiraceae.D_5__Epulopiscium	D_0__Bacteria.D_1__Firmicutes.D_2__Clostridia.D_3__Clostridiales.D_4__Family.XI.D_5__Parvimonas	D_0__Bacteria.D_1__Firmicutes.D_2__Bacilli.D_3__Lactobacillales.D_4__Enterococcaceae.D_5__Enterococcus		
	D_0__Bacteria.D_1__Firmicutes.D_2__Clostridia.D_3__Clostridiales.D_4__Lachnospiraceae.D_5__Fusicatenibacter	D_0__Bacteria.D_1__Firmicutes.D_2__Clostridia.D_3__Clostridiales.D_4__Family.XI.D_5__Peptoniphilus	D_0__Bacteria.D_1__Firmicutes.D_2__Bacilli.D_3__Lactobacillales.D_4__Lactobacillaceae.D_5__Lactobacillus		
	D_0__Bacteria.D_1__Firmicutes.D_2__Clostridia.D_3__Clostridiales.D_4__Lachnospiraceae.D_5__Howardella	D_0__Bacteria.D_1__Firmicutes.D_2__Clostridia.D_3__Clostridiales.D_4__Family.XI.D_5__W5053	D_0__Bacteria.D_1__Firmicutes.D_2__Bacilli.D_3__Lactobacillales.D_4__Leuconostocaceae.D_5__Leuconostoc		
	D_0__Bacteria.D_1__Firmicutes.D_2__Clostridia.D_3__Clostridiales.D_4__Lachnospiraceae.D_5__Hungatella	D_0__Bacteria.D_1__Firmicutes.D_2__Clostridia.D_3__Clostridiales.D_4__Family.XIII.D_5__Anaerovorax	D_0__Bacteria.D_1__Firmicutes.D_2__Bacilli.D_3__Lactobacillales.D_4__Leuconostocaceae.D_5__Weissella		
	D_0__Bacteria.D_1__Firmicutes.D_2__Clostridia.D_3__Clostridiales.D_4__Lachnospiraceae.D_5__Lachnoanaerobaculum	D_0__Bacteria.D_1__Firmicutes.D_2__Clostridia.D_3__Clostridiales.D_4__Family.XIII.D_5__Family.XIII.AD3011.group	D_0__Bacteria.D_1__Firmicutes.D_2__Bacilli.D_3__Lactobacillales.D_4__Streptococcaceae.D_5__Lactococcus		
	D_0__Bacteria.D_1__Firmicutes.D_2__Clostridia.D_3__Clostridiales.D_4__Lachnospiraceae.D_5__Lachnoclostridium.10	D_0__Bacteria.D_1__Firmicutes.D_2__Clostridia.D_3__Clostridiales.D_4__Family.XIII.D_5__S5.A14a	D_0__Bacteria.D_1__Firmicutes.D_2__Bacilli.D_3__Lactobacillales.D_4__Streptococcaceae.D_5__Streptococcus		
	D_0__Bacteria.D_1__Firmicutes.D_2__Clostridia.D_3__Clostridiales.D_4__Lachnospiraceae.D_5__Lachnospira	D_0__Bacteria.D_1__Firmicutes.D_2__Clostridia.D_3__Clostridiales.D_4__Family.XIII.__	D_0__Bacteria.D_1__Firmicutes.D_2__Bacilli.D_3__Lactobacillales.__.__		
	D_0__Bacteria.D_1__Firmicutes.D_2__Clostridia.D_3__Clostridiales.D_4__Lachnospiraceae.D_5__Lachnospiraceae.AC2044.group	D_0__Bacteria.D_1__Firmicutes.D_2__Clostridia.D_3__Clostridiales.D_4__Lachnospiraceae.Ambiguous_taxa	D_0__Bacteria.D_1__Firmicutes.D_2__Clostridia.D_3__Clostridiales.D_4__Christensenellaceae.D_5__Christensenellaceae.R.7.group		
	D_0__Bacteria.D_1__Firmicutes.D_2__Clostridia.D_3__Clostridiales.D_4__Lachnospiraceae.D_5__Lachnospiraceae.NC2004.group	D_0__Bacteria.D_1__Firmicutes.D_2__Clostridia.D_3__Clostridiales.D_4__Lachnospiraceae.D_5__Agathobacter	D_0__Bacteria.D_1__Firmicutes.D_2__Clostridia.D_3__Clostridiales.D_4__Christensenellaceae.__		
	D_0__Bacteria.D_1__Firmicutes.D_2__Clostridia.D_3__Clostridiales.D_4__Lachnospiraceae.D_5__Lachnospiraceae.ND3007.group	D_0__Bacteria.D_1__Firmicutes.D_2__Clostridia.D_3__Clostridiales.D_4__Lachnospiraceae.D_5__Anaerocolumna	D_0__Bacteria.D_1__Firmicutes.D_2__Clostridia.D_3__Clostridiales.D_4__Clostridiaceae.1.D_5__Candidatus.Arthromitus		
	D_0__Bacteria.D_1__Firmicutes.D_2__Clostridia.D_3__Clostridiales.D_4__Lachnospiraceae.D_5__Lachnospiraceae.NK3A20.group	D_0__Bacteria.D_1__Firmicutes.D_2__Clostridia.D_3__Clostridiales.D_4__Lachnospiraceae.D_5__Anaerostipes	D_0__Bacteria.D_1__Firmicutes.D_2__Clostridia.D_3__Clostridiales.D_4__Clostridiaceae.1.D_5__Clostridium.sensu.stricto.1		
	D_0__Bacteria.D_1__Firmicutes.D_2__Clostridia.D_3__Clostridiales.D_4__Lachnospiraceae.D_5__Lachnospiraceae.NK4A136.group	D_0__Bacteria.D_1__Firmicutes.D_2__Clostridia.D_3__Clostridiales.D_4__Lachnospiraceae.D_5__Blautia	D_0__Bacteria.D_1__Firmicutes.D_2__Clostridia.D_3__Clostridiales.D_4__Clostridiaceae.1.D_5__Clostridium.sensu.stricto.13		
	D_0__Bacteria.D_1__Firmicutes.D_2__Clostridia.D_3__Clostridiales.D_4__Lachnospiraceae.D_5__Marvinbryantia	D_0__Bacteria.D_1__Firmicutes.D_2__Clostridia.D_3__Clostridiales.D_4__Lachnospiraceae.D_5__Butyrivibrio	D_0__Bacteria.D_1__Firmicutes.D_2__Clostridia.D_3__Clostridiales.D_4__Clostridiaceae.1.D_5__Clostridium.sensu.stricto.3		
	D_0__Bacteria.D_1__Firmicutes.D_2__Clostridia.D_3__Clostridiales.D_4__Lachnospiraceae.D_5__Moryella	D_0__Bacteria.D_1__Firmicutes.D_2__Clostridia.D_3__Clostridiales.D_4__Lachnospiraceae.D_5__CAG.56	D_0__Bacteria.D_1__Firmicutes.D_2__Clostridia.D_3__Clostridiales.D_4__Clostridiales.vadinBB60.group.D_5__gut.metagenome		
	D_0__Bacteria.D_1__Firmicutes.D_2__Clostridia.D_3__Clostridiales.D_4__Lachnospiraceae.D_5__Roseburia	D_0__Bacteria.D_1__Firmicutes.D_2__Clostridia.D_3__Clostridiales.D_4__Lachnospiraceae.D_5__Coprococcus.1	D_0__Bacteria.D_1__Firmicutes.D_2__Clostridia.D_3__Clostridiales.D_4__Family.XI.D_5__Anaerococcus		
	D_0__Bacteria.D_1__Firmicutes.D_2__Clostridia.D_3__Clostridiales.D_4__Lachnospiraceae.D_5__Shuttleworthia	D_0__Bacteria.D_1__Firmicutes.D_2__Clostridia.D_3__Clostridiales.D_4__Lachnospiraceae.D_5__Coprococcus.2	D_0__Bacteria.D_1__Firmicutes.D_2__Clostridia.D_3__Clostridiales.D_4__Family.XI.D_5__Ezakiella		
	D_0__Bacteria.D_1__Firmicutes.D_2__Clostridia.D_3__Clostridiales.D_4__Lachnospiraceae.D_5__Tyzzerella.4	D_0__Bacteria.D_1__Firmicutes.D_2__Clostridia.D_3__Clostridiales.D_4__Lachnospiraceae.D_5__Coprococcus.3	D_0__Bacteria.D_1__Firmicutes.D_2__Clostridia.D_3__Clostridiales.D_4__Family.XI.D_5__Finegoldia		
	D_0__Bacteria.D_1__Firmicutes.D_2__Clostridia.D_3__Clostridiales.D_4__Lachnospiraceae.D_5__.Eubacterium..eligens.group	D_0__Bacteria.D_1__Firmicutes.D_2__Clostridia.D_3__Clostridiales.D_4__Lachnospiraceae.D_5__Dorea	D_0__Bacteria.D_1__Firmicutes.D_2__Clostridia.D_3__Clostridiales.D_4__Family.XI.D_5__Parvimonas		
	D_0__Bacteria.D_1__Firmicutes.D_2__Clostridia.D_3__Clostridiales.D_4__Lachnospiraceae.D_5__.Eubacterium..hallii.group	D_0__Bacteria.D_1__Firmicutes.D_2__Clostridia.D_3__Clostridiales.D_4__Lachnospiraceae.D_5__Epulopiscium	D_0__Bacteria.D_1__Firmicutes.D_2__Clostridia.D_3__Clostridiales.D_4__Family.XI.D_5__Peptoniphilus		
	D_0__Bacteria.D_1__Firmicutes.D_2__Clostridia.D_3__Clostridiales.D_4__Lachnospiraceae.D_5__.Eubacterium..oxidoreducens.group	D_0__Bacteria.D_1__Firmicutes.D_2__Clostridia.D_3__Clostridiales.D_4__Lachnospiraceae.D_5__Fusicatenibacter	D_0__Bacteria.D_1__Firmicutes.D_2__Clostridia.D_3__Clostridiales.D_4__Family.XI.D_5__W5053		
	D_0__Bacteria.D_1__Firmicutes.D_2__Clostridia.D_3__Clostridiales.D_4__Lachnospiraceae.D_5__.Eubacterium..ruminantium.group	D_0__Bacteria.D_1__Firmicutes.D_2__Clostridia.D_3__Clostridiales.D_4__Lachnospiraceae.D_5__Howardella	D_0__Bacteria.D_1__Firmicutes.D_2__Clostridia.D_3__Clostridiales.D_4__Family.XIII.D_5__S5.A14a		
	D_0__Bacteria.D_1__Firmicutes.D_2__Clostridia.D_3__Clostridiales.D_4__Lachnospiraceae.D_5__.Eubacterium..ventriosum.group	D_0__Bacteria.D_1__Firmicutes.D_2__Clostridia.D_3__Clostridiales.D_4__Lachnospiraceae.D_5__Lachnoclostridium	D_0__Bacteria.D_1__Firmicutes.D_2__Clostridia.D_3__Clostridiales.D_4__Lachnospiraceae.D_5__Agathobacter		
	D_0__Bacteria.D_1__Firmicutes.D_2__Clostridia.D_3__Clostridiales.D_4__Lachnospiraceae.D_5__.Ruminococcus..gauvreauii.group	D_0__Bacteria.D_1__Firmicutes.D_2__Clostridia.D_3__Clostridiales.D_4__Lachnospiraceae.D_5__Lachnospira	D_0__Bacteria.D_1__Firmicutes.D_2__Clostridia.D_3__Clostridiales.D_4__Lachnospiraceae.D_5__Anaerocolumna		
	D_0__Bacteria.D_1__Firmicutes.D_2__Clostridia.D_3__Clostridiales.D_4__Lachnospiraceae.D_5__.Ruminococcus..gnavus.group	D_0__Bacteria.D_1__Firmicutes.D_2__Clostridia.D_3__Clostridiales.D_4__Lachnospiraceae.D_5__Lachnospiraceae.NC2004.group	D_0__Bacteria.D_1__Firmicutes.D_2__Clostridia.D_3__Clostridiales.D_4__Lachnospiraceae.D_5__Blautia		
	D_0__Bacteria.D_1__Firmicutes.D_2__Clostridia.D_3__Clostridiales.D_4__Lachnospiraceae.D_5__.Ruminococcus..torques.group	D_0__Bacteria.D_1__Firmicutes.D_2__Clostridia.D_3__Clostridiales.D_4__Lachnospiraceae.D_5__Lachnospiraceae.ND3007.group	D_0__Bacteria.D_1__Firmicutes.D_2__Clostridia.D_3__Clostridiales.D_4__Lachnospiraceae.D_5__Coprococcus.3		
	D_0__Bacteria.D_1__Firmicutes.D_2__Clostridia.D_3__Clostridiales.D_4__Lachnospiraceae.__	D_0__Bacteria.D_1__Firmicutes.D_2__Clostridia.D_3__Clostridiales.D_4__Lachnospiraceae.D_5__Lachnospiraceae.NK3A20.group	D_0__Bacteria.D_1__Firmicutes.D_2__Clostridia.D_3__Clostridiales.D_4__Lachnospiraceae.D_5__Dorea		
	D_0__Bacteria.D_1__Firmicutes.D_2__Clostridia.D_3__Clostridiales.D_4__Peptococcaceae.D_5__Peptococcus	D_0__Bacteria.D_1__Firmicutes.D_2__Clostridia.D_3__Clostridiales.D_4__Lachnospiraceae.D_5__Lachnospiraceae.NK4A136.group	D_0__Bacteria.D_1__Firmicutes.D_2__Clostridia.D_3__Clostridiales.D_4__Lachnospiraceae.D_5__Howardella		
	D_0__Bacteria.D_1__Firmicutes.D_2__Clostridia.D_3__Clostridiales.D_4__Peptostreptococcaceae.D_5__Filifactor	D_0__Bacteria.D_1__Firmicutes.D_2__Clostridia.D_3__Clostridiales.D_4__Lachnospiraceae.D_5__Marvinbryantia	D_0__Bacteria.D_1__Firmicutes.D_2__Clostridia.D_3__Clostridiales.D_4__Lachnospiraceae.D_5__Lachnoclostridium		
	D_0__Bacteria.D_1__Firmicutes.D_2__Clostridia.D_3__Clostridiales.D_4__Peptostreptococcaceae.D_5__Intestinibacter	D_0__Bacteria.D_1__Firmicutes.D_2__Clostridia.D_3__Clostridiales.D_4__Lachnospiraceae.D_5__Moryella	D_0__Bacteria.D_1__Firmicutes.D_2__Clostridia.D_3__Clostridiales.D_4__Lachnospiraceae.D_5__Lachnospira		
	D_0__Bacteria.D_1__Firmicutes.D_2__Clostridia.D_3__Clostridiales.D_4__Peptostreptococcaceae.D_5__Peptostreptococcus	D_0__Bacteria.D_1__Firmicutes.D_2__Clostridia.D_3__Clostridiales.D_4__Lachnospiraceae.D_5__Oribacterium	D_0__Bacteria.D_1__Firmicutes.D_2__Clostridia.D_3__Clostridiales.D_4__Lachnospiraceae.D_5__Lachnospiraceae.NC2004.group		
	D_0__Bacteria.D_1__Firmicutes.D_2__Clostridia.D_3__Clostridiales.D_4__Peptostreptococcaceae.D_5__Romboutsia	D_0__Bacteria.D_1__Firmicutes.D_2__Clostridia.D_3__Clostridiales.D_4__Lachnospiraceae.D_5__Roseburia	D_0__Bacteria.D_1__Firmicutes.D_2__Clostridia.D_3__Clostridiales.D_4__Lachnospiraceae.D_5__Lachnospiraceae.NK4A136.group		
	D_0__Bacteria.D_1__Firmicutes.D_2__Clostridia.D_3__Clostridiales.D_4__Peptostreptococcaceae.D_5__Sporacetigenium	D_0__Bacteria.D_1__Firmicutes.D_2__Clostridia.D_3__Clostridiales.D_4__Lachnospiraceae.D_5__Shuttleworthia	D_0__Bacteria.D_1__Firmicutes.D_2__Clostridia.D_3__Clostridiales.D_4__Lachnospiraceae.D_5__Lachnospiraceae.UCG.001		
	D_0__Bacteria.D_1__Firmicutes.D_2__Clostridia.D_3__Clostridiales.D_4__Peptostreptococcaceae.D_5__Terrisporobacter	D_0__Bacteria.D_1__Firmicutes.D_2__Clostridia.D_3__Clostridiales.D_4__Lachnospiraceae.D_5__.Eubacterium..eligens.group	D_0__Bacteria.D_1__Firmicutes.D_2__Clostridia.D_3__Clostridiales.D_4__Lachnospiraceae.D_5__Lachnospiraceae.UCG.004		
	D_0__Bacteria.D_1__Firmicutes.D_2__Clostridia.D_3__Clostridiales.D_4__Peptostreptococcaceae.__	D_0__Bacteria.D_1__Firmicutes.D_2__Clostridia.D_3__Clostridiales.D_4__Lachnospiraceae.D_5__.Eubacterium..hallii.group	D_0__Bacteria.D_1__Firmicutes.D_2__Clostridia.D_3__Clostridiales.D_4__Lachnospiraceae.D_5__Lachnospiraceae.UCG.006		
	D_0__Bacteria.D_1__Firmicutes.D_2__Clostridia.D_3__Clostridiales.D_4__Ruminococcaceae.Ambiguous_taxa	D_0__Bacteria.D_1__Firmicutes.D_2__Clostridia.D_3__Clostridiales.D_4__Lachnospiraceae.D_5__.Eubacterium..oxidoreducens.group	D_0__Bacteria.D_1__Firmicutes.D_2__Clostridia.D_3__Clostridiales.D_4__Lachnospiraceae.D_5__Marvinbryantia		
	D_0__Bacteria.D_1__Firmicutes.D_2__Clostridia.D_3__Clostridiales.D_4__Ruminococcaceae.D_5__Butyricicoccus	D_0__Bacteria.D_1__Firmicutes.D_2__Clostridia.D_3__Clostridiales.D_4__Lachnospiraceae.D_5__.Eubacterium..ruminantium.group	D_0__Bacteria.D_1__Firmicutes.D_2__Clostridia.D_3__Clostridiales.D_4__Lachnospiraceae.D_5__Moryella		
	D_0__Bacteria.D_1__Firmicutes.D_2__Clostridia.D_3__Clostridiales.D_4__Ruminococcaceae.D_5__Candidatus.Soleaferrea	D_0__Bacteria.D_1__Firmicutes.D_2__Clostridia.D_3__Clostridiales.D_4__Lachnospiraceae.D_5__.Eubacterium..xylanophilum.group	D_0__Bacteria.D_1__Firmicutes.D_2__Clostridia.D_3__Clostridiales.D_4__Lachnospiraceae.D_5__Roseburia		
	D_0__Bacteria.D_1__Firmicutes.D_2__Clostridia.D_3__Clostridiales.D_4__Ruminococcaceae.D_5__Faecalibacterium	D_0__Bacteria.D_1__Firmicutes.D_2__Clostridia.D_3__Clostridiales.D_4__Lachnospiraceae.D_5__.Ruminococcus..gauvreauii.group	D_0__Bacteria.D_1__Firmicutes.D_2__Clostridia.D_3__Clostridiales.D_4__Lachnospiraceae.D_5__Shuttleworthia		
	D_0__Bacteria.D_1__Firmicutes.D_2__Clostridia.D_3__Clostridiales.D_4__Ruminococcaceae.D_5__Fastidiosipila	D_0__Bacteria.D_1__Firmicutes.D_2__Clostridia.D_3__Clostridiales.D_4__Lachnospiraceae.D_5__.Ruminococcus..gnavus.group	D_0__Bacteria.D_1__Firmicutes.D_2__Clostridia.D_3__Clostridiales.D_4__Lachnospiraceae.D_5__.Eubacterium..eligens.group		
	D_0__Bacteria.D_1__Firmicutes.D_2__Clostridia.D_3__Clostridiales.D_4__Ruminococcaceae.D_5__Intestinimonas	D_0__Bacteria.D_1__Firmicutes.D_2__Clostridia.D_3__Clostridiales.D_4__Lachnospiraceae.D_5__.Ruminococcus..torques.group	D_0__Bacteria.D_1__Firmicutes.D_2__Clostridia.D_3__Clostridiales.D_4__Lachnospiraceae.D_5__.Eubacterium..ruminantium.group		
	D_0__Bacteria.D_1__Firmicutes.D_2__Clostridia.D_3__Clostridiales.D_4__Ruminococcaceae.D_5__Ruminiclostridium.5	D_0__Bacteria.D_1__Firmicutes.D_2__Clostridia.D_3__Clostridiales.D_4__Lachnospiraceae.__	D_0__Bacteria.D_1__Firmicutes.D_2__Clostridia.D_3__Clostridiales.D_4__Lachnospiraceae.D_5__.Ruminococcus..gnavus.group		
	D_0__Bacteria.D_1__Firmicutes.D_2__Clostridia.D_3__Clostridiales.D_4__Ruminococcaceae.D_5__Ruminiclostridium.6	D_0__Bacteria.D_1__Firmicutes.D_2__Clostridia.D_3__Clostridiales.D_4__Peptococcaceae.D_5__Peptococcus	D_0__Bacteria.D_1__Firmicutes.D_2__Clostridia.D_3__Clostridiales.D_4__Lachnospiraceae.D_5__.Ruminococcus..torques.group		
	D_0__Bacteria.D_1__Firmicutes.D_2__Clostridia.D_3__Clostridiales.D_4__Ruminococcaceae.D_5__Ruminococcaceae.NK4A214.group	D_0__Bacteria.D_1__Firmicutes.D_2__Clostridia.D_3__Clostridiales.D_4__Peptostreptococcaceae.D_5__Filifactor	D_0__Bacteria.D_1__Firmicutes.D_2__Clostridia.D_3__Clostridiales.D_4__Lachnospiraceae.D_5__uncultured		
	D_0__Bacteria.D_1__Firmicutes.D_2__Clostridia.D_3__Clostridiales.D_4__Ruminococcaceae.D_5__Ruminococcaceae.UCG.002	D_0__Bacteria.D_1__Firmicutes.D_2__Clostridia.D_3__Clostridiales.D_4__Peptostreptococcaceae.D_5__Intestinibacter	D_0__Bacteria.D_1__Firmicutes.D_2__Clostridia.D_3__Clostridiales.D_4__Lachnospiraceae.__		
	D_0__Bacteria.D_1__Firmicutes.D_2__Clostridia.D_3__Clostridiales.D_4__Ruminococcaceae.D_5__Ruminococcaceae.UCG.003	D_0__Bacteria.D_1__Firmicutes.D_2__Clostridia.D_3__Clostridiales.D_4__Peptostreptococcaceae.D_5__Peptostreptococcus	D_0__Bacteria.D_1__Firmicutes.D_2__Clostridia.D_3__Clostridiales.D_4__Peptococcaceae.D_5__Peptococcus		
	D_0__Bacteria.D_1__Firmicutes.D_2__Clostridia.D_3__Clostridiales.D_4__Ruminococcaceae.D_5__Ruminococcaceae.UCG.004	D_0__Bacteria.D_1__Firmicutes.D_2__Clostridia.D_3__Clostridiales.D_4__Peptostreptococcaceae.D_5__Romboutsia	D_0__Bacteria.D_1__Firmicutes.D_2__Clostridia.D_3__Clostridiales.D_4__Peptococcaceae.D_5__uncultured		
	D_0__Bacteria.D_1__Firmicutes.D_2__Clostridia.D_3__Clostridiales.D_4__Ruminococcaceae.D_5__Ruminococcaceae.UCG.005	D_0__Bacteria.D_1__Firmicutes.D_2__Clostridia.D_3__Clostridiales.D_4__Peptostreptococcaceae.D_5__Terrisporobacter	D_0__Bacteria.D_1__Firmicutes.D_2__Clostridia.D_3__Clostridiales.D_4__Peptostreptococcaceae.D_5__Clostridioides		
	D_0__Bacteria.D_1__Firmicutes.D_2__Clostridia.D_3__Clostridiales.D_4__Ruminococcaceae.D_5__Ruminococcaceae.UCG.010	D_0__Bacteria.D_1__Firmicutes.D_2__Clostridia.D_3__Clostridiales.D_4__Peptostreptococcaceae.__	D_0__Bacteria.D_1__Firmicutes.D_2__Clostridia.D_3__Clostridiales.D_4__Peptostreptococcaceae.D_5__Peptostreptococcus		
	D_0__Bacteria.D_1__Firmicutes.D_2__Clostridia.D_3__Clostridiales.D_4__Ruminococcaceae.D_5__Ruminococcaceae.UCG.011	D_0__Bacteria.D_1__Firmicutes.D_2__Clostridia.D_3__Clostridiales.D_4__Ruminococcaceae.D_5__Butyricicoccus	D_0__Bacteria.D_1__Firmicutes.D_2__Clostridia.D_3__Clostridiales.D_4__Peptostreptococcaceae.D_5__Romboutsia		
	D_0__Bacteria.D_1__Firmicutes.D_2__Clostridia.D_3__Clostridiales.D_4__Ruminococcaceae.D_5__Ruminococcaceae.UCG.013	D_0__Bacteria.D_1__Firmicutes.D_2__Clostridia.D_3__Clostridiales.D_4__Ruminococcaceae.D_5__Faecalibacterium	D_0__Bacteria.D_1__Firmicutes.D_2__Clostridia.D_3__Clostridiales.D_4__Peptostreptococcaceae.D_5__Sporacetigenium		

OTUs present among HC	OTUs present among Cases_Day1	OTUs present among Cases_Day15	OTUs present among Cases_Day30	OTUs present among only Cases_Day30_A	OTUs present among only Cases_Day30_B
	D_0__Bacteria.D_1__Firmicutes.D_2__Clostridia.D_3__Clostridiales.D_4__Ruminococcaceae.D_5__Ruminococcaceae.UCG.014	D_0__Bacteria.D_1__Firmicutes.D_2__Clostridia.D_3__Clostridiales.D_4__Ruminococcaceae.D_5__Fastidiosipila	D_0__Bacteria.D_1__Firmicutes.D_2__Clostridia.D_3__Clostridiales.D_4__Ruminococcaceae.D_5__Butyricicoccus		
	D_0__Bacteria.D_1__Firmicutes.D_2__Clostridia.D_3__Clostridiales.D_4__Ruminococcaceae.D_5__Ruminococcus.1	D_0__Bacteria.D_1__Firmicutes.D_2__Clostridia.D_3__Clostridiales.D_4__Ruminococcaceae.D_5__Flavonifractor	D_0__Bacteria.D_1__Firmicutes.D_2__Clostridia.D_3__Clostridiales.D_4__Ruminococcaceae.D_5__Faecalibacterium		
	D_0__Bacteria.D_1__Firmicutes.D_2__Clostridia.D_3__Clostridiales.D_4__Ruminococcaceae.D_5__Ruminococcus.2	D_0__Bacteria.D_1__Firmicutes.D_2__Clostridia.D_3__Clostridiales.D_4__Ruminococcaceae.D_5__Fournierella	D_0__Bacteria.D_1__Firmicutes.D_2__Clostridia.D_3__Clostridiales.D_4__Ruminococcaceae.D_5__Fastidiosipila		
	D_0__Bacteria.D_1__Firmicutes.D_2__Clostridia.D_3__Clostridiales.D_4__Ruminococcaceae.D_5__Saccharofermentans	D_0__Bacteria.D_1__Firmicutes.D_2__Clostridia.D_3__Clostridiales.D_4__Ruminococcaceae.D_5__GCA.900066225	D_0__Bacteria.D_1__Firmicutes.D_2__Clostridia.D_3__Clostridiales.D_4__Ruminococcaceae.D_5__Flavonifractor		
	D_0__Bacteria.D_1__Firmicutes.D_2__Clostridia.D_3__Clostridiales.D_4__Ruminococcaceae.D_5__Subdoligranulum	D_0__Bacteria.D_1__Firmicutes.D_2__Clostridia.D_3__Clostridiales.D_4__Ruminococcaceae.D_5__Intestinimonas	D_0__Bacteria.D_1__Firmicutes.D_2__Clostridia.D_3__Clostridiales.D_4__Ruminococcaceae.D_5__Oscillibacter		
	D_0__Bacteria.D_1__Firmicutes.D_2__Clostridia.D_3__Clostridiales.D_4__Ruminococcaceae.D_5__UBA1819	D_0__Bacteria.D_1__Firmicutes.D_2__Clostridia.D_3__Clostridiales.D_4__Ruminococcaceae.D_5__Ruminiclostridium.1	D_0__Bacteria.D_1__Firmicutes.D_2__Clostridia.D_3__Clostridiales.D_4__Ruminococcaceae.D_5__Ruminiclostridium.6		
	D_0__Bacteria.D_1__Firmicutes.D_2__Clostridia.D_3__Clostridiales.D_4__Ruminococcaceae.D_5__.Eubacterium..coprostanoligenes.group	D_0__Bacteria.D_1__Firmicutes.D_2__Clostridia.D_3__Clostridiales.D_4__Ruminococcaceae.D_5__Ruminiclostridium.5	D_0__Bacteria.D_1__Firmicutes.D_2__Clostridia.D_3__Clostridiales.D_4__Ruminococcaceae.D_5__Ruminiclostridium.9		
	D_0__Bacteria.D_1__Firmicutes.D_2__Clostridia.D_3__Clostridiales.D_4__Ruminococcaceae.D_5__uncultured	D_0__Bacteria.D_1__Firmicutes.D_2__Clostridia.D_3__Clostridiales.D_4__Ruminococcaceae.D_5__Ruminiclostridium.6	D_0__Bacteria.D_1__Firmicutes.D_2__Clostridia.D_3__Clostridiales.D_4__Ruminococcaceae.D_5__Ruminococcaceae.NK4A214.group		
	D_0__Bacteria.D_1__Firmicutes.D_2__Clostridia.D_3__Clostridiales.D_4__Ruminococcaceae.__	D_0__Bacteria.D_1__Firmicutes.D_2__Clostridia.D_3__Clostridiales.D_4__Ruminococcaceae.D_5__Ruminococcaceae.NK4A214.group	D_0__Bacteria.D_1__Firmicutes.D_2__Clostridia.D_3__Clostridiales.D_4__Ruminococcaceae.D_5__Ruminococcaceae.UCG.002		
	D_0__Bacteria.D_1__Firmicutes.D_2__Clostridia.D_3__Clostridiales.__.__	D_0__Bacteria.D_1__Firmicutes.D_2__Clostridia.D_3__Clostridiales.D_4__Ruminococcaceae.D_5__Ruminococcaceae.UCG.002	D_0__Bacteria.D_1__Firmicutes.D_2__Clostridia.D_3__Clostridiales.D_4__Ruminococcaceae.D_5__Ruminococcaceae.UCG.003		
	D_0__Bacteria.D_1__Firmicutes.D_2__Erysipelotrichia.D_3__Erysipelotrichales.D_4__Erysipelotrichaceae.D_5__Asteroleplasma	D_0__Bacteria.D_1__Firmicutes.D_2__Clostridia.D_3__Clostridiales.D_4__Ruminococcaceae.D_5__Ruminococcaceae.UCG.003	D_0__Bacteria.D_1__Firmicutes.D_2__Clostridia.D_3__Clostridiales.D_4__Ruminococcaceae.D_5__Ruminococcaceae.UCG.005		
	D_0__Bacteria.D_1__Firmicutes.D_2__Erysipelotrichia.D_3__Erysipelotrichales.D_4__Erysipelotrichaceae.D_5__Catenibacterium	D_0__Bacteria.D_1__Firmicutes.D_2__Clostridia.D_3__Clostridiales.D_4__Ruminococcaceae.D_5__Ruminococcaceae.UCG.004	D_0__Bacteria.D_1__Firmicutes.D_2__Clostridia.D_3__Clostridiales.D_4__Ruminococcaceae.D_5__Ruminococcaceae.UCG.008		
	D_0__Bacteria.D_1__Firmicutes.D_2__Erysipelotrichia.D_3__Erysipelotrichales.D_4__Erysipelotrichaceae.D_5__Erysipelatoclostridium	D_0__Bacteria.D_1__Firmicutes.D_2__Clostridia.D_3__Clostridiales.D_4__Ruminococcaceae.D_5__Ruminococcaceae.UCG.005	D_0__Bacteria.D_1__Firmicutes.D_2__Clostridia.D_3__Clostridiales.D_4__Ruminococcaceae.D_5__Ruminococcaceae.UCG.010		
	D_0__Bacteria.D_1__Firmicutes.D_2__Erysipelotrichia.D_3__Erysipelotrichales.D_4__Erysipelotrichaceae.D_5__Erysipelothrix	D_0__Bacteria.D_1__Firmicutes.D_2__Clostridia.D_3__Clostridiales.D_4__Ruminococcaceae.D_5__Ruminococcaceae.UCG.008	D_0__Bacteria.D_1__Firmicutes.D_2__Clostridia.D_3__Clostridiales.D_4__Ruminococcaceae.D_5__Ruminococcaceae.UCG.013		
	D_0__Bacteria.D_1__Firmicutes.D_2__Erysipelotrichia.D_3__Erysipelotrichales.D_4__Erysipelotrichaceae.D_5__Erysipelotrichaceae.UCG.003	D_0__Bacteria.D_1__Firmicutes.D_2__Clostridia.D_3__Clostridiales.D_4__Ruminococcaceae.D_5__Ruminococcaceae.UCG.010	D_0__Bacteria.D_1__Firmicutes.D_2__Clostridia.D_3__Clostridiales.D_4__Ruminococcaceae.D_5__Ruminococcaceae.UCG.014		
	D_0__Bacteria.D_1__Firmicutes.D_2__Erysipelotrichia.D_3__Erysipelotrichales.D_4__Erysipelotrichaceae.D_5__Holdemanella	D_0__Bacteria.D_1__Firmicutes.D_2__Clostridia.D_3__Clostridiales.D_4__Ruminococcaceae.D_5__Ruminococcaceae.UCG.013	D_0__Bacteria.D_1__Firmicutes.D_2__Clostridia.D_3__Clostridiales.D_4__Ruminococcaceae.D_5__Ruminococcus.1		
	D_0__Bacteria.D_1__Firmicutes.D_2__Erysipelotrichia.D_3__Erysipelotrichales.D_4__Erysipelotrichaceae.D_5__Solobacterium	D_0__Bacteria.D_1__Firmicutes.D_2__Clostridia.D_3__Clostridiales.D_4__Ruminococcaceae.D_5__Ruminococcaceae.UCG.014	D_0__Bacteria.D_1__Firmicutes.D_2__Clostridia.D_3__Clostridiales.D_4__Ruminococcaceae.D_5__Ruminococcus.2		
	D_0__Bacteria.D_1__Firmicutes.D_2__Erysipelotrichia.D_3__Erysipelotrichales.D_4__Erysipelotrichaceae.D_5__Turicibacter	D_0__Bacteria.D_1__Firmicutes.D_2__Clostridia.D_3__Clostridiales.D_4__Ruminococcaceae.D_5__Ruminococcus.1	D_0__Bacteria.D_1__Firmicutes.D_2__Clostridia.D_3__Clostridiales.D_4__Ruminococcaceae.D_5__Subdoligranulum		
	D_0__Bacteria.D_1__Firmicutes.D_2__Erysipelotrichia.D_3__Erysipelotrichales.D_4__Erysipelotrichaceae.D_5__.Clostridium..innocuum.group	D_0__Bacteria.D_1__Firmicutes.D_2__Clostridia.D_3__Clostridiales.D_4__Ruminococcaceae.D_5__Ruminococcus.2	D_0__Bacteria.D_1__Firmicutes.D_2__Clostridia.D_3__Clostridiales.D_4__Ruminococcaceae.D_5__UBA1819		
	D_0__Bacteria.D_1__Firmicutes.D_2__Negativicutes.D_3__Selenomonadales.D_4__Acidaminococcaceae.D_5__Acidaminococcus	D_0__Bacteria.D_1__Firmicutes.D_2__Clostridia.D_3__Clostridiales.D_4__Ruminococcaceae.D_5__Saccharofermentans	D_0__Bacteria.D_1__Firmicutes.D_2__Clostridia.D_3__Clostridiales.D_4__Ruminococcaceae.D_5__.Eubacterium..coprostanoligenes.group		
	D_0__Bacteria.D_1__Firmicutes.D_2__Negativicutes.D_3__Selenomonadales.D_4__Acidaminococcaceae.D_5__Phascolarctobacterium	D_0__Bacteria.D_1__Firmicutes.D_2__Clostridia.D_3__Clostridiales.D_4__Ruminococcaceae.D_5__Subdoligranulum	D_0__Bacteria.D_1__Firmicutes.D_2__Clostridia.D_3__Clostridiales.D_4__Ruminococcaceae.D_5__uncultured		
	D_0__Bacteria.D_1__Firmicutes.D_2__Negativicutes.D_3__Selenomonadales.D_4__Veillonellaceae.D_5__Dialister	D_0__Bacteria.D_1__Firmicutes.D_2__Clostridia.D_3__Clostridiales.D_4__Ruminococcaceae.D_5__.Eubacterium..coprostanoligenes.group	D_0__Bacteria.D_1__Firmicutes.D_2__Clostridia.D_3__Clostridiales.D_4__Ruminococcaceae.__		
	D_0__Bacteria.D_1__Firmicutes.D_2__Negativicutes.D_3__Selenomonadales.D_4__Veillonellaceae.D_5__Megamonas	D_0__Bacteria.D_1__Firmicutes.D_2__Clostridia.D_3__Clostridiales.D_4__Ruminococcaceae.D_5__uncultured	D_0__Bacteria.D_1__Firmicutes.D_2__Erysipelotrichia.D_3__Erysipelotrichales.D_4__Erysipelotrichaceae.D_5__Allobaculum		
	D_0__Bacteria.D_1__Firmicutes.D_2__Negativicutes.D_3__Selenomonadales.D_4__Veillonellaceae.D_5__Megasphaera	D_0__Bacteria.D_1__Firmicutes.D_2__Clostridia.D_3__Clostridiales.D_4__Ruminococcaceae.__	D_0__Bacteria.D_1__Firmicutes.D_2__Erysipelotrichia.D_3__Erysipelotrichales.D_4__Erysipelotrichaceae.D_5__Asteroleplasma		
	D_0__Bacteria.D_1__Firmicutes.D_2__Negativicutes.D_3__Selenomonadales.D_4__Veillonellaceae.D_5__Mitsuokella	D_0__Bacteria.D_1__Firmicutes.D_2__Clostridia.D_3__Clostridiales.__.__	D_0__Bacteria.D_1__Firmicutes.D_2__Erysipelotrichia.D_3__Erysipelotrichales.D_4__Erysipelotrichaceae.D_5__Candidatus.Stoquefichus		
	D_0__Bacteria.D_1__Firmicutes.D_2__Negativicutes.D_3__Selenomonadales.D_4__Veillonellaceae.D_5__Veillonella	D_0__Bacteria.D_1__Firmicutes.D_2__Erysipelotrichia.D_3__Erysipelotrichales.D_4__Erysipelotrichaceae.D_5__Asteroleplasma	D_0__Bacteria.D_1__Firmicutes.D_2__Erysipelotrichia.D_3__Erysipelotrichales.D_4__Erysipelotrichaceae.D_5__Catenibacterium		
	D_0__Bacteria.D_1__Firmicutes.D_2__Negativicutes.D_3__Selenomonadales.D_4__Veillonellaceae.__	D_0__Bacteria.D_1__Firmicutes.D_2__Erysipelotrichia.D_3__Erysipelotrichales.D_4__Erysipelotrichaceae.D_5__Catenibacterium	D_0__Bacteria.D_1__Firmicutes.D_2__Erysipelotrichia.D_3__Erysipelotrichales.D_4__Erysipelotrichaceae.D_5__Dubosiella		
	D_0__Bacteria.D_1__Fusobacteria.D_2__Fusobacteriia.D_3__Fusobacteriales.D_4__Fusobacteriaceae.D_5__Cetobacterium	D_0__Bacteria.D_1__Firmicutes.D_2__Erysipelotrichia.D_3__Erysipelotrichales.D_4__Erysipelotrichaceae.D_5__Erysipelothrix	D_0__Bacteria.D_1__Firmicutes.D_2__Erysipelotrichia.D_3__Erysipelotrichales.D_4__Erysipelotrichaceae.D_5__Erysipelatoclostridium		
	D_0__Bacteria.D_1__Fusobacteria.D_2__Fusobacteriia.D_3__Fusobacteriales.D_4__Fusobacteriaceae.D_5__Fusobacterium	D_0__Bacteria.D_1__Firmicutes.D_2__Erysipelotrichia.D_3__Erysipelotrichales.D_4__Erysipelotrichaceae.D_5__Erysipelotrichaceae.UCG.003	D_0__Bacteria.D_1__Firmicutes.D_2__Erysipelotrichia.D_3__Erysipelotrichales.D_4__Erysipelotrichaceae.D_5__Erysipelothrix		
	D_0__Bacteria.D_1__Fusobacteria.D_2__Fusobacteriia.D_3__Fusobacteriales.D_4__Leptotrichiaceae.D_5__Sneathia	D_0__Bacteria.D_1__Firmicutes.D_2__Erysipelotrichia.D_3__Erysipelotrichales.D_4__Erysipelotrichaceae.D_5__Holdemanella	D_0__Bacteria.D_1__Firmicutes.D_2__Erysipelotrichia.D_3__Erysipelotrichales.D_4__Erysipelotrichaceae.D_5__Faecalibaculum		
	D_0__Bacteria.D_1__Lentisphaerae.D_2__Lentisphaeria.D_3__Victivallales.D_4__vadinBE97.D_5__uncultured.bacterium	D_0__Bacteria.D_1__Firmicutes.D_2__Erysipelotrichia.D_3__Erysipelotrichales.D_4__Erysipelotrichaceae.D_5__Solobacterium	D_0__Bacteria.D_1__Firmicutes.D_2__Erysipelotrichia.D_3__Erysipelotrichales.D_4__Erysipelotrichaceae.D_5__Holdemanella		
	D_0__Bacteria.D_1__Patescibacteria.D_2__Saccharimonadia.D_3__Saccharimonadales.D_4__Saccharimonadaceae.D_5__Candidatus.Saccharimonas	D_0__Bacteria.D_1__Firmicutes.D_2__Erysipelotrichia.D_3__Erysipelotrichales.D_4__Erysipelotrichaceae.D_5__Turicibacter	D_0__Bacteria.D_1__Firmicutes.D_2__Erysipelotrichia.D_3__Erysipelotrichales.D_4__Erysipelotrichaceae.D_5__.Clostridium..innocuum.group		
	D_0__Bacteria.D_1__Patescibacteria.D_2__Saccharimonadia.D_3__Saccharimonadales.D_4__Saccharimonadaceae.__	D_0__Bacteria.D_1__Firmicutes.D_2__Erysipelotrichia.D_3__Erysipelotrichales.D_4__Erysipelotrichaceae.__	D_0__Bacteria.D_1__Firmicutes.D_2__Erysipelotrichia.D_3__Erysipelotrichales.D_4__Erysipelotrichaceae.D_5__uncultured		
	D_0__Bacteria.D_1__Patescibacteria.D_2__Saccharimonadia.D_3__Saccharimonadales.__.__	D_0__Bacteria.D_1__Firmicutes.D_2__Negativicutes.D_3__Selenomonadales.D_4__Acidaminococcaceae.D_5__Acidaminococcus	D_0__Bacteria.D_1__Firmicutes.D_2__Negativicutes.D_3__Selenomonadales.D_4__Acidaminococcaceae.D_5__Acidaminococcus		
	D_0__Bacteria.D_1__Proteobacteria.D_2__Alphaproteobacteria.D_3__Acetobacterales.D_4__Acetobacteraceae.D_5__Roseococcus	D_0__Bacteria.D_1__Firmicutes.D_2__Negativicutes.D_3__Selenomonadales.D_4__Acidaminococcaceae.D_5__Phascolarctobacterium	D_0__Bacteria.D_1__Firmicutes.D_2__Negativicutes.D_3__Selenomonadales.D_4__Acidaminococcaceae.D_5__Phascolarctobacterium		
	D_0__Bacteria.D_1__Proteobacteria.D_2__Alphaproteobacteria.D_3__Caulobacterales.D_4__Caulobacteraceae.D_5__Asticcacaulis	D_0__Bacteria.D_1__Firmicutes.D_2__Negativicutes.D_3__Selenomonadales.D_4__Veillonellaceae.D_5__Dialister	D_0__Bacteria.D_1__Firmicutes.D_2__Negativicutes.D_3__Selenomonadales.D_4__Veillonellaceae.D_5__Allisonella		
	D_0__Bacteria.D_1__Proteobacteria.D_2__Alphaproteobacteria.D_3__Caulobacterales.D_4__Caulobacteraceae.D_5__Brevundimonas	D_0__Bacteria.D_1__Firmicutes.D_2__Negativicutes.D_3__Selenomonadales.D_4__Veillonellaceae.D_5__Megamonas	D_0__Bacteria.D_1__Firmicutes.D_2__Negativicutes.D_3__Selenomonadales.D_4__Veillonellaceae.D_5__Anaerovibrio		
	D_0__Bacteria.D_1__Proteobacteria.D_2__Alphaproteobacteria.D_3__Caulobacterales.D_4__Caulobacteraceae.D_5__Caulobacter	D_0__Bacteria.D_1__Firmicutes.D_2__Negativicutes.D_3__Selenomonadales.D_4__Veillonellaceae.D_5__Megasphaera	D_0__Bacteria.D_1__Firmicutes.D_2__Negativicutes.D_3__Selenomonadales.D_4__Veillonellaceae.D_5__Dialister		
	D_0__Bacteria.D_1__Proteobacteria.D_2__Alphaproteobacteria.D_3__Caulobacterales.D_4__Caulobacteraceae.D_5__Phenylobacterium	D_0__Bacteria.D_1__Firmicutes.D_2__Negativicutes.D_3__Selenomonadales.D_4__Veillonellaceae.D_5__Mitsuokella	D_0__Bacteria.D_1__Firmicutes.D_2__Negativicutes.D_3__Selenomonadales.D_4__Veillonellaceae.D_5__Megamonas		
	D_0__Bacteria.D_1__Proteobacteria.D_2__Alphaproteobacteria.D_3__Caulobacterales.D_4__Caulobacteraceae.__	D_0__Bacteria.D_1__Firmicutes.D_2__Negativicutes.D_3__Selenomonadales.D_4__Veillonellaceae.D_5__Selenomonas	D_0__Bacteria.D_1__Firmicutes.D_2__Negativicutes.D_3__Selenomonadales.D_4__Veillonellaceae.D_5__Megasphaera		
	D_0__Bacteria.D_1__Proteobacteria.D_2__Alphaproteobacteria.D_3__Reyranellales.D_4__Reyranellaceae.D_5__Reyranella	D_0__Bacteria.D_1__Firmicutes.D_2__Negativicutes.D_3__Selenomonadales.D_4__Veillonellaceae.D_5__Veillonella	D_0__Bacteria.D_1__Firmicutes.D_2__Negativicutes.D_3__Selenomonadales.D_4__Veillonellaceae.D_5__Mitsuokella		
	D_0__Bacteria.D_1__Proteobacteria.D_2__Alphaproteobacteria.D_3__Rhizobiales.D_4__Beijerinckiaceae.D_5__Bosea	D_0__Bacteria.D_1__Firmicutes.D_2__Negativicutes.D_3__Selenomonadales.D_4__Veillonellaceae.__	D_0__Bacteria.D_1__Firmicutes.D_2__Negativicutes.D_3__Selenomonadales.D_4__Veillonellaceae.D_5__Quinella		
	D_0__Bacteria.D_1__Proteobacteria.D_2__Alphaproteobacteria.D_3__Rhizobiales.D_4__Beijerinckiaceae.__	D_0__Bacteria.D_1__Firmicutes.__.__.__.__	D_0__Bacteria.D_1__Firmicutes.D_2__Negativicutes.D_3__Selenomonadales.D_4__Veillonellaceae.D_5__Veillonella		
	D_0__Bacteria.D_1__Proteobacteria.D_2__Alphaproteobacteria.D_3__Rhizobiales.D_4__Devosiaceae.D_5__Devosia	D_0__Bacteria.D_1__Fusobacteria.D_2__Fusobacteriia.D_3__Fusobacteriales.D_4__Leptotrichiaceae.D_5__Leptotrichia	D_0__Bacteria.D_1__Firmicutes.D_2__Negativicutes.D_3__Selenomonadales.D_4__Veillonellaceae.D_5__uncultured		
	D_0__Bacteria.D_1__Proteobacteria.D_2__Alphaproteobacteria.D_3__Rhizobiales.D_4__Devosiaceae.__	D_0__Bacteria.D_1__Fusobacteria.D_2__Fusobacteriia.D_3__Fusobacteriales.D_4__Leptotrichiaceae.D_5__Sneathia	D_0__Bacteria.D_1__Firmicutes.D_2__Negativicutes.D_3__Selenomonadales.D_4__Veillonellaceae.__		
	D_0__Bacteria.D_1__Proteobacteria.D_2__Alphaproteobacteria.D_3__Rhizobiales.D_4__Kaistiaceae.D_5__Kaistia	D_0__Bacteria.D_1__Lentisphaerae.D_2__Lentisphaeria.D_3__Victivallales.D_4__vadinBE97.D_5__uncultured.bacterium	D_0__Bacteria.D_1__Firmicutes.__.__.__.__		
	D_0__Bacteria.D_1__Proteobacteria.D_2__Alphaproteobacteria.D_3__Rhizobiales.D_4__Rhizobiaceae.D_5__Allorhizobium.Neorhizobium.Pararhizobium.Rhizobium	D_0__Bacteria.D_1__Lentisphaerae.D_2__Lentisphaeria.D_3__Victivallales.D_4__vadinBE97.__	D_0__Bacteria.D_1__Fusobacteria.D_2__Fusobacteriia.D_3__Fusobacteriales.D_4__Fusobacteriaceae.D_5__Fusobacterium		
	D_0__Bacteria.D_1__Proteobacteria.D_2__Alphaproteobacteria.D_3__Rhizobiales.D_4__Rhizobiaceae.D_5__Aminobacter	D_0__Bacteria.D_1__Patescibacteria.D_2__Saccharimonadia.D_3__Saccharimonadales.__.__	D_0__Bacteria.D_1__Fusobacteria.D_2__Fusobacteriia.D_3__Fusobacteriales.D_4__Leptotrichiaceae.D_5__Sneathia		
	D_0__Bacteria.D_1__Proteobacteria.D_2__Alphaproteobacteria.D_3__Rhizobiales.D_4__Rhizobiaceae.D_5__Mesorhizobium	D_0__Bacteria.D_1__Proteobacteria.D_2__Alphaproteobacteria.D_3__Acetobacterales.D_4__Acetobacteraceae.D_5__Roseococcus	D_0__Bacteria.D_1__Lentisphaerae.D_2__Lentisphaeria.D_3__Victivallales.D_4__vadinBE97.D_5__uncultured.bacterium		
	D_0__Bacteria.D_1__Proteobacteria.D_2__Alphaproteobacteria.D_3__Rhizobiales.D_4__Rhizobiaceae.D_5__Ochrobactrum	D_0__Bacteria.D_1__Proteobacteria.D_2__Alphaproteobacteria.D_3__Caulobacterales.D_4__Caulobacteraceae.D_5__Asticcacaulis	D_0__Bacteria.D_1__Patescibacteria.D_2__Saccharimonadia.D_3__Saccharimonadales.__.__		
	D_0__Bacteria.D_1__Proteobacteria.D_2__Alphaproteobacteria.D_3__Rhizobiales.D_4__Rhizobiaceae.D_5__Pseudochrobactrum	D_0__Bacteria.D_1__Proteobacteria.D_2__Alphaproteobacteria.D_3__Caulobacterales.D_4__Caulobacteraceae.D_5__Brevundimonas	D_0__Bacteria.D_1__Planctomycetes.D_2__Planctomycetacia.D_3__Isosphaerales.D_4__Isosphaeraceae.D_5__Isosphaera		
	D_0__Bacteria.D_1__Proteobacteria.D_2__Alphaproteobacteria.D_3__Rhizobiales.D_4__Rhizobiaceae.__	D_0__Bacteria.D_1__Proteobacteria.D_2__Alphaproteobacteria.D_3__Caulobacterales.D_4__Caulobacteraceae.D_5__Caulobacter	D_0__Bacteria.D_1__Proteobacteria.D_2__Alphaproteobacteria.D_3__Caulobacterales.D_4__Caulobacteraceae.D_5__Asticcacaulis		
	D_0__Bacteria.D_1__Proteobacteria.D_2__Alphaproteobacteria.D_3__Rhizobiales.D_4__Xanthobacteraceae.D_5__Bradyrhizobium	D_0__Bacteria.D_1__Proteobacteria.D_2__Alphaproteobacteria.D_3__Caulobacterales.D_4__Caulobacteraceae.__	D_0__Bacteria.D_1__Proteobacteria.D_2__Alphaproteobacteria.D_3__Caulobacterales.D_4__Caulobacteraceae.D_5__Brevundimonas		
	D_0__Bacteria.D_1__Proteobacteria.D_2__Alphaproteobacteria.D_3__Rhizobiales.D_4__Xanthobacteraceae.D_5__Rhodopseudomonas	D_0__Bacteria.D_1__Proteobacteria.D_2__Alphaproteobacteria.D_3__Paracaedibacterales.D_4__Paracaedibacteraceae.D_5__Candidatus.Paracaedibacter	D_0__Bacteria.D_1__Proteobacteria.D_2__Alphaproteobacteria.D_3__Caulobacterales.D_4__Caulobacteraceae.D_5__Caulobacter		
	D_0__Bacteria.D_1__Proteobacteria.D_2__Alphaproteobacteria.D_3__Rhodobacterales.D_4__Rhodobacteraceae.D_5__Falsirhodobacter	D_0__Bacteria.D_1__Proteobacteria.D_2__Alphaproteobacteria.D_3__Reyranellales.D_4__Reyranellaceae.D_5__Reyranella	D_0__Bacteria.D_1__Proteobacteria.D_2__Alphaproteobacteria.D_3__Caulobacterales.D_4__Caulobacteraceae.D_5__Phenylobacterium		
	D_0__Bacteria.D_1__Proteobacteria.D_2__Alphaproteobacteria.D_3__Rhodobacterales.D_4__Rhodobacteraceae.D_5__Gemmobacter	D_0__Bacteria.D_1__Proteobacteria.D_2__Alphaproteobacteria.D_3__Rhizobiales.D_4__Beijerinckiaceae.D_5__Bosea	D_0__Bacteria.D_1__Proteobacteria.D_2__Alphaproteobacteria.D_3__Paracaedibacterales.D_4__Paracaedibacteraceae.D_5__Candidatus.Finniella		
	D_0__Bacteria.D_1__Proteobacteria.D_2__Alphaproteobacteria.D_3__Rhodobacterales.D_4__Rhodobacteraceae.D_5__Ketogulonicigenium	D_0__Bacteria.D_1__Proteobacteria.D_2__Alphaproteobacteria.D_3__Rhizobiales.D_4__Beijerinckiaceae.D_5__Methylobacterium	D_0__Bacteria.D_1__Proteobacteria.D_2__Alphaproteobacteria.D_3__Paracaedibacterales.D_4__Paracaedibacteraceae.D_5__uncultured		

OTUs present among HC	OTUs present among Cases_Day1	OTUs present among Cases_Day15	OTUs present among Cases_Day30	OTUs present among only Cases_Day30_A	OTUs present among only Cases_Day30_B
	D_0__Bacteria.D_1__Proteobacteria.D_2__Alphaproteobacteria.D_3__Rhodobacterales.D_4__Rhodobacteraceae.D_5__Paracoccus	D_0__Bacteria.D_1__Proteobacteria.D_2__Alphaproteobacteria.D_3__Rhizobiales.D_4__Beijerinckiaceae.__	D_0__Bacteria.D_1__Proteobacteria.D_2__Alphaproteobacteria.D_3__Reyranellales.D_4__Reyranellaceae.D_5__Reyranella		
	D_0__Bacteria.D_1__Proteobacteria.D_2__Alphaproteobacteria.D_3__Rhodobacterales.D_4__Rhodobacteraceae.__	D_0__Bacteria.D_1__Proteobacteria.D_2__Alphaproteobacteria.D_3__Rhizobiales.D_4__Devosiaceae.D_5__Devosia	D_0__Bacteria.D_1__Proteobacteria.D_2__Alphaproteobacteria.D_3__Rhizobiales.D_4__Beijerinckiaceae.D_5__28.YEA.48		
	D_0__Bacteria.D_1__Proteobacteria.D_2__Alphaproteobacteria.D_3__Sneathiellales.D_4__Sneathiellaceae.D_5__Taonella	D_0__Bacteria.D_1__Proteobacteria.D_2__Alphaproteobacteria.D_3__Rhizobiales.D_4__Hyphomicrobiaceae.D_5__Hyphomicrobium	D_0__Bacteria.D_1__Proteobacteria.D_2__Alphaproteobacteria.D_3__Rhizobiales.D_4__Beijerinckiaceae.D_5__Bosea		
	D_0__Bacteria.D_1__Proteobacteria.D_2__Alphaproteobacteria.D_3__Sphingomonadales.D_4__Sphingomonadaceae.D_5__Blastomonas	D_0__Bacteria.D_1__Proteobacteria.D_2__Alphaproteobacteria.D_3__Rhizobiales.D_4__Kaistiaceae.D_5__Kaistia	D_0__Bacteria.D_1__Proteobacteria.D_2__Alphaproteobacteria.D_3__Rhizobiales.D_4__Beijerinckiaceae.D_5__Methylobacterium		
	D_0__Bacteria.D_1__Proteobacteria.D_2__Alphaproteobacteria.D_3__Sphingomonadales.D_4__Sphingomonadaceae.D_5__Novosphingobium	D_0__Bacteria.D_1__Proteobacteria.D_2__Alphaproteobacteria.D_3__Rhizobiales.D_4__Rhizobiaceae.D_5__Allorhizobium.Neorhizobium.Pararhizobium.Rhizobium	D_0__Bacteria.D_1__Proteobacteria.D_2__Alphaproteobacteria.D_3__Rhizobiales.D_4__Beijerinckiaceae.__		
	D_0__Bacteria.D_1__Proteobacteria.D_2__Alphaproteobacteria.D_3__Sphingomonadales.D_4__Sphingomonadaceae.D_5__Sphingobium	D_0__Bacteria.D_1__Proteobacteria.D_2__Alphaproteobacteria.D_3__Rhizobiales.D_4__Rhizobiaceae.D_5__Aminobacter	D_0__Bacteria.D_1__Proteobacteria.D_2__Alphaproteobacteria.D_3__Rhizobiales.D_4__Devosiaceae.D_5__Devosia		
	D_0__Bacteria.D_1__Proteobacteria.D_2__Alphaproteobacteria.D_3__Sphingomonadales.D_4__Sphingomonadaceae.D_5__Sphingomonas	D_0__Bacteria.D_1__Proteobacteria.D_2__Alphaproteobacteria.D_3__Rhizobiales.D_4__Rhizobiaceae.D_5__Ensifer	D_0__Bacteria.D_1__Proteobacteria.D_2__Alphaproteobacteria.D_3__Rhizobiales.D_4__Hyphomicrobiaceae.D_5__Hyphomicrobium		
	D_0__Bacteria.D_1__Proteobacteria.D_2__Alphaproteobacteria.D_3__Sphingomonadales.D_4__Sphingomonadaceae.D_5__Sphingopyxis	D_0__Bacteria.D_1__Proteobacteria.D_2__Alphaproteobacteria.D_3__Rhizobiales.D_4__Rhizobiaceae.D_5__Mesorhizobium	D_0__Bacteria.D_1__Proteobacteria.D_2__Alphaproteobacteria.D_3__Rhizobiales.D_4__Kaistiaceae.D_5__Kaistia		
	D_0__Bacteria.D_1__Proteobacteria.D_2__Alphaproteobacteria.D_3__Sphingomonadales.D_4__Sphingomonadaceae.__	D_0__Bacteria.D_1__Proteobacteria.D_2__Alphaproteobacteria.D_3__Rhizobiales.D_4__Rhizobiaceae.D_5__Ochrobactrum	D_0__Bacteria.D_1__Proteobacteria.D_2__Alphaproteobacteria.D_3__Rhizobiales.D_4__Rhizobiaceae.D_5__Allorhizobium.Neorhizobium.Pararhizobium.Rhizobium		
	D_0__Bacteria.D_1__Proteobacteria.D_2__Alphaproteobacteria.__.__.__	D_0__Bacteria.D_1__Proteobacteria.D_2__Alphaproteobacteria.D_3__Rhizobiales.D_4__Rhizobiaceae.D_5__Pseudochrobactrum	D_0__Bacteria.D_1__Proteobacteria.D_2__Alphaproteobacteria.D_3__Rhizobiales.D_4__Rhizobiaceae.D_5__Aminobacter		
	D_0__Bacteria.D_1__Proteobacteria.D_2__Deltaproteobacteria.D_3__Desulfovibrionales.D_4__Desulfovibrionaceae.D_5__Desulfovibrio	D_0__Bacteria.D_1__Proteobacteria.D_2__Alphaproteobacteria.D_3__Rhizobiales.D_4__Rhizobiaceae.D_5__uncultured	D_0__Bacteria.D_1__Proteobacteria.D_2__Alphaproteobacteria.D_3__Rhizobiales.D_4__Rhizobiaceae.D_5__Ensifer		
	D_0__Bacteria.D_1__Proteobacteria.D_2__Gammaproteobacteria.D_3__Aeromonadales.D_4__Aeromonadaceae.D_5__Aeromonas	D_0__Bacteria.D_1__Proteobacteria.D_2__Alphaproteobacteria.D_3__Rhizobiales.D_4__Rhizobiaceae.__	D_0__Bacteria.D_1__Proteobacteria.D_2__Alphaproteobacteria.D_3__Rhizobiales.D_4__Rhizobiaceae.D_5__Mesorhizobium		
	D_0__Bacteria.D_1__Proteobacteria.D_2__Gammaproteobacteria.D_3__Aeromonadales.D_4__Succinivibrionaceae.D_5__Succinivibrio	D_0__Bacteria.D_1__Proteobacteria.D_2__Alphaproteobacteria.D_3__Rhizobiales.D_4__Rhizobiales.Incertae.Sedis.D_5__Nordella	D_0__Bacteria.D_1__Proteobacteria.D_2__Alphaproteobacteria.D_3__Rhizobiales.D_4__Rhizobiaceae.D_5__uncultured		
	D_0__Bacteria.D_1__Proteobacteria.D_2__Gammaproteobacteria.D_3__Aeromonadales.D_4__Succinivibrionaceae.D_5__uncultured	D_0__Bacteria.D_1__Proteobacteria.D_2__Alphaproteobacteria.D_3__Rhizobiales.D_4__Rhizobiales.Incertae.Sedis.D_5__Phreatobacter	D_0__Bacteria.D_1__Proteobacteria.D_2__Alphaproteobacteria.D_3__Rhizobiales.D_4__Rhizobiaceae.__		
	D_0__Bacteria.D_1__Proteobacteria.D_2__Gammaproteobacteria.D_3__Alteromonadales.D_4__Shewanellaceae.D_5__Shewanella	D_0__Bacteria.D_1__Proteobacteria.D_2__Alphaproteobacteria.D_3__Rhizobiales.D_4__Stappiaceae.D_5__Pannonibacter	D_0__Bacteria.D_1__Proteobacteria.D_2__Alphaproteobacteria.D_3__Rhizobiales.D_4__Xanthobacteraceae.D_5__Bradyrhizobium		
	D_0__Bacteria.D_1__Proteobacteria.D_2__Gammaproteobacteria.D_3__Betaproteobacteriales.D_4__Burkholderiaceae.Ambiguous_taxa	D_0__Bacteria.D_1__Proteobacteria.D_2__Alphaproteobacteria.D_3__Rhizobiales.D_4__Xanthobacteraceae.D_5__Bradyrhizobium	D_0__Bacteria.D_1__Proteobacteria.D_2__Alphaproteobacteria.D_3__Rhizobiales.D_4__Xanthobacteraceae.D_5__uncultured		
	D_0__Bacteria.D_1__Proteobacteria.D_2__Gammaproteobacteria.D_3__Betaproteobacteriales.D_4__Burkholderiaceae.D_5__Achromobacter	D_0__Bacteria.D_1__Proteobacteria.D_2__Alphaproteobacteria.D_3__Rhizobiales.D_4__Xanthobacteraceae.D_5__uncultured	D_0__Bacteria.D_1__Proteobacteria.D_2__Alphaproteobacteria.D_3__Rhizobiales.D_4__Xanthobacteraceae.__		
	D_0__Bacteria.D_1__Proteobacteria.D_2__Gammaproteobacteria.D_3__Betaproteobacteriales.D_4__Burkholderiaceae.D_5__Aquabacterium	D_0__Bacteria.D_1__Proteobacteria.D_2__Alphaproteobacteria.D_3__Rhizobiales.D_4__Xanthobacteraceae.__	D_0__Bacteria.D_1__Proteobacteria.D_2__Alphaproteobacteria.D_3__Rhodobacterales.D_4__Rhodobacteraceae.D_5__Defluviimonas		
	D_0__Bacteria.D_1__Proteobacteria.D_2__Gammaproteobacteria.D_3__Betaproteobacteriales.D_4__Burkholderiaceae.D_5__Burkholderia.Caballeronia.Paraburkholderia	D_0__Bacteria.D_1__Proteobacteria.D_2__Alphaproteobacteria.D_3__Rhodobacterales.D_4__Rhodobacteraceae.D_5__Gemmobacter	D_0__Bacteria.D_1__Proteobacteria.D_2__Alphaproteobacteria.D_3__Rhodobacterales.D_4__Rhodobacteraceae.D_5__Gemmobacter		
	D_0__Bacteria.D_1__Proteobacteria.D_2__Gammaproteobacteria.D_3__Betaproteobacteriales.D_4__Burkholderiaceae.D_5__Comamonas	D_0__Bacteria.D_1__Proteobacteria.D_2__Alphaproteobacteria.D_3__Rhodobacterales.D_4__Rhodobacteraceae.D_5__Ketogulonicigenium	D_0__Bacteria.D_1__Proteobacteria.D_2__Alphaproteobacteria.D_3__Rhodobacterales.D_4__Rhodobacteraceae.D_5__Ketogulonicigenium		
	D_0__Bacteria.D_1__Proteobacteria.D_2__Gammaproteobacteria.D_3__Betaproteobacteriales.D_4__Burkholderiaceae.D_5__Cupriavidus	D_0__Bacteria.D_1__Proteobacteria.D_2__Alphaproteobacteria.D_3__Rhodobacterales.D_4__Rhodobacteraceae.D_5__Paracoccus	D_0__Bacteria.D_1__Proteobacteria.D_2__Alphaproteobacteria.D_3__Rhodobacterales.D_4__Rhodobacteraceae.D_5__Paracoccus		
	D_0__Bacteria.D_1__Proteobacteria.D_2__Gammaproteobacteria.D_3__Betaproteobacteriales.D_4__Burkholderiaceae.D_5__Delftia	D_0__Bacteria.D_1__Proteobacteria.D_2__Alphaproteobacteria.D_3__Rhodobacterales.D_4__Rhodobacteraceae.D_5__Rubellimicrobium	D_0__Bacteria.D_1__Proteobacteria.D_2__Alphaproteobacteria.D_3__Rhodobacterales.D_4__Rhodobacteraceae.D_5__uncultured		
	D_0__Bacteria.D_1__Proteobacteria.D_2__Gammaproteobacteria.D_3__Betaproteobacteriales.D_4__Burkholderiaceae.D_5__Herminiimonas	D_0__Bacteria.D_1__Proteobacteria.D_2__Alphaproteobacteria.D_3__Rhodobacterales.D_4__Rhodobacteraceae.__	D_0__Bacteria.D_1__Proteobacteria.D_2__Alphaproteobacteria.D_3__Rhodobacterales.D_4__Rhodobacteraceae.__		
	D_0__Bacteria.D_1__Proteobacteria.D_2__Gammaproteobacteria.D_3__Betaproteobacteriales.D_4__Burkholderiaceae.D_5__Lautropia	D_0__Bacteria.D_1__Proteobacteria.D_2__Alphaproteobacteria.D_3__Rhodospirillales.D_4__Rhodospirillaceae.D_5__Novispirillum	D_0__Bacteria.D_1__Proteobacteria.D_2__Alphaproteobacteria.D_3__Rhodospirillales.D_4__Rhodospirillaceae.D_5__Novispirillum		
	D_0__Bacteria.D_1__Proteobacteria.D_2__Gammaproteobacteria.D_3__Betaproteobacteriales.D_4__Burkholderiaceae.D_5__Massilia	D_0__Bacteria.D_1__Proteobacteria.D_2__Alphaproteobacteria.D_3__Rickettsiales.D_4__Rickettsiaceae.D_5__Rickettsia	D_0__Bacteria.D_1__Proteobacteria.D_2__Alphaproteobacteria.D_3__Rhodospirillales.D_4__uncultured.D_5__uncultured.bacterium		
	D_0__Bacteria.D_1__Proteobacteria.D_2__Gammaproteobacteria.D_3__Betaproteobacteriales.D_4__Burkholderiaceae.D_5__Pandoraea	D_0__Bacteria.D_1__Proteobacteria.D_2__Alphaproteobacteria.D_3__Sneathiellales.D_4__Sneathiellaceae.D_5__Taonella	D_0__Bacteria.D_1__Proteobacteria.D_2__Alphaproteobacteria.D_3__Rickettsiales.D_4__Rickettsiaceae.D_5__Rickettsia		
	D_0__Bacteria.D_1__Proteobacteria.D_2__Gammaproteobacteria.D_3__Betaproteobacteriales.D_4__Burkholderiaceae.D_5__Polaromonas	D_0__Bacteria.D_1__Proteobacteria.D_2__Alphaproteobacteria.D_3__Sphingomonadales.D_4__Sphingomonadaceae.D_5__Novosphingobium	D_0__Bacteria.D_1__Proteobacteria.D_2__Alphaproteobacteria.D_3__Sneathiellales.D_4__Sneathiellaceae.D_5__Taonella		
	D_0__Bacteria.D_1__Proteobacteria.D_2__Gammaproteobacteria.D_3__Betaproteobacteriales.D_4__Burkholderiaceae.D_5__Ralstonia	D_0__Bacteria.D_1__Proteobacteria.D_2__Alphaproteobacteria.D_3__Sphingomonadales.D_4__Sphingomonadaceae.D_5__Sphingobium	D_0__Bacteria.D_1__Proteobacteria.D_2__Alphaproteobacteria.D_3__Sphingomonadales.D_4__Sphingomonadaceae.D_5__Altererythrobacter		
	D_0__Bacteria.D_1__Proteobacteria.D_2__Gammaproteobacteria.D_3__Betaproteobacteriales.D_4__Burkholderiaceae.D_5__Sutterella	D_0__Bacteria.D_1__Proteobacteria.D_2__Alphaproteobacteria.D_3__Sphingomonadales.D_4__Sphingomonadaceae.D_5__Sphingomonas	D_0__Bacteria.D_1__Proteobacteria.D_2__Alphaproteobacteria.D_3__Sphingomonadales.D_4__Sphingomonadaceae.D_5__Blastomonas		
	D_0__Bacteria.D_1__Proteobacteria.D_2__Gammaproteobacteria.D_3__Betaproteobacteriales.D_4__Burkholderiaceae.D_5__Variovorax	D_0__Bacteria.D_1__Proteobacteria.D_2__Alphaproteobacteria.D_3__Sphingomonadales.D_4__Sphingomonadaceae.D_5__Sphingopyxis	D_0__Bacteria.D_1__Proteobacteria.D_2__Alphaproteobacteria.D_3__Sphingomonadales.D_4__Sphingomonadaceae.D_5__Novosphingobium		
	D_0__Bacteria.D_1__Proteobacteria.D_2__Gammaproteobacteria.D_3__Betaproteobacteriales.D_4__Burkholderiaceae.D_5__Verticia	D_0__Bacteria.D_1__Proteobacteria.D_2__Alphaproteobacteria.D_3__Sphingomonadales.D_4__Sphingomonadaceae.__	D_0__Bacteria.D_1__Proteobacteria.D_2__Alphaproteobacteria.D_3__Sphingomonadales.D_4__Sphingomonadaceae.D_5__Sphingobium		
	D_0__Bacteria.D_1__Proteobacteria.D_2__Gammaproteobacteria.D_3__Betaproteobacteriales.D_4__Burkholderiaceae.D_5__uncultured	D_0__Bacteria.D_1__Proteobacteria.D_2__Alphaproteobacteria.__.__.__	D_0__Bacteria.D_1__Proteobacteria.D_2__Alphaproteobacteria.D_3__Sphingomonadales.D_4__Sphingomonadaceae.D_5__Sphingomonas		
	D_0__Bacteria.D_1__Proteobacteria.D_2__Gammaproteobacteria.D_3__Betaproteobacteriales.D_4__Burkholderiaceae.__	D_0__Bacteria.D_1__Proteobacteria.D_2__Deltaproteobacteria.D_3__Desulfovibrionales.D_4__Desulfovibrionaceae.D_5__Desulfovibrio	D_0__Bacteria.D_1__Proteobacteria.D_2__Alphaproteobacteria.D_3__Sphingomonadales.D_4__Sphingomonadaceae.D_5__Sphingopyxis		
	D_0__Bacteria.D_1__Proteobacteria.D_2__Gammaproteobacteria.D_3__Betaproteobacteriales.D_4__Methylophilaceae.D_5__Methylophilus	D_0__Bacteria.D_1__Proteobacteria.D_2__Gammaproteobacteria.D_3__Aeromonadales.D_4__Aeromonadaceae.D_5__Aeromonas	D_0__Bacteria.D_1__Proteobacteria.D_2__Alphaproteobacteria.D_3__Sphingomonadales.D_4__Sphingomonadaceae.__		
	D_0__Bacteria.D_1__Proteobacteria.D_2__Gammaproteobacteria.D_3__Betaproteobacteriales.D_4__Neisseriaceae.D_5__Neisseria	D_0__Bacteria.D_1__Proteobacteria.D_2__Gammaproteobacteria.D_3__Aeromonadales.D_4__Succinivibrionaceae.D_5__Succinivibrio	D_0__Bacteria.D_1__Proteobacteria.D_2__Alphaproteobacteria.__.__.__		
	D_0__Bacteria.D_1__Proteobacteria.D_2__Gammaproteobacteria.D_3__Betaproteobacteriales.D_4__Rhodocyclaceae.D_5__uncultured	D_0__Bacteria.D_1__Proteobacteria.D_2__Gammaproteobacteria.D_3__Alteromonadales.D_4__Shewanellaceae.D_5__Shewanella	D_0__Bacteria.D_1__Proteobacteria.D_2__Deltaproteobacteria.D_3__Desulfovibrionales.D_4__Desulfovibrionaceae.D_5__Bilophila		
	D_0__Bacteria.D_1__Proteobacteria.D_2__Gammaproteobacteria.D_3__Cellvibrionales.D_4__Cellvibrionaceae.D_5__Cellvibrio	D_0__Bacteria.D_1__Proteobacteria.D_2__Gammaproteobacteria.D_3__Betaproteobacteriales.D_4__Burkholderiaceae.Ambiguous_taxa	D_0__Bacteria.D_1__Proteobacteria.D_2__Deltaproteobacteria.D_3__Desulfovibrionales.D_4__Desulfovibrionaceae.D_5__Desulfovibrio		
	D_0__Bacteria.D_1__Proteobacteria.D_2__Gammaproteobacteria.D_3__Enterobacteriales.D_4__Enterobacteriaceae.D_5__Citrobacter	D_0__Bacteria.D_1__Proteobacteria.D_2__Gammaproteobacteria.D_3__Betaproteobacteriales.D_4__Burkholderiaceae.D_5__Achromobacter	D_0__Bacteria.D_1__Proteobacteria.D_2__Deltaproteobacteria.D_3__Desulfovibrionales.D_4__Desulfovibrionaceae.D_5__uncultured		
	D_0__Bacteria.D_1__Proteobacteria.D_2__Gammaproteobacteria.D_3__Enterobacteriales.D_4__Enterobacteriaceae.D_5__Cronobacter	D_0__Bacteria.D_1__Proteobacteria.D_2__Gammaproteobacteria.D_3__Betaproteobacteriales.D_4__Burkholderiaceae.D_5__Acidovorax	D_0__Bacteria.D_1__Proteobacteria.D_2__Gammaproteobacteria.D_3__Aeromonadales.D_4__Aeromonadaceae.D_5__Aeromonas		
	D_0__Bacteria.D_1__Proteobacteria.D_2__Gammaproteobacteria.D_3__Enterobacteriales.D_4__Enterobacteriaceae.D_5__Enterobacter	D_0__Bacteria.D_1__Proteobacteria.D_2__Gammaproteobacteria.D_3__Betaproteobacteriales.D_4__Burkholderiaceae.D_5__Aquabacterium	D_0__Bacteria.D_1__Proteobacteria.D_2__Gammaproteobacteria.D_3__Aeromonadales.D_4__Succinivibrionaceae.D_5__Succinivibrio		
	D_0__Bacteria.D_1__Proteobacteria.D_2__Gammaproteobacteria.D_3__Enterobacteriales.D_4__Enterobacteriaceae.D_5__Escherichia.Shigella	D_0__Bacteria.D_1__Proteobacteria.D_2__Gammaproteobacteria.D_3__Betaproteobacteriales.D_4__Burkholderiaceae.D_5__Burkholderia.Caballeronia.Paraburkholderia	D_0__Bacteria.D_1__Proteobacteria.D_2__Gammaproteobacteria.D_3__Aeromonadales.D_4__Succinivibrionaceae.D_5__uncultured		
	D_0__Bacteria.D_1__Proteobacteria.D_2__Gammaproteobacteria.D_3__Enterobacteriales.D_4__Enterobacteriaceae.D_5__Klebsiella	D_0__Bacteria.D_1__Proteobacteria.D_2__Gammaproteobacteria.D_3__Betaproteobacteriales.D_4__Burkholderiaceae.D_5__Comamonas	D_0__Bacteria.D_1__Proteobacteria.D_2__Gammaproteobacteria.D_3__Aeromonadales.D_4__Succinivibrionaceae.__		
	D_0__Bacteria.D_1__Proteobacteria.D_2__Gammaproteobacteria.D_3__Enterobacteriales.D_4__Enterobacteriaceae.D_5__Kosakonia	D_0__Bacteria.D_1__Proteobacteria.D_2__Gammaproteobacteria.D_3__Betaproteobacteriales.D_4__Burkholderiaceae.D_5__Cupriavidus	D_0__Bacteria.D_1__Proteobacteria.D_2__Gammaproteobacteria.D_3__Alteromonadales.D_4__Shewanellaceae.D_5__Shewanella		
	D_0__Bacteria.D_1__Proteobacteria.D_2__Gammaproteobacteria.D_3__Enterobacteriales.D_4__Enterobacteriaceae.D_5__Pantoea	D_0__Bacteria.D_1__Proteobacteria.D_2__Gammaproteobacteria.D_3__Betaproteobacteriales.D_4__Burkholderiaceae.D_5__Curvibacter	D_0__Bacteria.D_1__Proteobacteria.D_2__Gammaproteobacteria.D_3__Betaproteobacteriales.D_4__Burkholderiaceae.D_5__Achromobacter		
	D_0__Bacteria.D_1__Proteobacteria.D_2__Gammaproteobacteria.D_3__Enterobacteriales.D_4__Enterobacteriaceae.D_5__Serratia	D_0__Bacteria.D_1__Proteobacteria.D_2__Gammaproteobacteria.D_3__Betaproteobacteriales.D_4__Burkholderiaceae.D_5__Delftia	D_0__Bacteria.D_1__Proteobacteria.D_2__Gammaproteobacteria.D_3__Betaproteobacteriales.D_4__Burkholderiaceae.D_5__Acidovorax		
	D_0__Bacteria.D_1__Proteobacteria.D_2__Gammaproteobacteria.D_3__Enterobacteriales.D_4__Enterobacteriaceae.D_5__Yersinia	D_0__Bacteria.D_1__Proteobacteria.D_2__Gammaproteobacteria.D_3__Betaproteobacteriales.D_4__Burkholderiaceae.D_5__Diaphorobacter	D_0__Bacteria.D_1__Proteobacteria.D_2__Gammaproteobacteria.D_3__Betaproteobacteriales.D_4__Burkholderiaceae.D_5__Aquabacterium		
	D_0__Bacteria.D_1__Proteobacteria.D_2__Gammaproteobacteria.D_3__Enterobacteriales.D_4__Enterobacteriaceae.__	D_0__Bacteria.D_1__Proteobacteria.D_2__Gammaproteobacteria.D_3__Betaproteobacteriales.D_4__Burkholderiaceae.D_5__Limnobacter	D_0__Bacteria.D_1__Proteobacteria.D_2__Gammaproteobacteria.D_3__Betaproteobacteriales.D_4__Burkholderiaceae.D_5__Burkholderia.Caballeronia.Paraburkholderia		
	D_0__Bacteria.D_1__Proteobacteria.D_2__Gammaproteobacteria.D_3__Pasteurellales.D_4__Pasteurellaceae.D_5__Actinobacillus	D_0__Bacteria.D_1__Proteobacteria.D_2__Gammaproteobacteria.D_3__Betaproteobacteriales.D_4__Burkholderiaceae.D_5__Massilia	D_0__Bacteria.D_1__Proteobacteria.D_2__Gammaproteobacteria.D_3__Betaproteobacteriales.D_4__Burkholderiaceae.D_5__Comamonas		
	D_0__Bacteria.D_1__Proteobacteria.D_2__Gammaproteobacteria.D_3__Pasteurellales.D_4__Pasteurellaceae.D_5__Haemophilus	D_0__Bacteria.D_1__Proteobacteria.D_2__Gammaproteobacteria.D_3__Betaproteobacteriales.D_4__Burkholderiaceae.D_5__Methylibium	D_0__Bacteria.D_1__Proteobacteria.D_2__Gammaproteobacteria.D_3__Betaproteobacteriales.D_4__Burkholderiaceae.D_5__Cupriavidus		
	D_0__Bacteria.D_1__Proteobacteria.D_2__Gammaproteobacteria.D_3__Pasteurellales.D_4__Pasteurellaceae.__	D_0__Bacteria.D_1__Proteobacteria.D_2__Gammaproteobacteria.D_3__Betaproteobacteriales.D_4__Burkholderiaceae.D_5__Paenalcaligenes	D_0__Bacteria.D_1__Proteobacteria.D_2__Gammaproteobacteria.D_3__Betaproteobacteriales.D_4__Burkholderiaceae.D_5__Curvibacter		
	D_0__Bacteria.D_1__Proteobacteria.D_2__Gammaproteobacteria.D_3__Pseudomonadales.D_4__Moraxellaceae.D_5__Acinetobacter	D_0__Bacteria.D_1__Proteobacteria.D_2__Gammaproteobacteria.D_3__Betaproteobacteriales.D_4__Burkholderiaceae.D_5__Pelomonas	D_0__Bacteria.D_1__Proteobacteria.D_2__Gammaproteobacteria.D_3__Betaproteobacteriales.D_4__Burkholderiaceae.D_5__Delftia		
	D_0__Bacteria.D_1__Proteobacteria.D_2__Gammaproteobacteria.D_3__Pseudomonadales.D_4__Moraxellaceae.D_5__Alkanindiges	D_0__Bacteria.D_1__Proteobacteria.D_2__Gammaproteobacteria.D_3__Betaproteobacteriales.D_4__Burkholderiaceae.D_5__Polaromonas	D_0__Bacteria.D_1__Proteobacteria.D_2__Gammaproteobacteria.D_3__Betaproteobacteriales.D_4__Burkholderiaceae.D_5__Diaphorobacter		
	D_0__Bacteria.D_1__Proteobacteria.D_2__Gammaproteobacteria.D_3__Pseudomonadales.D_4__Pseudomonadaceae.D_5__Pseudomonas	D_0__Bacteria.D_1__Proteobacteria.D_2__Gammaproteobacteria.D_3__Betaproteobacteriales.D_4__Burkholderiaceae.D_5__Pseudacidovorax	D_0__Bacteria.D_1__Proteobacteria.D_2__Gammaproteobacteria.D_3__Betaproteobacteriales.D_4__Burkholderiaceae.D_5__Duganella		
	D_0__Bacteria.D_1__Proteobacteria.D_2__Gammaproteobacteria.D_3__Xanthomonadales.D_4__Rhodanobacteraceae.D_5__Dokdonella	D_0__Bacteria.D_1__Proteobacteria.D_2__Gammaproteobacteria.D_3__Betaproteobacteriales.D_4__Burkholderiaceae.D_5__Ralstonia	D_0__Bacteria.D_1__Proteobacteria.D_2__Gammaproteobacteria.D_3__Betaproteobacteriales.D_4__Burkholderiaceae.D_5__Herbaspirillum		
	D_0__Bacteria.D_1__Proteobacteria.D_2__Gammaproteobacteria.D_3__Xanthomonadales.D_4__Xanthomonadaceae.D_5__Luteimonas	D_0__Bacteria.D_1__Proteobacteria.D_2__Gammaproteobacteria.D_3__Betaproteobacteriales.D_4__Burkholderiaceae.D_5__Roseateles	D_0__Bacteria.D_1__Proteobacteria.D_2__Gammaproteobacteria.D_3__Betaproteobacteriales.D_4__Burkholderiaceae.D_5__Herminiimonas		
	D_0__Bacteria.D_1__Proteobacteria.D_2__Gammaproteobacteria.D_3__Xanthomonadales.D_4__Xanthomonadaceae.D_5__Pseudoxanthomonas	D_0__Bacteria.D_1__Proteobacteria.D_2__Gammaproteobacteria.D_3__Betaproteobacteriales.D_4__Burkholderiaceae.D_5__Sutterella	D_0__Bacteria.D_1__Proteobacteria.D_2__Gammaproteobacteria.D_3__Betaproteobacteriales.D_4__Burkholderiaceae.D_5__Lautropia		
	D_0__Bacteria.D_1__Proteobacteria.D_2__Gammaproteobacteria.D_3__Xanthomonadales.D_4__Xanthomonadaceae.D_5__Stenotrophomonas	D_0__Bacteria.D_1__Proteobacteria.D_2__Gammaproteobacteria.D_3__Betaproteobacteriales.D_4__Burkholderiaceae.D_5__Variovorax	D_0__Bacteria.D_1__Proteobacteria.D_2__Gammaproteobacteria.D_3__Betaproteobacteriales.D_4__Burkholderiaceae.D_5__Massilia		

OTUs present among HC	OTUs present among Cases_Day1	OTUs present among Cases_Day15	OTUs present among Cases_Day30	OTUs present among only Cases_Day30_A	OTUs present among only Cases_Day30_B
	D_0__Bacteria.D_1__Proteobacteria.D_2__Gammaproteobacteria.D_3__Xanthomonadales.D_4__Xanthomonadaceae.__	D_0__Bacteria.D_1__Proteobacteria.D_2__Gammaproteobacteria.D_3__Betaproteobacteriales.D_4__Burkholderiaceae.D_5__Verticia	D_0__Bacteria.D_1__Proteobacteria.D_2__Gammaproteobacteria.D_3__Betaproteobacteriales.D_4__Burkholderiaceae.D_5__Noviherbaspirillum		
	D_0__Bacteria.D_1__Proteobacteria.D_2__Gammaproteobacteria.__.__.__	D_0__Bacteria.D_1__Proteobacteria.D_2__Gammaproteobacteria.D_3__Betaproteobacteriales.D_4__Burkholderiaceae.D_5__uncultured	D_0__Bacteria.D_1__Proteobacteria.D_2__Gammaproteobacteria.D_3__Betaproteobacteriales.D_4__Burkholderiaceae.D_5__Parasutterella		
	D_0__Bacteria.D_1__Spirochaetes.D_2__Spirochaetia.D_3__Spirochaetales.D_4__Spirochaetaceae.D_5__Treponema.2	D_0__Bacteria.D_1__Proteobacteria.D_2__Gammaproteobacteria.D_3__Betaproteobacteriales.D_4__Burkholderiaceae.__	D_0__Bacteria.D_1__Proteobacteria.D_2__Gammaproteobacteria.D_3__Betaproteobacteriales.D_4__Burkholderiaceae.D_5__Pelomonas		
	D_0__Bacteria.D_1__Tenericutes.D_2__Mollicutes.D_3__Mycoplasmatales.D_4__Mycoplasmataceae.D_5__Mycoplasma	D_0__Bacteria.D_1__Proteobacteria.D_2__Gammaproteobacteria.D_3__Betaproteobacteriales.D_4__Chitinimonadaceae.D_5__Chitinimonas	D_0__Bacteria.D_1__Proteobacteria.D_2__Gammaproteobacteria.D_3__Betaproteobacteriales.D_4__Burkholderiaceae.D_5__Pigmentiphaga		
	D_0__Bacteria.D_1__Tenericutes.D_2__Mollicutes.D_3__Mycoplasmatales.D_4__Mycoplasmataceae.D_5__Ureaplasma	D_0__Bacteria.D_1__Proteobacteria.D_2__Gammaproteobacteria.D_3__Betaproteobacteriales.D_4__Chromobacteriaceae.D_5__Chromobacterium	D_0__Bacteria.D_1__Proteobacteria.D_2__Gammaproteobacteria.D_3__Betaproteobacteriales.D_4__Burkholderiaceae.D_5__Pseudacidovorax		
	D_0__Bacteria.D_1__Verrucomicrobia.D_2__Verrucomicrobiae.D_3__Verrucomicrobiales.D_4__Akkermansiaceae.D_5__Akkermansia	D_0__Bacteria.D_1__Proteobacteria.D_2__Gammaproteobacteria.D_3__Betaproteobacteriales.D_4__Neisseriaceae.D_5__Neisseria	D_0__Bacteria.D_1__Proteobacteria.D_2__Gammaproteobacteria.D_3__Betaproteobacteriales.D_4__Burkholderiaceae.D_5__Ralstonia		
	D_0__Bacteria.D_1__Verrucomicrobia.D_2__Verrucomicrobiae.D_3__Verrucomicrobiales.D_4__Rubritaleaceae.D_5__Luteolibacter	D_0__Bacteria.D_1__Proteobacteria.D_2__Gammaproteobacteria.D_3__Betaproteobacteriales.__.__	D_0__Bacteria.D_1__Proteobacteria.D_2__Gammaproteobacteria.D_3__Betaproteobacteriales.D_4__Burkholderiaceae.D_5__Ramlibacter		
		D_0__Bacteria.D_1__Proteobacteria.D_2__Gammaproteobacteria.D_3__Cellvibrionales.D_4__Cellvibrionaceae.D_5__Cellvibrio	D_0__Bacteria.D_1__Proteobacteria.D_2__Gammaproteobacteria.D_3__Betaproteobacteriales.D_4__Burkholderiaceae.D_5__Roseateles		
		D_0__Bacteria.D_1__Proteobacteria.D_2__Gammaproteobacteria.D_3__Enterobacteriales.D_4__Enterobacteriaceae.D_5__Citrobacter	D_0__Bacteria.D_1__Proteobacteria.D_2__Gammaproteobacteria.D_3__Betaproteobacteriales.D_4__Burkholderiaceae.D_5__Sutterella		
		D_0__Bacteria.D_1__Proteobacteria.D_2__Gammaproteobacteria.D_3__Enterobacteriales.D_4__Enterobacteriaceae.D_5__Cronobacter	D_0__Bacteria.D_1__Proteobacteria.D_2__Gammaproteobacteria.D_3__Betaproteobacteriales.D_4__Burkholderiaceae.D_5__Undibacterium		
		D_0__Bacteria.D_1__Proteobacteria.D_2__Gammaproteobacteria.D_3__Enterobacteriales.D_4__Enterobacteriaceae.D_5__Enterobacter	D_0__Bacteria.D_1__Proteobacteria.D_2__Gammaproteobacteria.D_3__Betaproteobacteriales.D_4__Burkholderiaceae.D_5__Variovorax		
		D_0__Bacteria.D_1__Proteobacteria.D_2__Gammaproteobacteria.D_3__Enterobacteriales.D_4__Enterobacteriaceae.D_5__Escherichia.Shigella	D_0__Bacteria.D_1__Proteobacteria.D_2__Gammaproteobacteria.D_3__Betaproteobacteriales.D_4__Burkholderiaceae.D_5__Verticia		
		D_0__Bacteria.D_1__Proteobacteria.D_2__Gammaproteobacteria.D_3__Enterobacteriales.D_4__Enterobacteriaceae.D_5__Klebsiella	D_0__Bacteria.D_1__Proteobacteria.D_2__Gammaproteobacteria.D_3__Betaproteobacteriales.D_4__Burkholderiaceae.D_5__Xenophilus		
		D_0__Bacteria.D_1__Proteobacteria.D_2__Gammaproteobacteria.D_3__Enterobacteriales.D_4__Enterobacteriaceae.D_5__Kosakonia	D_0__Bacteria.D_1__Proteobacteria.D_2__Gammaproteobacteria.D_3__Betaproteobacteriales.D_4__Burkholderiaceae.D_5__uncultured		
		D_0__Bacteria.D_1__Proteobacteria.D_2__Gammaproteobacteria.D_3__Enterobacteriales.D_4__Enterobacteriaceae.D_5__Pantoea	D_0__Bacteria.D_1__Proteobacteria.D_2__Gammaproteobacteria.D_3__Betaproteobacteriales.D_4__Burkholderiaceae.__		
		D_0__Bacteria.D_1__Proteobacteria.D_2__Gammaproteobacteria.D_3__Enterobacteriales.D_4__Enterobacteriaceae.D_5__Proteus	D_0__Bacteria.D_1__Proteobacteria.D_2__Gammaproteobacteria.D_3__Betaproteobacteriales.D_4__Chromobacteriaceae.D_5__Chromobacterium		
		D_0__Bacteria.D_1__Proteobacteria.D_2__Gammaproteobacteria.D_3__Enterobacteriales.D_4__Enterobacteriaceae.D_5__Serratia	D_0__Bacteria.D_1__Proteobacteria.D_2__Gammaproteobacteria.D_3__Betaproteobacteriales.D_4__Methylophilaceae.D_5__Methylophilus		
		D_0__Bacteria.D_1__Proteobacteria.D_2__Gammaproteobacteria.D_3__Enterobacteriales.D_4__Enterobacteriaceae.D_5__Yersinia	D_0__Bacteria.D_1__Proteobacteria.D_2__Gammaproteobacteria.D_3__Betaproteobacteriales.D_4__Neisseriaceae.D_5__Neisseria		
		D_0__Bacteria.D_1__Proteobacteria.D_2__Gammaproteobacteria.D_3__Enterobacteriales.D_4__Enterobacteriaceae.__	D_0__Bacteria.D_1__Proteobacteria.D_2__Gammaproteobacteria.D_3__Betaproteobacteriales.D_4__Rhodocyclaceae.D_5__C39		
		D_0__Bacteria.D_1__Proteobacteria.D_2__Gammaproteobacteria.D_3__Gammaproteobacteria.Incertae.Sedis.D_4__Unknown.Family.D_5__Candidatus.Berkiella	D_0__Bacteria.D_1__Proteobacteria.D_2__Gammaproteobacteria.D_3__Betaproteobacteriales.D_4__Rhodocyclaceae.D_5__Methyloversatilis		
		D_0__Bacteria.D_1__Proteobacteria.D_2__Gammaproteobacteria.D_3__Pasteurellales.D_4__Pasteurellaceae.D_5__Actinobacillus	D_0__Bacteria.D_1__Proteobacteria.D_2__Gammaproteobacteria.D_3__Betaproteobacteriales.__.__		
		D_0__Bacteria.D_1__Proteobacteria.D_2__Gammaproteobacteria.D_3__Pasteurellales.D_4__Pasteurellaceae.D_5__Aggregatibacter	D_0__Bacteria.D_1__Proteobacteria.D_2__Gammaproteobacteria.D_3__Cellvibrionales.D_4__Cellvibrionaceae.D_5__Cellvibrio		
		D_0__Bacteria.D_1__Proteobacteria.D_2__Gammaproteobacteria.D_3__Pasteurellales.D_4__Pasteurellaceae.D_5__Haemophilus	D_0__Bacteria.D_1__Proteobacteria.D_2__Gammaproteobacteria.D_3__Enterobacteriales.D_4__Enterobacteriaceae.D_5__Buttiauxella		
		D_0__Bacteria.D_1__Proteobacteria.D_2__Gammaproteobacteria.D_3__Pasteurellales.D_4__Pasteurellaceae.__	D_0__Bacteria.D_1__Proteobacteria.D_2__Gammaproteobacteria.D_3__Enterobacteriales.D_4__Enterobacteriaceae.D_5__Citrobacter		
		D_0__Bacteria.D_1__Proteobacteria.D_2__Gammaproteobacteria.D_3__Pseudomonadales.D_4__Moraxellaceae.D_5__Acinetobacter	D_0__Bacteria.D_1__Proteobacteria.D_2__Gammaproteobacteria.D_3__Enterobacteriales.D_4__Enterobacteriaceae.D_5__Cronobacter		
		D_0__Bacteria.D_1__Proteobacteria.D_2__Gammaproteobacteria.D_3__Pseudomonadales.D_4__Moraxellaceae.D_5__Alkanindiges	D_0__Bacteria.D_1__Proteobacteria.D_2__Gammaproteobacteria.D_3__Enterobacteriales.D_4__Enterobacteriaceae.D_5__Enterobacter		
		D_0__Bacteria.D_1__Proteobacteria.D_2__Gammaproteobacteria.D_3__Pseudomonadales.D_4__Moraxellaceae.D_5__Enhydrobacter	D_0__Bacteria.D_1__Proteobacteria.D_2__Gammaproteobacteria.D_3__Enterobacteriales.D_4__Enterobacteriaceae.D_5__Escherichia.Shigella		
		D_0__Bacteria.D_1__Proteobacteria.D_2__Gammaproteobacteria.D_3__Pseudomonadales.D_4__Pseudomonadaceae.D_5__Pseudomonas	D_0__Bacteria.D_1__Proteobacteria.D_2__Gammaproteobacteria.D_3__Enterobacteriales.D_4__Enterobacteriaceae.D_5__Klebsiella		
		D_0__Bacteria.D_1__Proteobacteria.D_2__Gammaproteobacteria.D_3__Xanthomonadales.D_4__Rhodanobacteraceae.D_5__Dokdonella	D_0__Bacteria.D_1__Proteobacteria.D_2__Gammaproteobacteria.D_3__Enterobacteriales.D_4__Enterobacteriaceae.D_5__Kosakonia		
		D_0__Bacteria.D_1__Proteobacteria.D_2__Gammaproteobacteria.D_3__Xanthomonadales.D_4__Xanthomonadaceae.D_5__Pseudoxanthomonas	D_0__Bacteria.D_1__Proteobacteria.D_2__Gammaproteobacteria.D_3__Enterobacteriales.D_4__Enterobacteriaceae.D_5__Pantoea		
		D_0__Bacteria.D_1__Proteobacteria.D_2__Gammaproteobacteria.D_3__Xanthomonadales.D_4__Xanthomonadaceae.D_5__Stenotrophomonas	D_0__Bacteria.D_1__Proteobacteria.D_2__Gammaproteobacteria.D_3__Enterobacteriales.D_4__Enterobacteriaceae.D_5__Proteus		
		D_0__Bacteria.D_1__Proteobacteria.D_2__Gammaproteobacteria.D_3__Xanthomonadales.D_4__Xanthomonadaceae.__	D_0__Bacteria.D_1__Proteobacteria.D_2__Gammaproteobacteria.D_3__Enterobacteriales.D_4__Enterobacteriaceae.D_5__Rahnella		
		D_0__Bacteria.D_1__Proteobacteria.D_2__Gammaproteobacteria.__.__.__	D_0__Bacteria.D_1__Proteobacteria.D_2__Gammaproteobacteria.D_3__Enterobacteriales.D_4__Enterobacteriaceae.D_5__Serratia		
		D_0__Bacteria.D_1__Proteobacteria.__.__.__.__	D_0__Bacteria.D_1__Proteobacteria.D_2__Gammaproteobacteria.D_3__Enterobacteriales.D_4__Enterobacteriaceae.D_5__Yersinia		
		D_0__Bacteria.D_1__Spirochaetes.D_2__Spirochaetia.D_3__Spirochaetales.D_4__Spirochaetaceae.D_5__Treponema.2	D_0__Bacteria.D_1__Proteobacteria.D_2__Gammaproteobacteria.D_3__Enterobacteriales.D_4__Enterobacteriaceae.__		
		D_0__Bacteria.D_1__Synergistetes.D_2__Synergistia.D_3__Synergistales.D_4__Synergistaceae.D_5__Fretibacterium	D_0__Bacteria.D_1__Proteobacteria.D_2__Gammaproteobacteria.D_3__Gammaproteobacteria.Incertae.Sedis.D_4__Unknown.Family.D_5__uncultured.bacterium		
		D_0__Bacteria.D_1__Synergistetes.D_2__Synergistia.D_3__Synergistales.D_4__Synergistaceae.D_5__Jonquetella	D_0__Bacteria.D_1__Proteobacteria.D_2__Gammaproteobacteria.D_3__Pasteurellales.D_4__Pasteurellaceae.D_5__Actinobacillus		
		D_0__Bacteria.D_1__Tenericutes.D_2__Mollicutes.D_3__Mycoplasmatales.D_4__Mycoplasmataceae.D_5__Mycoplasma	D_0__Bacteria.D_1__Proteobacteria.D_2__Gammaproteobacteria.D_3__Pasteurellales.D_4__Pasteurellaceae.D_5__Aggregatibacter		
		D_0__Bacteria.D_1__Tenericutes.D_2__Mollicutes.D_3__Mycoplasmatales.D_4__Mycoplasmataceae.D_5__Ureaplasma	D_0__Bacteria.D_1__Proteobacteria.D_2__Gammaproteobacteria.D_3__Pasteurellales.D_4__Pasteurellaceae.D_5__Haemophilus		
		D_0__Bacteria.D_1__Verrucomicrobia.D_2__Verrucomicrobiae.D_3__Verrucomicrobiales.D_4__Akkermansiaceae.D_5__Akkermansia	D_0__Bacteria.D_1__Proteobacteria.D_2__Gammaproteobacteria.D_3__Pseudomonadales.D_4__Moraxellaceae.D_5__Acinetobacter		
		D_0__Bacteria.D_1__Verrucomicrobia.D_2__Verrucomicrobiae.D_3__Verrucomicrobiales.D_4__Rubritaleaceae.D_5__Luteolibacter	D_0__Bacteria.D_1__Proteobacteria.D_2__Gammaproteobacteria.D_3__Pseudomonadales.D_4__Moraxellaceae.D_5__Alkanindiges		
		D_0__Bacteria.D_1__Verrucomicrobia.D_2__Verrucomicrobiae.D_3__Verrucomicrobiales.D_4__Verrucomicrobiaceae.D_5__Verrucomicrobium	D_0__Bacteria.D_1__Proteobacteria.D_2__Gammaproteobacteria.D_3__Pseudomonadales.D_4__Moraxellaceae.D_5__Enhydrobacter		
		D_0__Bacteria.__.__.__.__.__	D_0__Bacteria.D_1__Proteobacteria.D_2__Gammaproteobacteria.D_3__Pseudomonadales.D_4__Moraxellaceae.D_5__uncultured		
			D_0__Bacteria.D_1__Proteobacteria.D_2__Gammaproteobacteria.D_3__Pseudomonadales.D_4__Pseudomonadaceae.D_5__Pseudomonas		
			D_0__Bacteria.D_1__Proteobacteria.D_2__Gammaproteobacteria.D_3__Salinisphaerales.D_4__Solimonadaceae.D_5__uncultured		
			D_0__Bacteria.D_1__Proteobacteria.D_2__Gammaproteobacteria.D_3__Xanthomonadales.D_4__Rhodanobacteraceae.D_5__Dokdonella		
			D_0__Bacteria.D_1__Proteobacteria.D_2__Gammaproteobacteria.D_3__Xanthomonadales.D_4__Xanthomonadaceae.D_5__Pseudoxanthomonas		
			D_0__Bacteria.D_1__Proteobacteria.D_2__Gammaproteobacteria.D_3__Xanthomonadales.D_4__Xanthomonadaceae.D_5__Stenotrophomonas		
			D_0__Bacteria.D_1__Proteobacteria.D_2__Gammaproteobacteria.__.__.__		
			D_0__Bacteria.D_1__Proteobacteria.__.__.__.__		
			D_0__Bacteria.D_1__Spirochaetes.D_2__Spirochaetia.D_3__Spirochaetales.D_4__Spirochaetaceae.D_5__Treponema.2		
			D_0__Bacteria.D_1__Tenericutes.D_2__Mollicutes.D_3__Mycoplasmatales.D_4__Mycoplasmataceae.D_5__Mycoplasma		
			D_0__Bacteria.D_1__Tenericutes.D_2__Mollicutes.D_3__Mycoplasmatales.D_4__Mycoplasmataceae.D_5__Ureaplasma		
			D_0__Bacteria.D_1__Verrucomicrobia.D_2__Verrucomicrobiae.D_3__Verrucomicrobiales.D_4__Akkermansiaceae.D_5__Akkermansia		
			D_0__Bacteria.D_1__Verrucomicrobia.D_2__Verrucomicrobiae.D_3__Verrucomicrobiales.D_4__Rubritaleaceae.D_5__Luteolibacter		
			D_0__Bacteria.__.__.__.__.__		

Supplementary Table 4: The OTUs which have strong evidence to be causal or have already shown to be strongly associated with lung infection

Genus	LDA Score (Control vs Case)
Acenetobactor	-4.6
Chryseobacterium	-4.4
Stenotrophomonas	-4.3
Serratia	-3.9
Citrobacter	-3.8
Enterobacter	-5.1
Bifidobacterium	-2.4
Blautia	-2.4
Streptococcus	5.2
Klebsiella	5.1
Haemophilus	5.1
Velionella	4.8
Prostheotobactor	3.7
Granulcatella	3.5
Neisseria	3.3
Rummeliibacillus	3.3
Marmoricola	3.2
Rothia	3.2

Note of Supplementary Table 4:

The proportional rise of the Genus likethat includes Acenetobactor (LDA Score: 4.6), Chryseobacterium (LDA Score: 4.4), Stenotrophomonas (LDA Score:4.3), Serratia (LDA Score:3.9), Citrobacter (LDA Score: 3.8) Enterobacter (LDA Score: 5.1), Bifidobacterium and Blautia (LDA Score:2.4) were in significantly higher proportion among the associated with cases and. The genus like Streptococcus (LDA Score: 5.2), Klebsiella(LDA Score: 5.01), Haemophilus (LDA Score:5.01), Velionella (LDA Score: 4.8), Prostheotobactor, Granulcatella, Neisseria, Rummeliibacillus, Marmoricola and Rothia (LDA Score ≥3) were significantly depleted among the cases compared to HC; (For a complete list see Fig.4B and S_F_Table 1). We further computed the LDA between cases

Day 1 and HC (Fig.4C) and cases Day 15 and HC (Fig4D) with HC. Along with the genus Acenetobactor, Chryseobacterium, Stenotrophomonas (LDA Score:4.3), Citrobacter (LDA Score: 3.8) Enterobacter we found the genus Senegalimassilia, Kosakonia, Libanicoccus and Salkia significantly elevated among cases with LDA Score>3. Using LDA, we further looked for OTUs which differentiate categorized our cases with Group A with HC(Fig.4E) and Group B with HC(Fig.4F) groupwise who become RT-PCR positive till Day 30 and RT-PCR negative at Day 30, respectively and done LDA with HC separately. The data reveals and found that the genus Parabacteroides, Chryseobacterium and Parasutterella are significantly associated with cases(LDA Score>3) however the genus Bifidobacterium, Aerococcus, Gordonibacter, Lachnoclostridium, Delfia, Muribaculum, jeotgalicoccus and Duboscilla are significantly associate with Group A compared to HC.
