# Supplementary material for: Temporal dynamics of oropharyngeal microbiome among SARS-CoV-2 patients reveals continued dysbiosis even after Viral Clearance
Source: NPJ Biofilms Microbiomes. 2022 Aug 24;8:67. doi: 10.1038/s41522-022-00330-y (PMC9400563; doi:10.1038/s41522-022-00330-y)
Supplement: Supplementary file 5 — Reporting Summary [file 41522_2022_330_MOESM5_ESM.pdf]

nature portfolio

Corresponding author(s): Analabha Basu

Last updated by author(s): Jul 7, 2022

Reporting Summary

Nature Portfolio wishes to improve the reproducibility of the work that we publish. This form provides structure for consistency and transparency in reporting. For further information on Nature Portfolio policies, see our Editorial Policies and the Editorial Policy Checklist.

Statistics

For all statistical analyses, confirm that the following items are present in the figure legend, table legend, main text, or Methods section.

n/a	Confirmed	
☐	☒	The exact sample size (*n*) for each experimental group/condition, given as a discrete number and unit of measurement
☐	☒	A statement on whether measurements were taken from distinct samples or whether the same sample was measured repeatedly
☐	☒	The statistical test(s) used AND whether they are one- or two-sided<br>*Only common tests should be described solely by name; describe more complex techniques in the Methods section.*
☒	☐	A description of all covariates tested
☐	☒	A description of any assumptions or corrections, such as tests of normality and adjustment for multiple comparisons
☐	☒	A full description of the statistical parameters including central tendency (e.g. means) or other basic estimates (e.g. regression coefficient) AND variation (e.g. standard deviation) or associated estimates of uncertainty (e.g. confidence intervals)
☐	☒	For null hypothesis testing, the test statistic (e.g. *F*, *t*, *r*) with confidence intervals, effect sizes, degrees of freedom and *P* value noted<br>*Give P values as exact values whenever suitable.*
☐	☒	For Bayesian analysis, information on the choice of priors and Markov chain Monte Carlo settings
☒	☐	For hierarchical and complex designs, identification of the appropriate level for tests and full reporting of outcomes
☒	☐	Estimates of effect sizes (e.g. Cohen's *d*, Pearson's *r*), indicating how they were calculated

Our web collection on statistics for biologists contains articles on many of the points above.

Software and code

Policy information about availability of computer code

Data collection	No software used
Data analysis	QIIME, Version 2.0 and R, version 4.0.5.

For manuscripts utilizing custom algorithms or software that are central to the research but not yet described in published literature, software must be made available to editors and reviewers. We strongly encourage code deposition in a community repository (e.g. GitHub). See the Nature Portfolio guidelines for submitting code & software for further information.

Data

Policy information about availability of data

All manuscripts must include a data availability statement. This statement should provide the following information, where applicable:

- Accession codes, unique identifiers, or web links for publicly available datasets
- A description of any restrictions on data availability
- For clinical datasets or third party data, please ensure that the statement adheres to our policy

The raw data and Meta data are available in the following link: https://www.ncbi.nlm.nih.gov/bioproject/PRJNA850148

Field-specific reporting

Please select the one below that is the best fit for your research. If you are not sure, read the appropriate sections before making your selection.

☒ Life sciences ☐ Behavioural & social sciences ☐ Ecological, evolutionary & environmental sciences

For a reference copy of the document with all sections, see nature.com/documents/nr-reporting-summary-flat.pdf

Life sciences study design

All studies must disclose on these points even when the disclosure is negative.

Sample size	We did not do a formal power calculation for sample size estimation. Because the effect size is unknown, a proper power calculation is not possible.<br>Our's is a longitudinal study with three time points. We prioritized detailed follow up of study participants, which was difficult, rather increasing number of samples.
Data exclusions	No data was excluded.
Replication	This study is not a replication of any previous study.<br>The study aims for temporal variation of microbial dysbiosis after SARS-COV2 infection. Hence, we have collected samples at three time points. But we have not replicated this entire process in another setup or in another population.
Randomization	This is a case-control study design, selected based on exclusion and inclusion criteria.
Blinding	As this is a case-control study, blinding is not applicable.

Reporting for specific materials, systems and methods

We require information from authors about some types of materials, experimental systems and methods used in many studies. Here, indicate whether each material, system or method listed is relevant to your study. If you are not sure if a list item applies to your research, read the appropriate section before selecting a response.

Materials & experimental systems

n/a	Involved in the study	
☒	☐	Antibodies
☒	☐	Eukaryotic cell lines
☒	☐	Palaeontology and archaeology
☒	☐	Animals and other organisms
☐	☒	Human research participants
☒	☐	Clinical data
☒	☐	Dual use research of concern

Methods

n/a	Involved in the study	
☒	☐	ChIP-seq
☒	☐	Flow cytometry
☒	☐	MRI-based neuroimaging

Human research participants

Policy information about studies involving human research participants

Population characteristics	All the study participants were between age group 21-65, with a matched male-female ratio. The comorbidity status of each individual, i.e the Charlson Comorbidity Index (CCI) was described in the manuscript.
Recruitment	Cases were RT-PCR positive COVID-19 patients and controls were defined as RT-PCR, antibody titre negative for SARS-CoV-2 and devoid of any flu-like symptoms. Cases and controls were collected from the same geographical location viz. the campus of ICMR-RMRC-Bhubaneswar and they were matched for the socioeconomic and ethnic background.
Ethics oversight	National Institute of Biomedical Genomics, Kalyani and ICMR-Regional Medical Research Centre, Bhubaneswar.

Note that full information on the approval of the study protocol must also be provided in the manuscript.
